# Selective *E* to *Z* isomerization of 1,3-Dienes Enabled by A Dinuclear Mechanism

**DOI:** 10.1038/s41467-021-21720-4

**Published:** 2021-03-05

**Authors:** Eiji Kudo, Kota Sasaki, Shiori Kawamata, Koji Yamamoto, Tetsuro Murahashi

**Affiliations:** 1 Department of Chemical Science and Engineering, School of Materials and Chemical Technology, Tokyo Institute of Technology, Tokyo, Japan; 2 Research Center of Integrative Molecular Science (CIMoS), Institute for Molecular Science, Okazaki, Aichi Japan

**Keywords:** Synthetic chemistry methodology, Chemical bonding, Stereochemistry

## Abstract

The *E*/*Z* stereocontrol in a C=C bond is a fundamental issue in olefin synthesis. Although the thermodynamically more stable *E* geometry is readily addressable by thermal *Z* to *E* geometric isomerization through equilibrium, it has remained difficult to undergo thermal geometric isomerization to the reverse *E* to *Z* direction in a selective manner, because it requires kinetic trapping of *Z*-isomer with injection of chemical energy. Here we report that a dinuclear Pd^I^−Pd^I^ complex mediates selective isomerization of *E*-1,3-diene to its *Z*-isomer without photoirradiation, where kinetic trapping is achieved through rational sequences of dinuclear elementary steps. The chemical energy required for the *E* to *Z* isomerization can be injected from an organic conjugate reaction through sharing of common Pd species.

## Abstract (web summary)

Geometric *E* to *Z* double C=C bond isomerization is challenging as it requires kinetic trapping of the *Z*-isomer with injection of chemical energy. Here, the authors report a dinuclear Pd(I)−Pd(I) complex that mediates selective isomerization of *E*-1,3-dienes to the *Z*-isomers without photoirradiation.

## Introduction

**Fig. 1:**
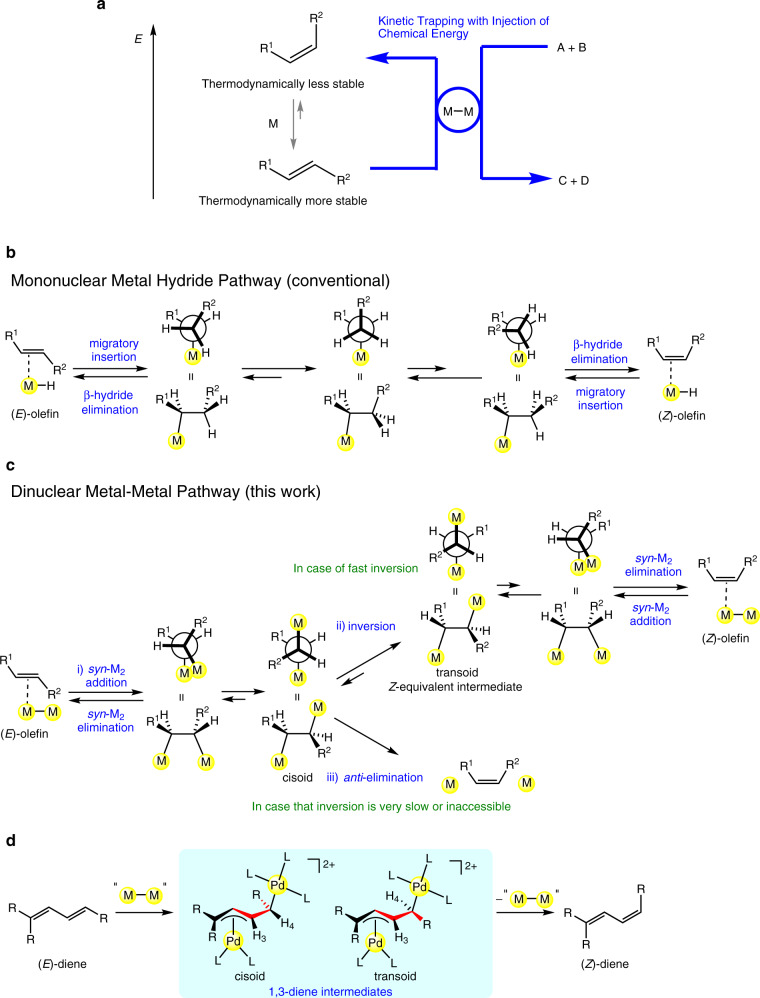
Isomerization of alkenes. **a** A qualitative energy profile of *Z*-alkenes and *E*-alkenes. An M–M complex may promote *E* to *Z* geometric isomerization of alkenes through a dinuclear mechanism. The chemical energy required for the *E* to *Z* isomerization may come from the reaction energy of a coupled reaction (A **+** B **→** C **+** D), where the common M–M species are shared, making the net reaction exergonic. **b** A simplified model for the mononuclear metal hydride pathway which is a representative and conventional mechanism for *Z* to *E* isomerization. **c** A dinuclear M–M pathway that enables kinetic trapping of *Z*-alkene. **d** The key intermediates for the isomerization of 1,3-diene.

The *E*/*Z* stereocontrol of a C**=**C moiety has been a central issue in olefin chemistry. Significant efforts have been made to obtain the thermodynamically less stable *Z*-alkenes as a kinetically preferred product, because *Z*-alkenes are contained in many natural products, biologically active molecules, and synthons for organic synthesis^1,2^. Several bond-construction methods have been developed to obtain *Z*-alkene selectively, such as *syn* 1,2-adition to alkynes, stereoretentive cross-coupling using *Z*-vinyl reagents, *Z*-selective olefin metathesis, modified Wittig reactions, and *Z*-selective double bond migration^3–10^. In view of the fact that an *E*/*Z* mixture of alkenes in which a thermodynamically more stable *E*-alkene is the major isomer can be readily obtained by a common alkene construction method such as the Wittig reaction, the *E* to *Z* geometric isomerization may also become a powerful method to address to *Z*-alkenes. Despite its potential usefulness, however, the *E* to *Z* geometric isomerization of alkenes is not straightforward. Although photoirradiation of several alkenes leads to the *E* to *Z* isomerization^1,11–13^, *Z* stereoselection under photoirradiation is sometimes incomplete, mainly because a photochemical *E*/*Z* ratio depends on a photostationary state derived from an excited state structure^1,14,15^. Moreover, it has been difficult to undergo *E* to *Z* geometric isomerization without photoirradiation, because an equilibrium of a reversible *E*/*Z* isomerization lies largely to the side of *E*-alkenes (Fig. 1a). The claims that thermal *E* to *Z* isomerization of 1,3-dienes proceeds in the presence of a cobalt catalyst^16^ have proven erroneous recently. There has been no rational mechanism that allows kinetic trapping of *Z*-alkenes through a thermal *E*/*Z* isomerization. For example, *E*/*Z* geometric isomerization of alkenes is efficiently mediated or catalyzed by a transition metal hydride ([M]−H)^17^, where the production of *Z*-alkenes is disfavored due to the difficulty of kinetic discrimination of one of two diastereotopic β-H atoms during β-H elimination^10^, in addition to the rapid reversibility of migratory insertion and β-H elimination (Fig. 1b). Any other established mechanisms involving transition metal species such as (allylic C–H oxidative addition)-(reductive elimination), (allylic C–H radical abstraction)-addition, and (nucleophile/electrophile addition)-elimination^18–21^ also preferentially gives *E*-alkene over *Z*-alkene. It is noted that even a technique using 1,2-addition and 1,2-elimination reactions has been considered as a formal *E* to *Z* isomerization, in which an *E*-alkene is initially converted to an isolable C–C single bonded organic product through *syn* (or *anti*) addition, and then 1,2-*anti* (or *syn*) elimination reaction gives a corresponding *Z*-alkene^22–24^. Thus, it is highly desirable to develop a mechanism that enables straightforward, selective *E* to *Z* geometric isomerization of alkenes.

We envisioned that a dinuclear M–M bonded species might promote *E* to *Z* isomerization through a rational mechanism. Our strategy is based on the following hypotheses; i) an M–M bonded species that can accommodate alkenes at its semi-bridging or bridging coordination site undergoes stereoretentive *syn* addition to an *E*-alkene, ii) subsequent stereoinversion at the α-carbon center of the dimetalated intermediate generates the *Z*-equivalent intermediate, that then gives *Z*-alkene selectively via dinuclear *syn* elimination, and iii) as an alternative to ii), dinuclear *anti* elimination from the *syn* addition product gives *Z*-alkene selectively (Fig. 1c). Concerning the pathway i)→ii), the previous stereochemical study by our group proved that the dinuclear addition and elimination of a Pd–Pd moiety to/from 1,3,5-trienes occur in a highly stereoretentive (*syn*) manner, although the stereoinversion in a bi-π-allyl dinuclear adduct is very slow in the absence of Pd^0^ impurities^25^. If the stereoinversion at the α-carbon center occurs reversibly, the *Z*-equivalent intermediate is expected to become a major component because the metal moieties, which are usually the most sterically demanding groups, prefer an antiperiplanar conformation with a transoid R^1^–C–C–R^2^ geometry (Fig. 1c). The pathway i)→iii) may be considered when the stereoinversion at the α-carbon center is very slow or inaccessible, although the selective dinuclear *anti* elimination giving *Z*-alkenes has not been reported. When synthetically important 1,3-dienes are used as the alkene substrate, these mechanisms involve dimetallated intermediates having either η^1^-σ-M or η^3^-π-M (Fig. 1d)^26,27^.

Here, we report that a dinuclear Pd^I^–Pd^I^ complex has the ability to mediate selective isomerization of *E*-1,3-diene to its *Z*-isomer without photoirradiation. We demonstrate that the sequence of dinuclear elementary steps involving either pathway i)→ii) or i)→iii) provides a rational way to obtain *Z*-1,3-dienes. Furthermore, we also show that the chemical energy required for the *E* to *Z* isomerization can be injected from an organic conjugate reaction through sharing of common Pd species, making the net process exergonic (Fig. 1a).

## Results and discussion

### A sequence of dinuclear addition, stereoinversion, and *syn* dinuclear elimination

**Fig. 2:**
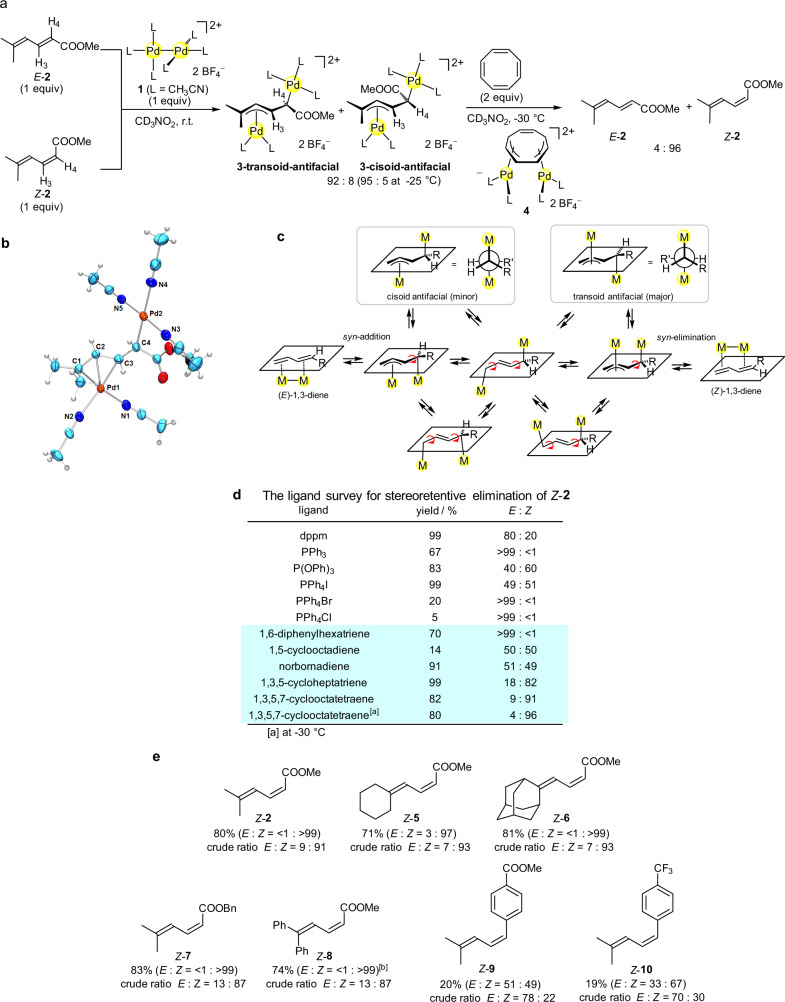
The dinuclear addition of a Pd^I^–Pd^I^ moiety to substituted 1,3-dienes. **a** The dinuclear addition to 5-methylhexa-2,4-dienoate (**2**), and subsequent dinuclear elimination giving *Z*-diene selectively. **b** ORTEP for **3-transoid-antifacial** (30% probability ellipsoids; counteranions are omitted for clarity). **c** The allyl π–σ–π interconversion for the rapid stereoinversion mechanism. **d** The ligand survey for stereoretentive elimination of *Z*-**2**. The reactions were carried out in CD_3_NO_2_ at room temperature. dppm = bis(diphenylphosphino)methane. [a] At −30 °C. **e** Yields of *Z*-1,3-dienes after isolation. Typically, the diene was added to a CH_3_NO_2_ solution of **1**, and stirred for 30 min. The reaction mixture was cooled to −30 °C, and then COT was added and the mixture was stirred for 15 min. [b] PPh_4_I was used instead of COT.

We employed a dinuclear Pd^I^–Pd^I^ bonded complex [Pd_2_(CH_3_CN)_6_][BF_4_]_2_ (**1**)^28^, which shows high reactivity with unsaturated hydrocarbons. We chose 1,3-dienes as the alkene substrate, not only because 1,3-dienes become useful starting substrates for many synthetic applications, but also because 1,3-dienes are smoothly introduced to the bridging coordination site of a dinuclear Pd–Pd^27,29–32^. We found that the reaction of **1** with either methyl *E*-5-methylhexa-2,4-dienoate (*E*-**2**) or its *Z*-isomer (*Z*-**2**) immediately gave an equilibrium mixture of transoid-antifacial and cisoid-antifacial isomers of the dinuclear adducts [Pd_2_(μ-η^3^:η^1^-mmd)(CH_3_CN)_5_][BF_4_]_2_ (**3**, mmd = methyl 5-methylhexa-2,4-dienoate) in a 92:8 molar ratio at 25 °C. The molar ratio of **3-transoid-antifacial**:**3-cisoid-antifacial** reached 95:5 at −25 °C (Fig. 2a). The structures of these isomers were assigned by ^1^H-NMR and ^13^C-NMR analyses, where the vicinal coupling constant *J*_H3–H4_ of the transoid-antifacial isomer (*J*_H3–H4_ **=** 10.4 Hz) is larger than that of cisoid-antifacial one (*J*_H3–H4_ **=** 6.8 Hz), and the η^1^-bound ^13^C atom, which is located at the α-position with respect to the COOMe group, appeared at the high-field region (*δ* = 31 ppm for **3-transoid-antifacial**). The molecular structure of the major isomer **3-transoid-antifacial** was confirmed by X-ray structure analysis (Fig. 2b). The η^3^-bound Pd1 atom and the η^1^-bound Pd2 atom are antiperiplanar with each other, and the conformation around the C3–C4 bond is transoid. The C3–C4 bond length (1.446(12) Å) is longer than those of C1–C2 (1.413(11) Å) and C2–C3 (1.385(11) Å) due to its single bond character. The minor production of **3-cisoid-antifacial** after the dinuclear addition to *E*-**2** indicated that the stereoinversion of the dinuclear adduct **3** proceeded rapidly. That is, the *syn* dinuclear addition of **1** with *E*-**2** initially gives **3-cisoid-antifacial**, and subsequent stereoinversion occurs through the π–σ–π interconversion of the η^3^-allyl Pd moiety^33,34^, giving the thermodynamically more stable **3-transoid-antifacial** in rapid equilibrium (Fig. 2c). As mentioned below, it was confirmed that the dinuclear addition of 1,3-diene to the Pd–Pd moiety proceeds in a stereoretentive (*syn*) manner when employing a 1,4-disubstituted 1,3-diene as the reactant. The theoretical calculations supported the relative stability of the dinuclear addition products; i.e., **3-transoid-antifacial** is more stable by Δ*G* **=** 6.9 kJ/mol than **3-cisoid-antifacial**; cf. *Z*-**2** is in higher energy by Δ*G* **=** 8.3 kJ/mol compared to *E*-**2**.

We then examined the kinetic trapping of *Z*-**2** from **3-transoid-antifacial** through *syn* elimination (Fig. 2d), although the rapid π–σ–π allyl-Pd interconversion can cause elimination of *E*-**2** via the reverse way of the formation of **3-transoid-antifacial** from *E*-**2**. Bis(diphenylphoshino)methane (dppm) has been used as a good dinuclear eliminating reagent owing to its bridging coordination ability^25,35^, but addition of dppm to **3-transoid-antifacial** showed poor *Z*-selectivity (*E*:*Z* = 80:20). Addition of PPh_3_ gave only *E*-**2**. P(OPh)_3_ showed improved but moderate *Z*-selectivity. Iodide also induced elimination of **2** with a moderate *Z*-selectivity (*E*:*Z* = 49:51), while 1,3-diene elimination with chloride or bromide was sluggish. We then examined the trans-olefination. Norton et al. previously revealed that the reversible trans-olefination of the diosmacyclobutane complex Os_2_(μ-η^1^:η^1^-C_2_H_4_)(CO)_8_ proceeds in a stereoretentive manner^36,37^. Our group also reported that the trans-olefination of the μ-η^3^:η^3^-1,3,5-triene Pd_2_ adduct with a linear 1,3,5-triene proceeds in a stereoretentive manner^25^. Although initial attempts of trans-olefination using 1,6-diphenyl-1,3,5-hexatriene gave only *E*-**2**, the use of 1,3,5-cycloheptatriene gave much better *Z*-selectivity (*E*:*Z* = 18:82). The trans-olefination with 1,3,5,7-cyclooctatetraene (COT) appeared to give a nearly perfect *Z*-selectivity (*E*:*Z* = 4:96) at −30 °C from the 95:5 mixture of the dimetallated intermediate **2**. The ^1^H NMR analysis showed that [Pd_2_(μ-η^3^:η^3^-COT)(CH_3_CN)_4_][BF_4_]_2_ (**4**)^38^ was formed after the trans-olefination with COT. By using the present method, the *Z*-isomer of **2** and several other 1,3-dienes **5**-**8** having an ester group at one of the diene termini were obtained conveniently in 71–83% yields from the corresponding pure *E*-isomer (Fig. 2e). Typically, *Z*-**2** was obtained by treatment of *E*-**2** with **1** for 30 min at room temperature in CH_3_NO_2_ and subsequent addition of COT (2 equiv) at −30 °C for 15 min, where the Pd complex **4** can be easily removed by filtration after pouring the reaction mixture into Et_2_O, gave a crude product containing *E*-**2**:*Z*-**2** = 9:91. It is noted that the observed high *Z*-selectivity is in contrast to the relatively lower selectivity of a previously reported photoinduced isomerization of **2**; e.g., *E*-**2**:*Z*-**2** = 40:60 from *E*-**2** after irradiation with ultraviolet (UV)-light (254 nm)^39^. By using the present method, terminally aryl substituted dienes *Z*-**9** and *Z*-**10** were formed in low *Z*-selectivity (Fig. 2e) probably due in part to the concomitant formation of more than one dinuclear addition product; i.e., we observed unidentified isomers of the dinuclear adducts for these dienes by ^1^H NMR monitoring experiments.

### A sequence of *syn* dinuclear addition and anti-dinuclear elimination

**Fig. 3:**
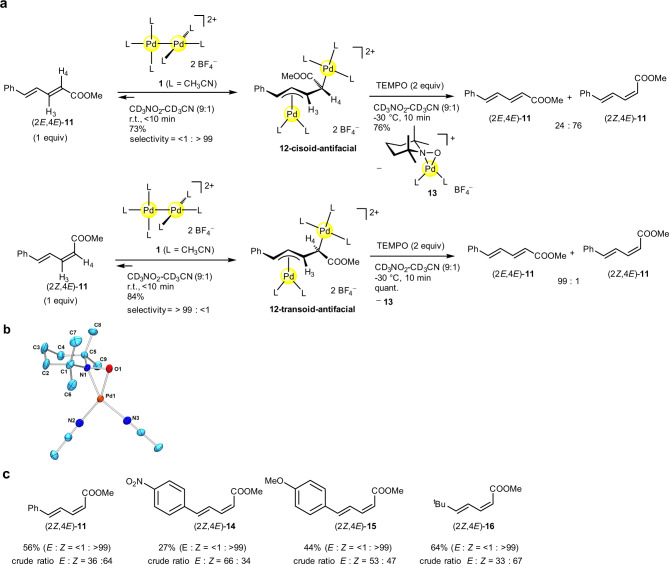
Isomerization of (*E*,*E*)-diene to (*Z*,*E*)-diene. **a** Reaction scheme to access (2*Z*,4*E*)-isomer of methyl 5-phenylpenta-2,4-dienoate (**11**) through geometric isomerization. **b** ORTEP for **13** (50% probability ellipsoids; counter anion is omitted for clarity). **c** Yields of (2*Z*,4*E)*-1,3-dienes from the corresponding (2*E,4E*)-1,3-dienes. Typically, the diene was added to a solution of **1** in CH_3_NO_2_/CH_3_CN (*v*/*v* **=** 95:5) at room temperature, and stirred for 15 min. The reaction mixture was then cooled to −20 °C and a solution of TEMPO (4 equiv) was added and stirred for 5 min.

According to the mechanism shown in Fig. 2a, the *E* to *Z* isomerization of 1,4-disubstituted 1,3-dienes is not straightforward, because the corresponding dinuclear addition intermediate unlikely undergoes stereoinversion through π–σ–π allyl interconversion that accompanies the exchange of the *syn*/*anti*-substituents at the terminal η^3^-allyl moieties. In fact, it was confirmed that addition of **1** to the (2*E*,4*E*)- or the (2*Z*,4*E*)-isomer of methyl 5-phenylpenta-2,4-dienoate (**11**) in a CD_3_NO_2_-CD_3_CN solution (*v*/*v* = 9/1) proceeded in a highly stereospecific (*syn*) manner, yielding the transoid-antifacial or cisoid-antifacial dinuclear adduct [Pd_2_(μ-η^3^:η^1^-mpd)L_5_][BF_4_]_2_ (**12-cisoid-antifacial** or **12-transoid-antifacial**, mpd **=** methyl 5-phenylpenta-2,4-dienoate) (Fig. 3a). The structure of each isomer of **12** was identified by the ^1^H NMR analysis, where the vicinal H3–H4 coupling constant of the transoid isomer (*J*_H3–H4_ = 10.4 Hz) is larger than that of the cisoid isomer (*J*_H3–H4_ = 6.0 Hz). We then examined the direct *anti* elimination from the *E*-equivalent intermediate **12-cisoid-antifacial** that may give (2*Z*,4*E*)-**11**. For the *anti* elimination, we focused on the free-radical-induced metal elimination, since it is known that a metal–carbon bond undergoes homolysis by addition of radical species^40–42^. The double homolysis of the C–Pd bonds proceeded by addition of 2,2,6,6-tetramethylpiperidine 1-oxyl (TEMPO) (2 equiv) to **12-cisoid-antifacial** in CD_3_NO_2_-CD_3_CN (*v*/*v* = 9/1) at −30 °C, giving (2*Z*,4*E*)-**11** (76% yield, (2*E*,4*E*)-**11**:(2*Z*,4*E*)-**11** = 24:76), where the mononuclear TEMPO-adduct [Pd(η^2^-TEMPO)(CH_3_CN)_2_][BF_4_] (**13**) was concomitantly formed (Fig. 3a). The TEMPO adduct **13** was isolated upon treatment of **1** with TEMPO in 91% yield, and its structure was determined by X-ray structure analysis (Fig. 3b). The observed *Z*-selectivity indicated that association of TEMPO to each Pd^II^ center in **12-cisoid-antifacial** and subsequent Pd–C bond cleavage occurs with conservation of the cisoid geometry. The partial loss of the stereochemistry may be caused by the TEMPO-induced elimination mechanism, where a palladium moiety could eliminate in a stepwise manner. We confirmed that the treatment of **12-transoid-antifacial** with TEMPO gave (2*E*,4*E*)-**11** selectively (Fig. 3a). It is noted that treatment of **12-transoid-antifacial** with COT resulted in the quantitative recovery of (2*Z*,4*E*)-**11**. For the preparative scale, in-situ-generated **12-cisoid-antifacial** in CH_3_NO_2_–CH_3_CN (*v*/*v* = 95/5) from pure (2*E*,4*E*)-**11** was treated with TEMPO (4 equiv) at −20 °C to give (2*Z*,4*E*)-**11** in 56% yield (Fig. 3c). Other 1,4-disubstituted (2*E*,4*E*)-dienes such as **14**, **15**, and **16** were also isomerized to the corresponding (2*Z*,4*E*)-isomers in 27–64% yields (Fig. 3c). It was reported that UV-irradiation of (2*E*,4*E*)-**11** at 313 nm gives a mixture of possible four isomers (2*E*,4*E*), (2*Z*,4*E*), (2*E*,4*Z*), and (2*Z*,4*Z*)-**11** with low selectivity (18:16:34:32)^39^.

### Injection of chemical energy through conjugate reactions

**Fig. 4:**
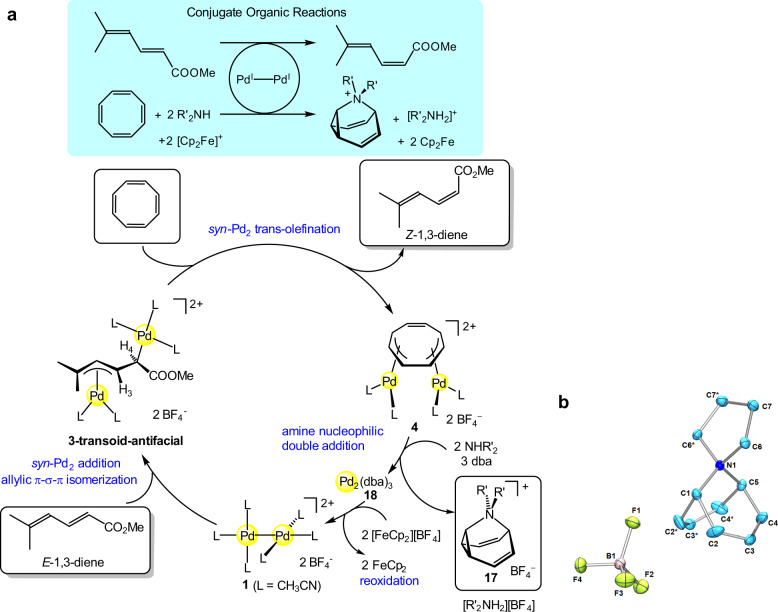
The conjugate reaction system for the uphill *E* to *Z* isomerization. **a** *E* to *Z* isomerization of 1,3-diene is energetically coupled with oxidative double amination of cyclooctatetraene. The reaction conditions: CH_3_CN, 0 °C, 2 h, for **4** to **18**; CH_3_CN/CH_2_Cl_2_, r.t., 1 h for **18** to **1**. **b** ORTEP for **17** (30% probability ellipsoids).

Finally, we demonstrated that the concept of conjugate reactions is applicable to the present *E* to *Z* isomerization of 1,3-diene, where the required chemical energy is injected from a coupled reaction by sharing of a common metal species (Fig. 1a). In biological systems, many endergonic chemical reactions are operated by energetic coupling with an exergonic reaction such as ATP-hydrolysis, that makes the net reaction system exergonic^43^. However, it is rare that the concept of the conjugate reaction system is applied to the reaction-design of artificial metal mediated-hill or catalyzed up-hill organic transformations. Although the above mentioned *E* to *Z* isomerization of **2** is driven thermodynamically by the organometallic complexation reaction of **1** with COT to yield the complex **4**, a conjugate reaction system in which the *E* to *Z* isomerization is energetically coupled with a downhill reaction (A + B → C + D in Fig. 1a) could give a net exergonic system. Furthermore, regeneration of **1** after the coupled down-hill reaction is highly desirable. We developed the oxidative double amination of COT by using **1** for the organic coupled reaction. The reaction of **4** with a secondary amine such as pyrrolidine (2 equiv) at 0 °C in the presence of dibenzylideneacetone (dba) (5 equiv) gave a disubstituted 9-aza-barbaralane (**17**)^44^ (58% yield) and Pd_2_(dba)_3_ (**18**)^45–47^ (71% yield) (Fig. 4a). The molecular structure of the fluxional molecule **17** was confirmed by X-ray structure analysis (Fig. 4b). The formation of **17** might involve the nucleophilic amine-attack at one of the η^3^-allyl moieties in **4**, subsequent deprotonation, intramolecular nucleophilic attack at the central carbon of the remaining η^3^-allyl moiety, and reductive elimination. The resultant Pd^0^ complex **18** can be converted to the Pd^I^–Pd^I^ complex **1** in 90% yield by treatment with [Cp_2_Fe][BF_4_] in CH_3_CN–CH_2_Cl_2_ at room temperature (Fig. 4a), although the selective synthesis of a Pd^I^_2_ complex by one-electron oxidation of Pd^0^ or one-electron reduction of Pd^II^ has been rarely reported^48^. Thus, merging the up-hill *E* to *Z* isomerization of 1,3-diene and the downhill oxidative double amination of COT gave a net exergonic conjugate reaction system, where delivery of the Pd^I^–Pd^I^ species or its equivalent from one reaction to another gives a closed cycle (Fig. 4a).

For the *anti* elimination mechanism, the downhill oxidative protonation reaction of TEMPO becomes the coupled reaction for the up-hill *E* to *Z* alkene isomerization of **11**. That is, protonation of the TEMPO-adduct **13** with HBF_4_·Et_2_O in CH_3_CN afforded [Pd(CH_3_CN)_4_][BF_4_]_2_ (**19**)^49^ in 80% yield with the elimination of [TEMPOH_2_][BF_4_] (**20**). The Pd^II^ complex **19** was then reduced with Cp_2_Fe in CH_3_CN to give **1** in 97% yield.

In summary, we have described *E* to *Z* isomerization of 1,3-dienes mediated by the dinuclear M–M bonded species. The *E* → *Z* stereocontrol can be achieved by a rational dinuclear mechanism that allows kinetic trapping of *Z*-1,3-diene. Furthermore, the concept of the conjugate reaction system demonstrated in this work may provide a basis to promote the metal-mediated up-hill organic transformation through energetic coupling with an exergonic reaction.

## Methods

### General procedure

All manipulations were conducted under a nitrogen atmosphere using standard Schlenk or drybox techniques. Unless specified, all reagents were purchased from commercial suppliers and used without purification. Solvents were purified according to the standard procedures. For experimental details, spectroscopic characterization data, X-ray crystallographic data, and details of quantum calculations, see the Supplementary Information.

### Isomerization of methyl *E*-2 to *Z*-2

To a solution of [Pd_2_(CH_3_CN)_6_][BF_4_]_2_ (**1**, 126.5 mg, 2.00 × 10^−1^ mmol) in CH_3_NO_2_ was added methyl (*E*)-5-methylhexa-2,4-dienoate (*E*-**2**, 28.0 mg, 2.00 × 10^−1^ mmol) and stirred at room temperature for 30 min. The reaction mixture was cooled to −30 °C and COT (41.7 mg, 4.00 × 10^−1^ mmol, 2 equiv.) was added. After stirring 15 min, the mixture was diluted with diethyl ether, and filtered through a silica gel pad. The solvent was removed in vacuo and the residue was purified by a silica gel column chromatography to give methyl (*Z*)-5-methylhexa-2,4-dienoate (*Z*-**2**) as colorless oil (22.4 mg, 1.60 × 10^−1^ mmol, 80% yield, *E*:*Z* = <1:>99).

### Isomerization of (2*E*,4*E*)-11 to (2*Z*,4*E*)-11

To a solution of [Pd_2_(CH_3_CN)_6_][BF_4_]_2_ (**1**, 168.1 mg, 2.66 × 10^−1^ mmol) in CH_3_NO_2_/CH_3_CN (*v*/*v* = 95:5) was added methyl (2*E*,4*E*)-5-phenylpenta-2,4-dienoate ((2*E*,4*E*)-**11**) (50.0 mg, 2.66 × 10^−1^ mmol) and stirred at room temperature for 15 min. The reaction mixture was cooled to −20 °C and a CH_3_NO_2_/CH_3_CN (*v*/*v* = 95:5) solution of TEMPO (167.7 mg, 1.07 mmol, and 4 equiv.) was added at −20 °C. After stirring 5 min, the mixture was diluted with diethyl ether, and filtered through Celite. The solution was washed with distilled water (two times). The organic layer was collected, and the aqueous layer was extracted with Et_2_O (two times). The combined organic layers were dried with MgSO_4_. The solvent was removed in vacuo and the residue was purified by a silica gel column chromatography and recycling size exclusion chromatography to give methyl (2*Z*,4*E*)-5-phenylpenta-2,4-dienoate ((2*Z*,4*E*)-**11**) as colorless oil (28.2 mg, 1.50 × 10^−1^ mmol, 56% yield, *E*:*Z* = <1:>99).

### Synthesis of Pd_2_(dba)_3_ and 9-aza-barbaralane 17 through double amination of 4

To a solution of [Pd_2_(CH_3_CN)_6_][BF_4_]_2_ (**1**, 100.0 mg, 1.60 × 10^−1^ mmol) in CH_3_CN was added the COT (17.3 mg, 1.70 × 10^−1^ mmol) and stirred at room temperature. After 30 min, dba (185.1 mg, 7.90 × 10^−1^ mmol) was added to the reaction mixture, and then the reaction mixture was cooled at 0 °C. After addition of pyrrolidine (22.4 mg, 3.20 × 10^−1^ mmol) at 0 °C, the reaction mixture was stirred at the temperature for 2 h. The violet precipitate in suspension was then separated by decantation, and the supernatant was concentrated in vacuo. The solution was diluted with CHCl_3_, and filtered through a silica gel pad. The silica gel pad was washed with CHCl_3_. The product was extracted by MeOH/CHCl_3_ (*v*/*v* = 1:5), and concentrated in vacuo. The crude product was purified by a silica gel column chromatography (MeOH/CHCl_3_ (*v*/*v* = 1:10)) to give 9-aza-barbaralane **17** (24.2 mg, 9.00 × 10^−2^ mmol, 58% yield**)** as a red solid. The single crystal of **17** suitable for X-ray diffraction analysis was obtained by recrystallization from CH_2_Cl_2_/diethyl ether at −30 °C. The violet precipitate separated by decantation was washed with CH_3_CN and diethyl ether and dried in vacuo to yield Pd_2_(dba)_3_ (**18**) in 71% yield (Yield was determined by the free dba/Pd_2_(dba)_3_ molar ratio from ^1^H NMR).

## Supplementary information

Supplementary Information

## Data Availability

The authors declare that the data supporting the findings of this study are available within the paper and its supplementary information files, as well as from the corresponding author upon reasonable request. The X-ray crystallographic coordinates for structures reported in this study (**3-transoid-antifacial**, **13**, and **17**) have been deposited at the Cambridge Crystallographic Data Centre (CCDC), under deposition numbers 2003060-2003062. These data can be obtained free of charge from The Cambridge Crystallographic Data Centre via www.ccdc.cam.ac.uk/data_request/cif. The experimental data are available in Supplementary Information.

## References

[1] Molloy JJ, Morack T, Gilmour R (2019). Positional and geometrical isomerisation of alkenes: the pinnacle of atom economy. Angew. Chem. Int. Ed..

[2] Siau W-Y, Zhang Y, Zhao Y (2012). Stereoselective synthesis of *Z*-alkenes. Top. Curr. Chem..

[3] Oger C, Balas L, Durand T, Galano J-M (2013). Are alkyne reductions chemo-, regio-, and stereoselective enough to provide pure (*Z*)-olefins in polyfunctionalized bioactive molecules?. Chem. Rev..

[4] Miyaura N, Suzuki A (1995). Palladium-catalyzed cross-coupling reactions of organoboron compounds. Chem. Rev..

[5] Negishi E (2008). Recent advances in efficient and selective synthesis of di-, tri-, and tetrasubstituted alkenes via Pd-catalyzed alkenylation-carbonyl olefination synergy. Acc. Chem. Res..

[6] Meek SJ, O’Brien RV, Llaveria J, Schrock RR, Hoveyda AH (2011). Catalytic *Z*-selective olefin cross-metathesis for natural product synthesis. Nature.

[7] Montgomery TP, Ahmed RS, Grubbs RH (2017). Stereoretentive olefin metathesis: an avenue to kinetic selectivity.. Angew. Chem. Int. Ed..

[8] Still WC, Gennari C (1983). Direct synthesis of *Z*-unsaturated esters. A useful modification of the Horner-Emmons olefination. Tetrahedron Lett..

[9] Ando K, Oishi T, Hirama M, Ohno H, Ibuka T (2000). *Z*-selective Horner-Wadsworth-Emmons reaction of ethyl (diarylphosphono)acetates using sodium iodide and DBU. J. Org. Chem..

[10] Holland PL (2015). Distinctive reaction pathways at base metals in high-spin organometallic catalysts. Acc. Chem. Res..

[11] Singh K, Staig S, Weaver JD (2014). Facile synthesis of *Z*-alkenes via uphill catalysis.. J. Am. Chem. Soc..

[12] Metternich JB, Gilmour R (2015). A bio-inspired, catalytic *E* → *Z* isomerization of activated olefins. J. Am. Chem. Soc..

[13] Molloy JJ (2020). Boron-enabled geometric isomerization of alkenes via selective energy-transfer catalysis. Science.

[14] Meier H (1992). The photochemistry of stilbenoid compounds and their role in materials technology.. Angew. Chem. Int. Ed..

[15] Arai T, Tokumaru K (1993). Photochemical one-way adiabatic isomerization of aromatic olefins. Chem. Rev..

[16] Pünner F, Schmidt A, Hilt G (2019). Corrigendum: Up the Hill: selective double‐bond isomerization of terminal 1,3‐dienes towards *Z*‐1,3‐dienes or 2*Z*,4*E*‐dienes. Angew. Chem. Int. Ed..

[17] Larionov E, Li H, Mazet C (2014). Well-defined transition metal hydrides in catalytic isomerizations. Chem. Commun..

[18] Cramer R, Lindsey RV (1966). The mechanism of isomerization of olefins with transition metal catalysts. J. Am. Chem. Soc..

[19] Kapat A, Sperger T, Guven S, Schoenebeck F (2019). *E*-olefins through intramolecular radical relocation. Science.

[20] Tan EHP, Lloyd-Jones GC, Harvey JN, Lennox AJJ, B. M. Mills BM (2011). [(RCN)_2_PdCl_2_]-catalyzed *E*/*Z* isomerization of alkenes: a non-hydride binuclear addition-elimination pathway.. Angew. Chem. Int. Ed..

[21] Sen A, Lai T-W (1981). Catalytic isomerization of alkenes by palladium(II) compounds. An alternative mechanistic view.. Inorg. Chem..

[22] Vedejs E, Fuchs PL (1973). Inversion of acyclic olefins by the phosphorus betaine method: scope and limitations. J. Am. Chem. Soc..

[23] Lamb JR, Hubbell AK, MacMillan SN, Coates GW (2020). Carbonylative, catalytic deoxygenation of 2,3-disubstituted epoxides with inversion of stereochemistry: an alternative alkene isomerization method. J. Am. Chem. Soc..

[24] Maeda K, Shinokubo H, Oshima K (1996). Olefin inversion: stereospecific olefin synthesis from vicinal alkoxyiodoalkanes with butyllitium by an E2 syn mechanism. J. Org. Chem..

[25] Murahashi T (2006). Stereoretentive elimination and trans-olefination of the dicationic dipalladium moiety [Pd_2_L_*n*_]^2+^ bound on 1,3,5-trienes. J. Am. Chem. Soc..

[26] Kreiter CG, Lipps W (1981). Cleavage of a metal-metal bond by 1,3-butadiene under photochemical conditions.. Angew. Chem. Int. Ed. Engl..

[27] Murahashi T, Nagai T, Nakashima H, Tomiyasu S, Kurosawa H (2006). Dinuclear addition of the Pd–Pd moieties to 1,3-dienes.. Chem. Lett.

[28] Murahashi, T., Nagai, T., Okuno, T., Matsutani, T. & Kurosawa, H. Synthesis and ligand substitution reaction of homoleptic acetonitrile dipalladium(I) complex. *Chem. Commun*. 1689–1690 (2000).

[29] Leoni P (1993). Reaction of phosphide-bridged palladium(I) dimers containing secondary phosphines with ethylene and isoprene: coordination vs. insertion. Organometallics.

[30] Murahashi, T., Kanehisa, N., Kai, Y., Otani, T. & Kurosawa, H. Rational synthesis of anionic, neutral, and cationic palladium(I) dinuclear complexes containing bridging conjugated dienes. *Chem. Commun*. 825–826 (1996).

[31] Murahashi T, Otani T, Mochizuki E, Kai Y, Kurosawa H (1998). Remarkably wide range of bond distance adjustment of d^9^-d^9^ Pd-Pd interactions to change in coordination environment. J. Am. Chem. Soc..

[32] Lin S, Herbert DE, Velian A, Day MW, Agapie T (2013). Dipalladium(I) terphenyl diphosphine complexes as models for two-site adsorption and activation of organic molecules. J. Am. Chem. Soc..

[33] Solin N, Szabo KJ (2001). Mechanism of the η^3^-η^1^-η^3^ isomerization in allylpalladium complexes: solvent coordination, ligand, and substituent effects. Organometallics.

[34] Ogasawara M, Takizawa K, Hayashi T (2002). Effects of bidentate phosphine ligands on *syn*-*anti* isomerization in π-allylpalladium complexes. Organometallics.

[35] Holloway, R. G., Penfold, B. R., Colton, R., McCormick, M. J. Crystal and molecular structure of bis-μ-(bisdiphenylphosphinomethane)-dibromodipalladium(*Pd*-*Pd*), a compound containing palladium(I). *J. Chem. Soc., Chem. Commun*. 485–486 (1976).

[36] Ramage DL, Wiser DC, Norton JR (1997). Kinetics of diosmacyclobutane exchange reactions. J. Am. Chem. Soc..

[37] Bender BR, Ramage DL, Norton JR, Wiser DC, Rappé AK (1997). Evidence for a ring-opening preequilibrium in the exchange reaction of diosmacyclobutanes. J. Am. Chem. Soc..

[38] Murahashi T, Kato N, Ogoshi S, Kurosawa H (2008). Synthesis and structure of dipalladium complexes containing cyclooctatetraene and bicyclooctatrienyl ligands. J. Organomet. Chem..

[39] Lewis FD, Howard DK, Barancyk SV, Oxman JD (1986). Lewis acid catalysis of photochemical reactions. 5. Selective isomerization of conjugated butenoic and dienoic esters. J. Am. Chem. Soc..

[40] Poli, R. Radical coordination chemistry and its relevance to metal-mediated radical polymerization. *Eur. J. Inorg. Chem*. 1513–1530 (2011).

[41] Reid, S. J. & Baird, M. C. Reactions of free radicals with η^3^-allylpalladium(II) complexes: phenyl and trityl radicals. *Dalton Trans*. 3975–3980 (2003).

[42] Boisvert L, Denney MC, Hanson SK, Goldberg KI (2009). Insertion of molecular oxygen into a palladium(II) methyl bond: a radical chain mechanism involving palladium(III) intermediates. J. Am. Chem. Soc..

[43] Peusner, L. *Concepts in Bioenergetics* (Prentice Hall, 1974).

[44] Anastassiou AG, Reichmainis E, Winston AE (1976). Unsubstituted 9-azabarbaralane; a π-destabilized heterocycle. Angew. Chem. Int. Ed. Engl..

[45] Takahashi, Y., Ito, T., Sakai, S. & Ishii, Y. A novel palladium(0) complex; bis(dibenzylideneacetone)palladium(0). *Chem. Commun*. 1065–1066 (1970).

[46] Mazza, M. C. & Pierpont, C. G. Structure and bonding in tris(dibenzylideneacetone)dipalladium(0). *Chem. Commun*. 207–208 (1973).

[47] Kapdi AR (2013). The elusive structure of Pd_2_(dba)_3_. Examination by isotopic labeling, NMR spectroscopy, and X-ray diffraction analysis: synthesis and characterization of Pd_2_(dba-Z)_3_ complexes. J. Am. Chem. Soc..

[48] Johansson Seechurn CCC, Sperger T, Scrase TG, Schoenebeck F, Colacot T (2017). Understanding the unusual reduction mechanism of Pd(II) to Pd(I): uncovering hidden species and implications in catalytic cross-coupling reactions.. J. Am. Chem. Soc..

[49] Schramm, R. F. & Wayland, B. B. Oxidation of metallic palladium by nitrosyl tetrafluoroborate. *Chem. Commun*. 898–899 (1968).

