# Supplementary material for: Selective E to Z isomerization of 1,3-Dienes Enabled by A Dinuclear Mechanism
Source: Nat Commun. 2021 Mar 5;12:1473. doi: 10.1038/s41467-021-21720-4 (PMC7935995; doi:10.1038/s41467-021-21720-4)
Supplement: Supplementary file 1 — Supplementary Information [file 41467_2021_21720_MOESM1_ESM.pdf]

Supplementary Information

Selective Thermal *E* to *Z* Isomerization of 1,3-Dienes Enabled by A Dinuclear Mechanism

Eiji Kudo, Kota Sasaki, Shiori Kawamata, Koji Yamamoto, & Tetsuro Murahashi

Table of Contents

Supplementary Methods	3
General Methods	3
Generation of $[Pd_2(\mu\text{-}\eta^3{:}\eta^1\text{-mmd})(CH_3CN)_5][BF_4]_2$ (**3**)	4
Preparation of *E*-dienes	6
General procedure for elimination of diene **2** from complex **3**	24
E to *Z* isomerization of dienes through *syn*-elimination of transoid-antifacial-Pd_2 complexes by using COT	24
E to *Z* isomerization of methyl (*E*)-5,5-diphenylpenta-2,4-dienoate by using PPh_4I	28
Synthesis of methyl (2*Z*,4*E*)-5-phenylpenta-2,4-dienoate ((2*Z*,4*E*)-**11**)	36
Generation of $[Pd_2(\mu\text{-}\eta^3{:}\eta^1\text{-mpd})(CH_3CN)_5][BF_4]_2$ (**12**) from diene **11**	38
Anti-elimination of diene **11** from complex **12** by the addition of TEMPO	41
Isolation of $[Pd(\eta^2\text{-TEMPO})(CH_3CN)_2][BF_4]$ (**13**)	41
E to *Z* isomerization of dienes through *anti*-elimination of transoid-antifacial-Pd_2 complexes by using TEMPO	43
Synthesis of $Pd_2(dba)_3$ and 9-aza-barbaralane **17** through double amination of complex **4**	48
Synthesis of complex **1** through the oxidation of $Pd_2(dba)_3$ (**18**)	50
Synthesis of $[Pd(CH_3CN)_4][BF_4]_2$ (**19**) by protonation of TEMPO-adduct **13**	50
Synthesis of $[Pd_2(CH_3CN)_6][BF_4]_2$ (**1**) by reduction of $[Pd(CH_3CN)_4][BF_4]_2$ (**19**)	50
Computational Details	51
X-ray Crystallographic Analyses	61
X-ray Crystallographic Data	61
Supplementary References	68

Supplementary Methods

General methods

All manipulations involving air- and moisture-sensitive compounds were conducted under a nitrogen atmosphere using standard Schlenk technique or glove-box technique. ^{1}H (400 MHz) and ^{13}C (101 MHz) NMR spectra were recorded on a JEOL JNM-ECZ400S instrument. The chemical shifts were referenced to the residual resonances of deuterated solvents. Assignments for ^{1}H and ^{13}C NMR signals for some of compounds were aided by 2D ^{1}H-^{1}H COSY, 2D ^{1}H-^{13}C HSQC, and 2D ^{1}H-^{13}C HMBC spectra. Elemental analyses were performed on a PerkinElmer 2400II series CHN analyzer. X-ray crystal data were collected by a Rigaku RAXIS-RAPID imaging plate or a Rigaku Saturn CCD diffractometer with graphite-monochromated Mo-Kα (0.71075 Å) radiation. ESI-MS spectra were recorded on Bruker micrOTOF. HRMS(EI) spectra were recorded on a JEOL JMS-700 instrument at the center for advanced materials analysis, technical department, Tokyo Institute of Technology. Unless specified, all reagents were purchased from commercial suppliers and used without purification. Acetonitrile, dichloromethane, diethyl ether, *n*-hexane, tetrahydrofuran, CD_3CN, CD_2Cl_2, CD_3NO_2, and tetrahydrofuran-d_8 were purified according to the standard procedures. $[Pd_2(CH_3CN)_6][BF_4]_2$ (**1**),[1] $Pd_2(dba)_3{\cdot}CHCl_3$,[2] $Pd(norbornene)_3$,[3] $[Cp_2Fe][BF_4]$,[4] methyl (*E*)-5-methylhexa-2,4-dienoate (*E*-**2**),[5] methyl (*E*)-4-(dialkoxylphosphono)but-2-enoate,[6] methyl 2-(dimethoxyphosphoryl)acetate,[7] dimethyl (arylmethyl)phosphonate,[8] and methyl (2Z)-3-iodoprop-2-enoate[9] were prepared according to the literature.

Generation of $[Pd_2(\mu\text{-}\eta^3{:}\eta^1\text{-mmd})(CH_3CN)_5][BF_4]_2$ (**3**)

To a solution of $[Pd_2(CH_3CN)_6][BF_4]_2$ (**1**, 10.0 mg, 1.60 x 10^{-2} mmol) in CD_3NO_2 was added methyl (*E*)-5-methylhexa-2,4-dienoate (*E*-**2**) (2.2 mg, 1.6 x 10^{-2} mmol). After 15 min at room temperature, the ^{1}H NMR spectra showed the generation of **3-transoid-antifacial** and **3-cisoid-antifacial** in 91% and 8% yield, respectively. The yellow single crystals of **3-transoid-antifacial** were grown from CH_3CN-CH_2Cl_2-toluene solution at -30 ˚C.

For **3-transoid-antifacial**, ^{1}H NMR (400 MHz, 25 ˚C, CD_3NO_2): δ 5.59 (d, ^3J = 12.4 Hz, 1H, H_4), 4.56 (dd, ^3J = 12.4 Hz, 10.4 Hz, 1H, H_3), 4.06 (d, ^3J = 10.4 Hz, 1H, H_2), 3.74 (s, 3H, OMe), 1.54 (s, 3H, Me), 1.41 (s, 3H, Me). ^{13}C NMR (101 MHz) δ 174.9 (C_1), 110.0 (C_4), 97.9 (C_5), 80.6 (C_3), 52.8 (OMe), 31.4 (C_2), 27.3 (Me), 22.3 (Me).

For **3-cisoid-antifacial**, ^{1}H NMR (400 MHz, 25 ˚C, CD_3NO_2): δ 5.97 (^3J = 12.0 Hz, 1H, H_4), 4.41 (dd, ^3J = 12.0 Hz, 6.8 Hz, 1H, H_3), 4.03 (d, ^3J = 6.8 Hz, 1H, H_2), 3.70 (s, 3H, OMe), 1.56 (s, 3H, Me), 1.42 (s, 3H, Me).

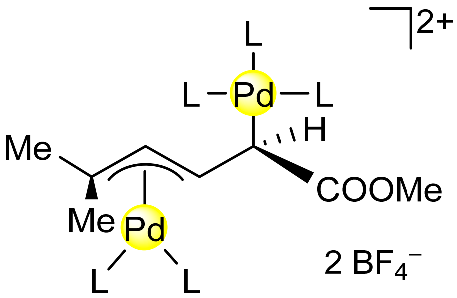

(L = CH_3CN)

3-transoid-antifacial

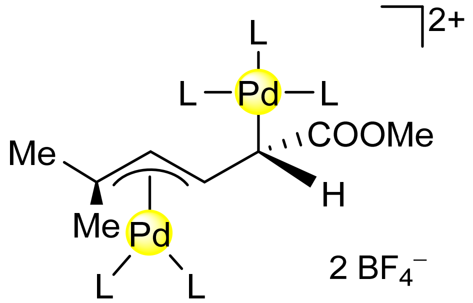

(L = CH_3CN)

3-cisoid-antifacial

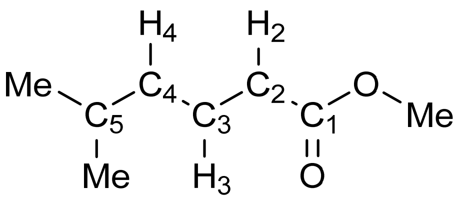

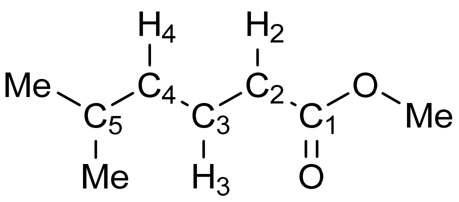

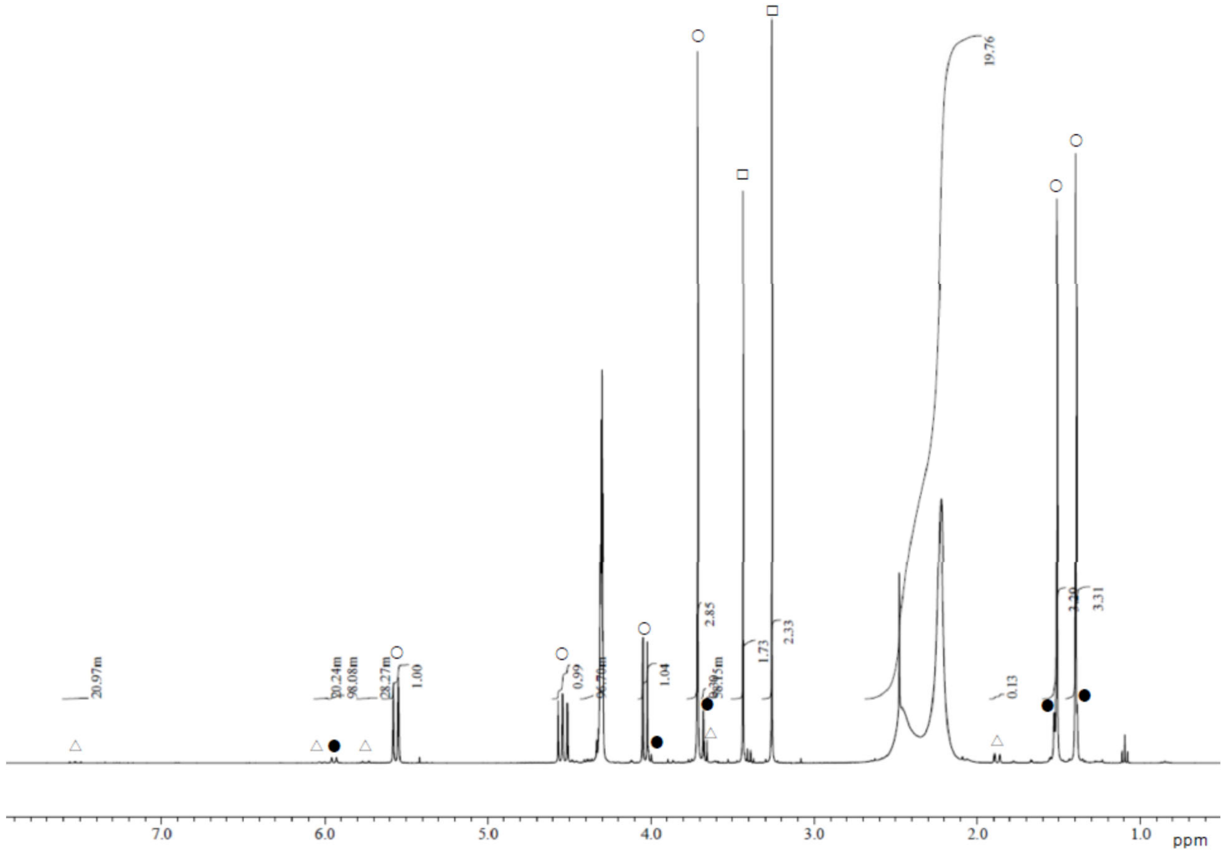

Supplementary Fig 1. ^{1}H NMR spectrum of **3-transoid-antifacial** (○) and **3-cisoid-antifacial** (●) in CD_2Cl_2. △ = methyl (*E*)-4-methylpenta-2,4-dienoate (*E*-**2**)

Preparation of *E*-dienes

Methyl (*E*)-5-methylhexa-2,4-dienoate (*E*-**2**)

To a suspension of NaH (0.65 g (60wt%), 1.62 x 10 mmol) in THF was added a THF solution of methyl 2-(dimethoxyphosphoryl)acetate (2.95 g, 1.62 x 10 mmol) at 0 °C. The reaction mixture was stirred for 30 min. 3-Methylbut-2-enal (1.35 g, 1.60 x 10 mmol) was added to the suspension, and then the mixture was stirred overnight at room temperature. The reaction mixture was quenched with NH_4Cl (aq) and extracted with diethyl ether. The combined organic layers were washed with brine and dried over $MgSO_4$. The solvent was removed *in vacuo* and the residual oil was purified by silica gel column chromatography (hexane/CH_2Cl_2) to give methyl (*E*)-5-methylhexa-2,4-dienoate (*E*-**2**) as a colorless oil (1.20 g, 8.56 mmol, 53% yield). The spectroscopic data were consistent with those reported in the literature.[5,10] ^{1}H NMR (400 MHz, $CDCl_3$, 25 °C) δ 7.56 (dd, ^3J = 15.2 Hz, and 11.6 Hz, 1H, H_3), 5.99 (d, ^3J = 11.6 Hz, 1H, H_4), 5.76 (d, ^3J = 15.2 Hz, 1H, H_2), 3.74 (s, 3H, OMe), 1.89 (s, 3H, Me), 1.87 (s, 3H, Me). HRMS-EI (m/z): $[M]^+$ calcd for $C_8H_{12}O_2$, 140.0837; found, 140.0839.

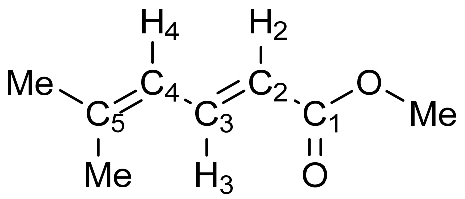

The following *E*-dienes were prepared according to the procedure for preparation of methyl (*E*)-5-methylhexa-2,4-dienoate.

Methyl (*E*)-4-cyclohexylidenebut-2-enoate (*E*-**5**)

Methyl (*E*)-4-(diethoxyphosphoryl)but-2-enoate (2.00 g, 8.47 mmol), cyclohexanone (0.910 g, 9.27 mmol) and NaH (0.339 g (60wt%), 8.47 mmol) were used. Methyl (*E*)-4-cyclohexylidenebut-2-enoate (*E*-**5**) was isolated as colorless oil (0.560 g, 3.11 mmol, 37% yield). The spectroscopic data were consistent with those reported in the literature.[11] ^{1}H NMR (400 MHz, $CDCl_3$, 25 °C) δ 7.63 (dd, ^3J = 15.2 Hz, and 11.6 Hz, 1H, H_3), 5.93 (d, ^3J = 11.6 Hz, 1H, H_4), 5.79 (d, ^3J = 15.2 Hz, 1H, H_2), 3.73 (s, 3H, OMe), 2.42-2.38 (m, 2H, Cy), 2.23-2.18 (m, 2H, Cy), 1.63-1.56 (m, 6H, Cy). HRMS-EI (m/z): $[M]^+$ calcd for $C_{11}H_{16}O_2$, 180.1150; found, 180.1151.

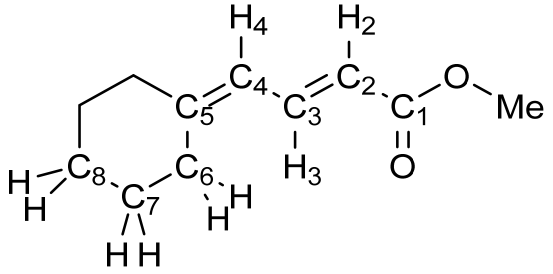

Methyl (*E*)-4-adamantan-2-ylidenebut-2-enoate (*E*-**6**)

Methyl (*E*)-4-(dimethoxyphosphoryl)but-2-enoate (0.680 g, 3.26 mmol), 2-adamantanone (0.490 g, 3.26 mmol) and NaH (0.260 g (60wt%), 6.52 mmol) were used. Methyl (*E*)-4-adamantan-2-ylidenebut-2-enoate (*E*-**6**) was isolated as colorless solid (0.262 g, 1.13 mmol, 35% yield). ^{1}H NMR (400 MHz, $CDCl_3$, 25 °C) δ 7.65 (dd, ^{3}J = 14.8 Hz, and 11.6 Hz, 1H, H_3), 5.92 (d, ^{3}J = 11.6 Hz, 1H, H_4), 5.80 (d, ^{3}J = 14.8 Hz, 1H, H_2), 3.73 (s, 3H, OMe), 3.19 (s, 1H, Ad), 2.47 (s, 1H, Ad), 1.97-1.92 (m, 6H, Ad), 1.86-1.75 (m, 6H, Ad). ^{13}C NMR ($CDCl_3$, 101 MHz, 25 °C) δ 168.2 (C_1),163.2 (C_5), 140.4 (C_3), 117.8 (C_2), 116.0 (C_4), 51.3 (OMe), 41.3 (Ad), 40.0 (Ad), 39.2 (Ad), 36.9 (Ad), 33.4 (Ad), 28.3 (Ad). HRMS-EI (m/z): $[M]^+$ calcd for $C_{15}H_{20}O_2$, 232.1463; found, 232.1457.

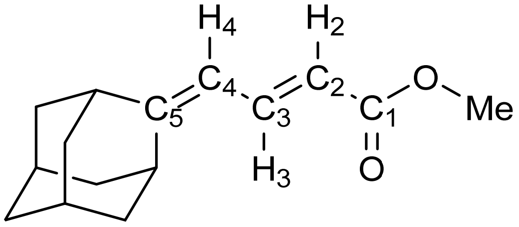

Benzyl (*E*)-5-methylhexa-2,4-dienoate (*E*-**7**)

Benzyl 2-(dimethoxyphosphoryl)acetate (0.500 g, 1.94 mmol), 3-methylbut-2-enal (0.192 g, 1.93 mmol) and NaH (0.0772 g (60wt%), 1.93 mmol) were used. Benzyl (*E*)-5-methylhexa-2,4-dienoate (*E*-**7**) was isolated as colorless solid (0.378 g, 1.74 mmol, 90% yield). The spectroscopic data were consistent with those reported in the literature.[12] ^{1}H NMR (400 MHz, $CDCl_3$, 25 °C) δ 7.61 (dd, ^{3}J = 15.2 Hz, and 11.6 Hz, 1H, H_3), 7.38-7.29 (m, 5H, Ph), 5.99 (d, ^{3}J = 11.6 Hz, 1H,

H_4), 5.81 (d, ^3J = 15.2 Hz, 1H, H_2), 5.19 (s, 2H, H_6), 1.89 (s, 3H, Me), 1.87 (s, 3H, Me). HRMS-EI (m/z): $[M]^+$ calcd for $C_{14}H_{16}O_2$, 216.1150; found, 216.1153.

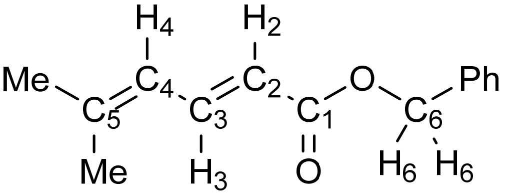

Methyl (*E*)-5,5-diphenylpenta-2,4-dienoate (*E*-**8**)

Methyl 2-(dimethoxyphosphoryl)acetate (0.480 g, 2.64 mmol), β-phenylcinnamaldehyde (0.500 g, 2.40 mmol) and NaH (0.106 g (60wt%), 2.65 mmol) were used. Methyl (*E*)-5,5-diphenylpenta-2,4-dienoate (*E*-**8**) was isolated as colorless solid (0.523 g, 1.98 mmol, 83% yield). The spectroscopic data were consistent with those reported in the literature.[13] ^{1}H NMR (400 MHz, $CDCl_3$, 25 °C) δ 7.45-7.29 (m, 9H, Ph and H_3), 7.22-7.20 (m, 2H, Ph), 6.80 (d, ^3J = 11.6 Hz, 1H, H_4), 6.06 (d, ^3J = 15.2 Hz, 1H, H_2), 3.71 (s, OMe). HRMS-EI (m/z): $[M]^+$ calcd for $C_{18}H_{16}O_2$, 264.1150; found, 264.1151.

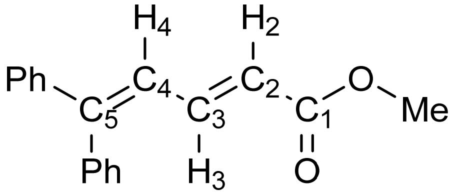

Methyl (*E*)-4-(4-methylpenta-1,3-dien-1-yl)benzoate (*E*-**9**)

Methyl 4-((dimethoxyphosphoryl)methyl)benzoate (5.64 g, 2.18 x 10 mmol), 3-methyl-2-butenal (2.02 g, 2.40 x 10 mmol) and NaH (0.873 g (60wt%), 2.18 x 10 mmol) were used. Methyl (*E*)-4-(4-methylpenta-1,3-dien-1-yl)benzoate (*E*-**9**) was isolated as white solid (1.02 g, 4.72 mmol, 22% yield). ^{1}H NMR (400 MHz, $CDCl_3$, 25 °C) δ 7.96 (d, ^3J = 8.8 Hz, 2H, H_7), 7.43 (d, ^3J = 8.8 Hz, 2H, H_6), 7.10 (dd, ^3J = 14.8 Hz, and 11.6 Hz, 1H, H_2), 6.43 (d, ^3J = 16.0 Hz, 1H, H_1), 6.02 (d, ^3J = 10.8 Hz, 1H, H_3), 3.91 (s, 3H, OMe), 1.89 (s, 3H, Me), 1.87 (s, 3H, Me). ^{13}C NMR ($CDCl_3$, 101 MHz, 25 °C) δ 167.0 (C_9), 142.7 (C_5), 138.8 (C_4), 129.9 (C_7), 128.4 (C_1), 128.1 (C_2 and C_8), 125.8

(C_6), 125.3 (C_3), 52.0 (OMe), 26.4 (Me), 18.7 (Me). HRMS-ESI (m/z): $[M+Na]^+$ calcd for $C_{14}H_{16}O_2$, 239.1043; found, 239.1088.

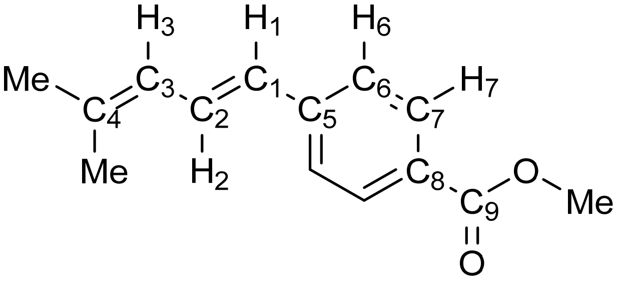

(*E*)-1-(4-methylpenta-1,3-dien-1-yl)-4-(trifluoromethyl)benzene (*E*-**10**)

dimethyl (4-(trifluoromethyl)benzyl)phosphonate (5.06 g, 1.89 x 10 mmol), 3-methyl-2-butenal (2.65 g, 2.44 x 10 mmol) and NaH (0.755 g (60wt%), 1.89 x 10 mmol) were used. (*E*)-1-(4-methylpenta-1,3-dien-1-yl)-4-(trifluoromethyl)benzene (*E*-**10**) was isolated as white solid (1.63 g, 7.20 mmol, 38% yield). ^{1}H NMR (400 MHz, $CDCl_3$, 25 °C) δ 7.53 (d, ^3J = 6.8 Hz, 2H, H_7), 7.46 (d, ^3J = 6.8 Hz, 2H, H_6), 7.07 (dd, ^3J = 15.2 Hz, and 10.4 Hz, 1H, H_2), 6.43 (d, ^3J = 15.2 Hz, 1H, H_1), 6.03 (d, ^3J = 10.4 Hz, 1H, H_3), 1.88 (s, 3H, Me), 1.87 (s, 3H, Me). ^{13}C NMR ($CDCl_3$, 101 MHz, 25 °C) δ 141.6 (C_5), 138.8 (C_4), 128.0 (C_2), 127.9 (C_1), 126.0 (C_6), 125.5 (C_7 and C_8), 125.2 (C_3), 26.3 (Me), 18.7 (Me). ^{19}F NMR (376 MHz, $CDCl_3$, 25 °C) δ -62.3. HRMS-ESI (m/z): $[M+Na+K-H]^+$ calcd for $C_{13}H_{13}F_3$, 287.0420; found, 287.0756.

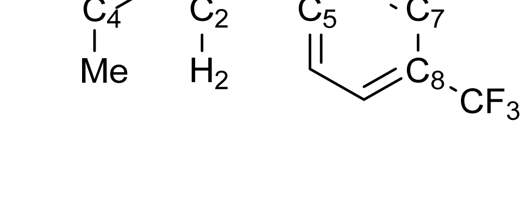

Methyl (2*E*,4*E*)-5-phenylpenta-2,4-dienoate ((2*E*,4*E*)-**11**)

Methyl 2-(dimethoxyphosphoryl)acetate (2.29 g, 1.26 x 10 mmol), cinnamaldehyde (1.53 g, 1.16 x 10 mmol) and NaH (0.51 g (60wt%), 1.28 x 10 mmol) were used. Methyl (2*E*,4*E*)-5-phenylpenta-2,4-dienoate ((2*E*,4*E*)-**11**) was isolated as colorless solid (1.52 g, 8.07 mmol, 70% yield). The spectroscopic data were consistent with those reported in the literature.[14] ^{1}H NMR (400 MHz, $CDCl_3$, 25 °C) δ 7.50-7.25 (m, 6H, Ph and H_3), 6.94-6.84 (m, 2H, H_4 and H_5), 6.00 (d, ^3J = 16.0 Hz, 1H, H_2), 3.77 (s, OMe). HRMS-ESI (m/z): $[M+Na]^+$ calcd for $C_{12}H_{12}O_2$, 211.0730; found, 211.0754.

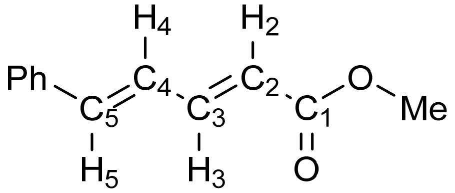

Methyl (2*E*,4*E*)-5-(4-nitrophenyl)penta-2,4-dienoate ((2*E*,4*E*)-**14**)

Methyl 2-(dimethoxyphosphoryl)acetate (1.98 g, 1.09 x 10 mmol), (*E*)-4-nitrocinnamaldehyde (1.72 g, 9.71 mmol) and NaH (0.43 g (60wt%), 1.08 x 10 mmol) were used. Methyl (2*E*,4*E*)-5-(4-nitrophenyl)penta-2,4-dienoate ((2*E*,4*E*)-**14**) was isolated as yellow solid (1.09 g, 4.67 mmol, 48% yield). ^{1}H NMR (400 MHz, $CDCl_3$, 25 °C) δ 8.21 (d, ^3J = 10.8 Hz, 1H, H_8), 7.60 (d, ^3J = 10.8 Hz, 2H, H_7), 7.45 (dd, ^3J = 15.2 Hz, and 10.2 Hz, 1H, H_3), 7.00 (dd, ^3J = 16.0 Hz, and 10.2 Hz, 1H, H_4), 6.93 (d, ^3J = 16.0 Hz, 1H, H_5), 6.11 (d ^3J = 15.2 Hz, 1H, H_2), 3.79 (s, 3H, H_{10}). ^{13}C NMR (101 MHz, $CDCl_3$, 25 °C) δ 167.0 (C_1), 147.6 (C_9), 143.4 (C_3), 142.2 (C_6), 137.4 (C_5), 130.3 (C_4), 127.6 (C_7), 124.2 (C_8), 123.6 (C_2), 51.8 (C_{10}). HRMS-ESI (m/z): $[M+Na]^+$ calcd for $C_{12}H_{11}NO_4$, 256.0580; found, 256.0690.

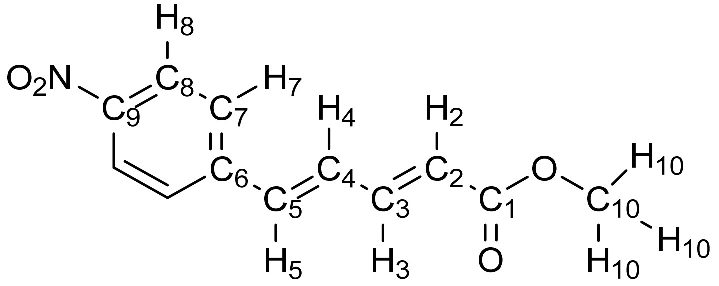

Methyl (2*E*,4*E*)-5-(4-methoxyphenyl)penta-2,4-dienoate ((2*E*,4*E*)-**15**)

Methyl 2-(dimethoxyphosphoryl)acetate (1.50 g, 8.24 mmol), (*E*)-4-methoxycinnamaldehyde (1.31 g, 8.08 mmol) and NaH (0.34 g (60wt%), 8.50 mmol) were used. Methyl (2*E*,4*E*)-5-(4-methoxyphenyl)penta-2,4-dienoate ((2*E*,4*E*)-**15**) was isolated as colorless solid (1.43 g, 6.55 mmol, 81% yield). The spectroscopic data were consistent with those reported in the literature.[15] ^{1}H NMR (400 MHz, $CDCl_3$, 25 °C) δ 7.44 (dd, ^{3}J = 15.2 Hz, and 10.8 Hz, 1H, H_3), 7.41 (d, ^{3}J = 9.2 Hz, 2H, H_7), 6.88 (d, ^{3}J = 9.2 Hz, 2H, H_8), 6.85 (d, ^{3}J = 15.2 Hz, 1H, H_5), 6.75 (dd, ^{3}J = 15.2 Hz, and 10.8 Hz, 1H, H_4), 5.94 (d ^{3}J = 15.2 Hz, 1H, H_2), 3.83 (s, 3H, H_{11}), 3.76 (s, 3H, H_{10}). ^{13}C NMR (101 MHz, $CDCl_3$, 25 °C) δ 167.7 (C_1), 160.4 (C_9), 145.3 (C_3), 140.3 (C_5), 128.8 (C_6), 128.6 (C_7), 124.1 (C_4), 119.5 (C_2), 114.2 (C_8), 55.3 (C_{11}), 51.5 (C_{10}). HRMS-ESI (m/z): $[M+Na]^+$ calcd for $C_{13}H_{14}O_3$, 241.0835; found, 241.0951.

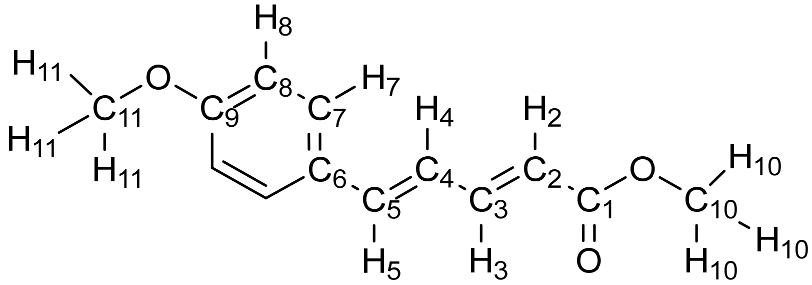

Methyl (2*E*,4*E*)-6,6-dimethylhepta-2,4-dienoate ((2*E*,4*E*)-**16**)

Methyl 4-(dimethoxyphosphoryl)but-2-enoate (1.65 g, 7.93 mmol), pivalaldehyde (0.70 g, 7.9 mmol) and lithium bis(trimethylsilyl)amide (1.34 g, 7.93 mmol) were used. Methyl (2*E*,4*E*)-6,6-dimethylhepta-2,4-dienoate ((2*E*,4*E*)-**16**) was isolated as colorless oil (0.97 g, 5.77 mmol, 73% yield). ^{1}H NMR (400 MHz, $CDCl_3$, 25 °C) δ 7.27 (dd, ^{3}J = 16.0 Hz, and 9.2 Hz, 1H, H_3), 6.16-6.06 (m, 2H, H_4 and H_5), 5.82 (d, ^{3}J = 16.0 Hz, 1H, H_2), 3.74 (s, 3H, H_8), 1.06 (s, 9H, H_7). ^{13}C NMR (101 MHz) δ 167.7 (C_1), 155.2 (C_5), 145.9 (C_3), 123.3 (C_4), 118.9 (C_2), 51.4 (C_8), 33.9 (C_6), 29.0 (C_7). HRMS-ESI (m/z): $[M+Na]^+$ calcd for $C_{10}H_{16}O_2$, 191.1043; found, 191.0908.

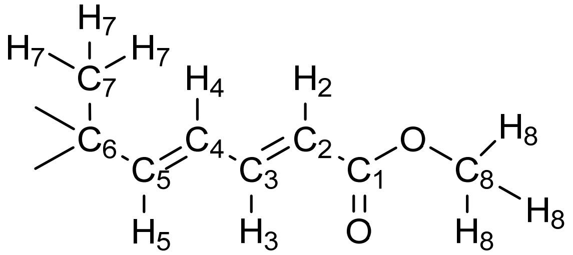
H7
H7
H7
C7
H4
H2
C6
C4
C2
O
H8
C5
C3
C1
C8
H8
H5
H3
O
H8

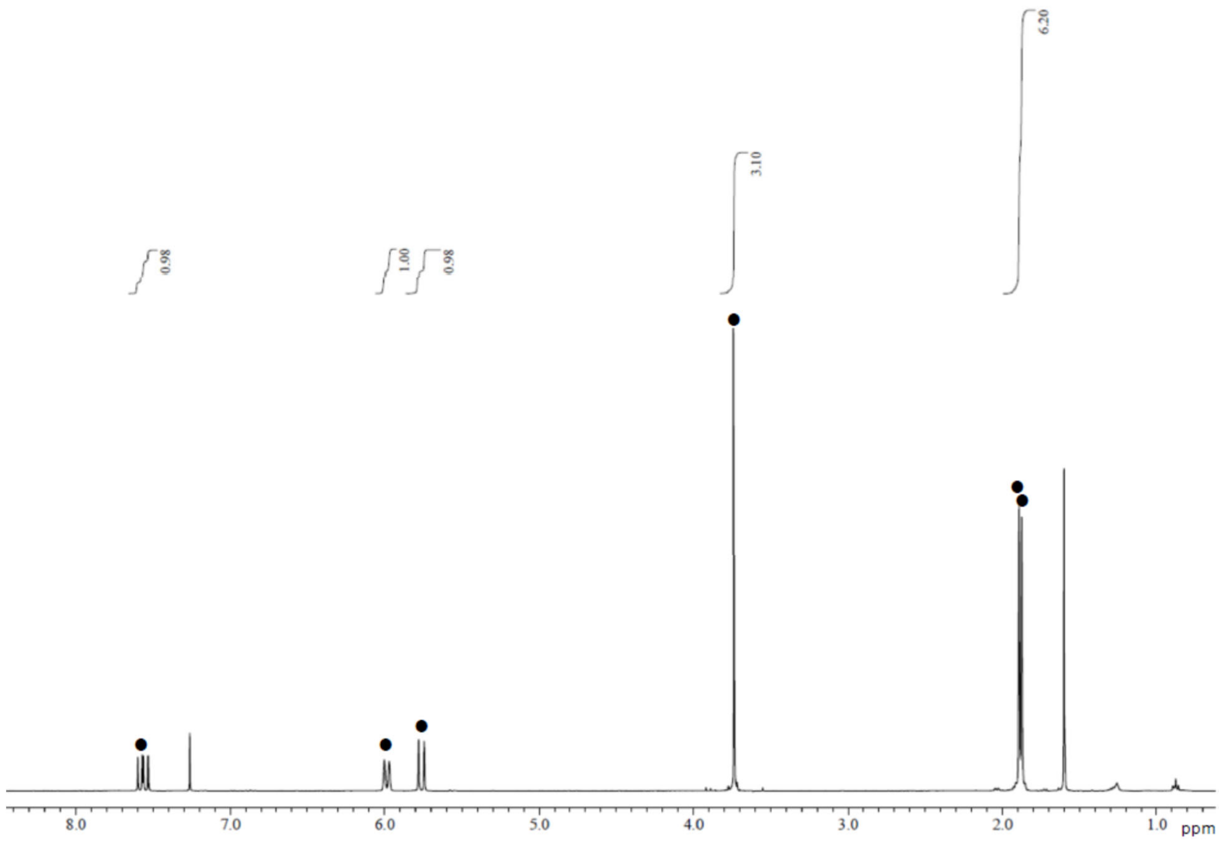

Supplementary Fig 2. ^{1}H NMR spectrum of methyl (*E*)-5-methylhexa-2,4-dienoate (*E*-**2**) (●) in $CDCl_3$.

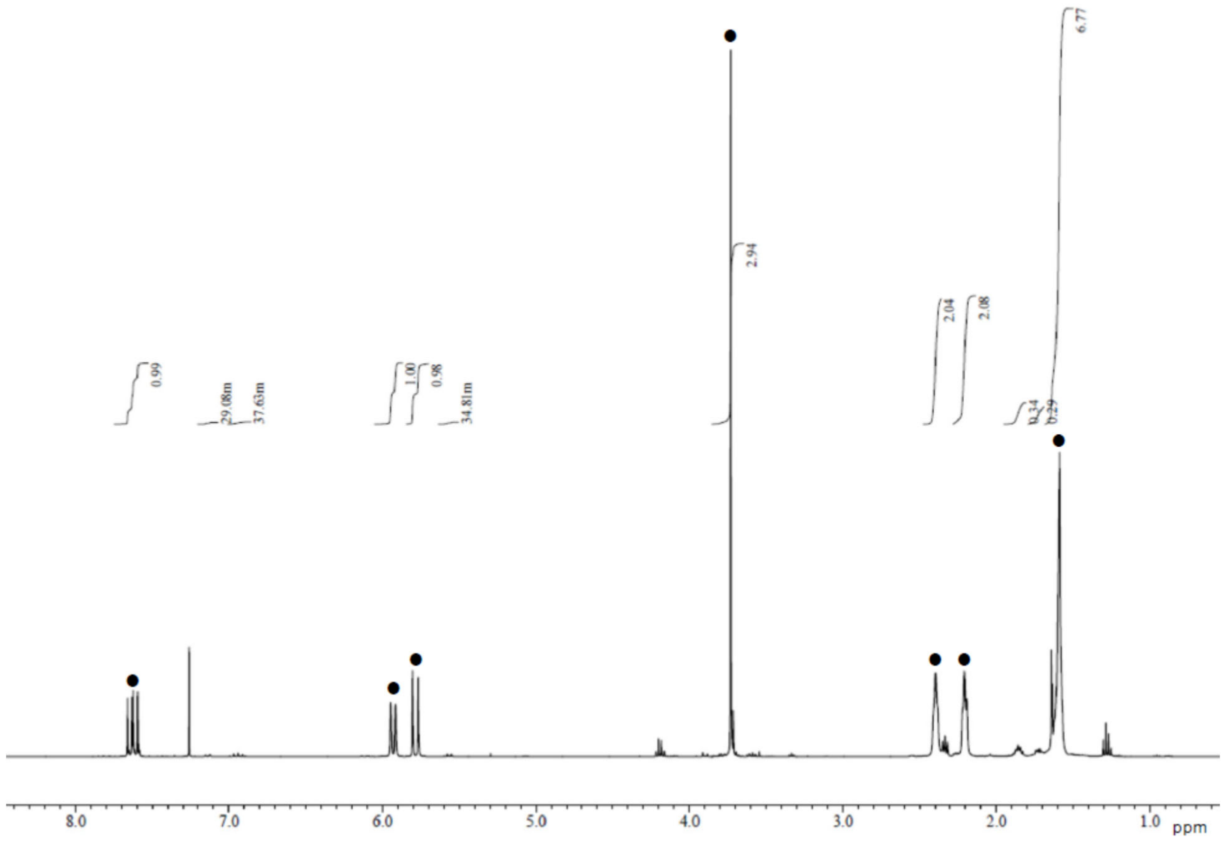

Supplementary Fig 3. ^{1}H NMR spectrum of methyl (*E*)-4-cyclohexylidenebut-2-enoate (*E*-**5**) (●) in $CDCl_3$.

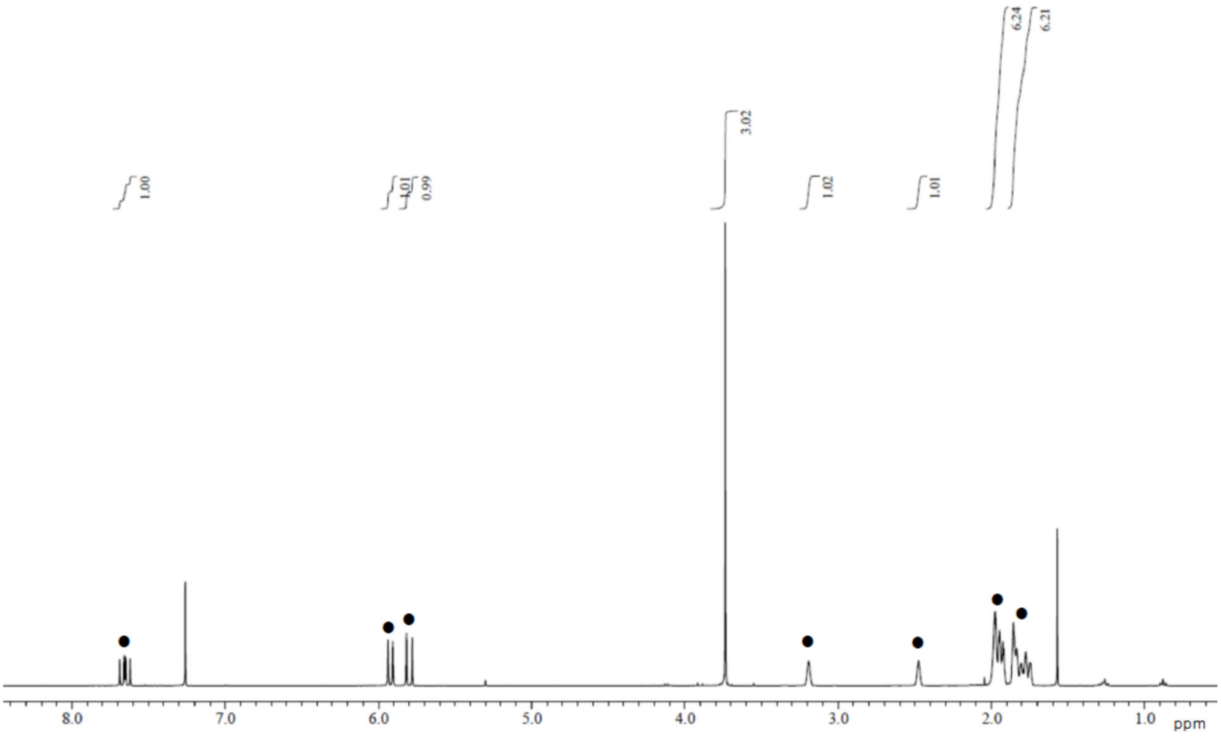

Supplementary Fig 4. ^{1}H NMR spectrum of methyl (*E*)-4-adamantan-2-ylidenebut-2-enoate (*E*-**6**) (●) in $CDCl_3$.

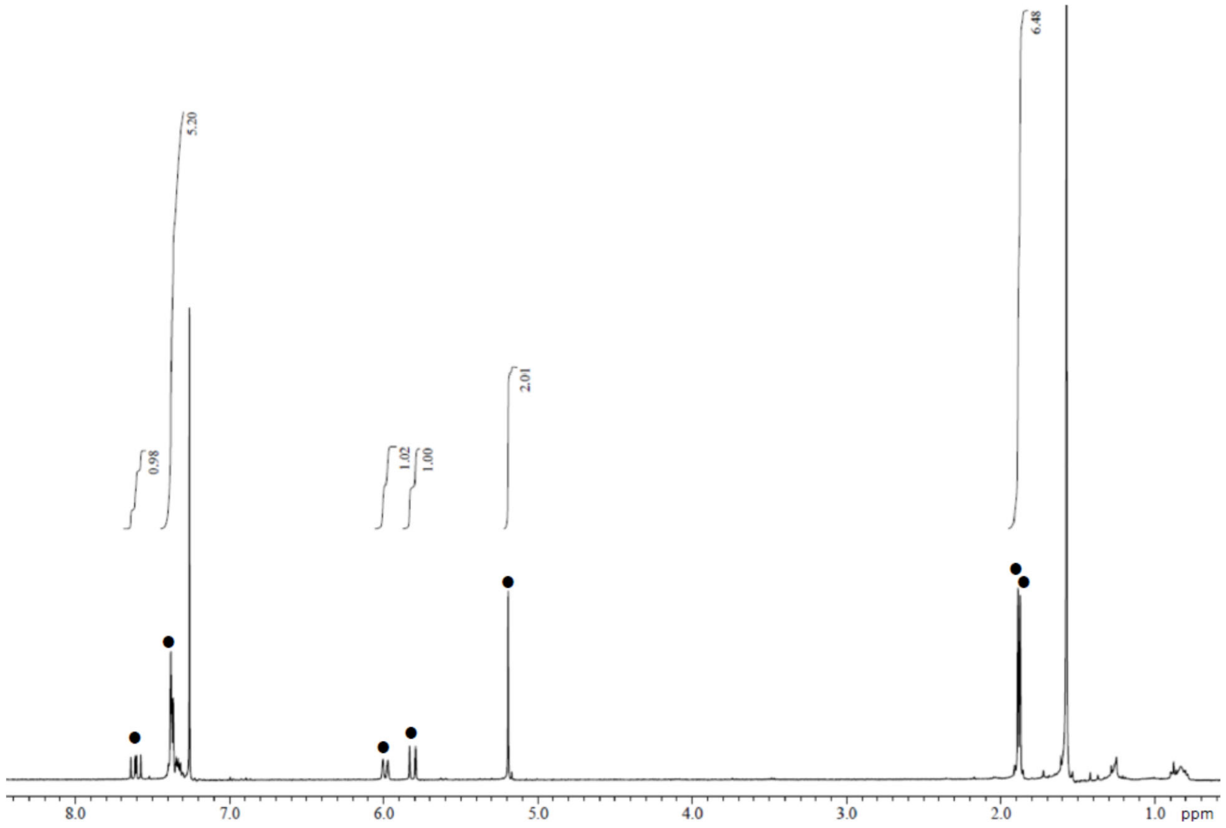

Supplementary Fig 5. ^{1}H NMR spectrum of benzyl (*E*)-5-methylhexa-2,4-dienoate (*E*-**7**) (●) in $CDCl_3$.

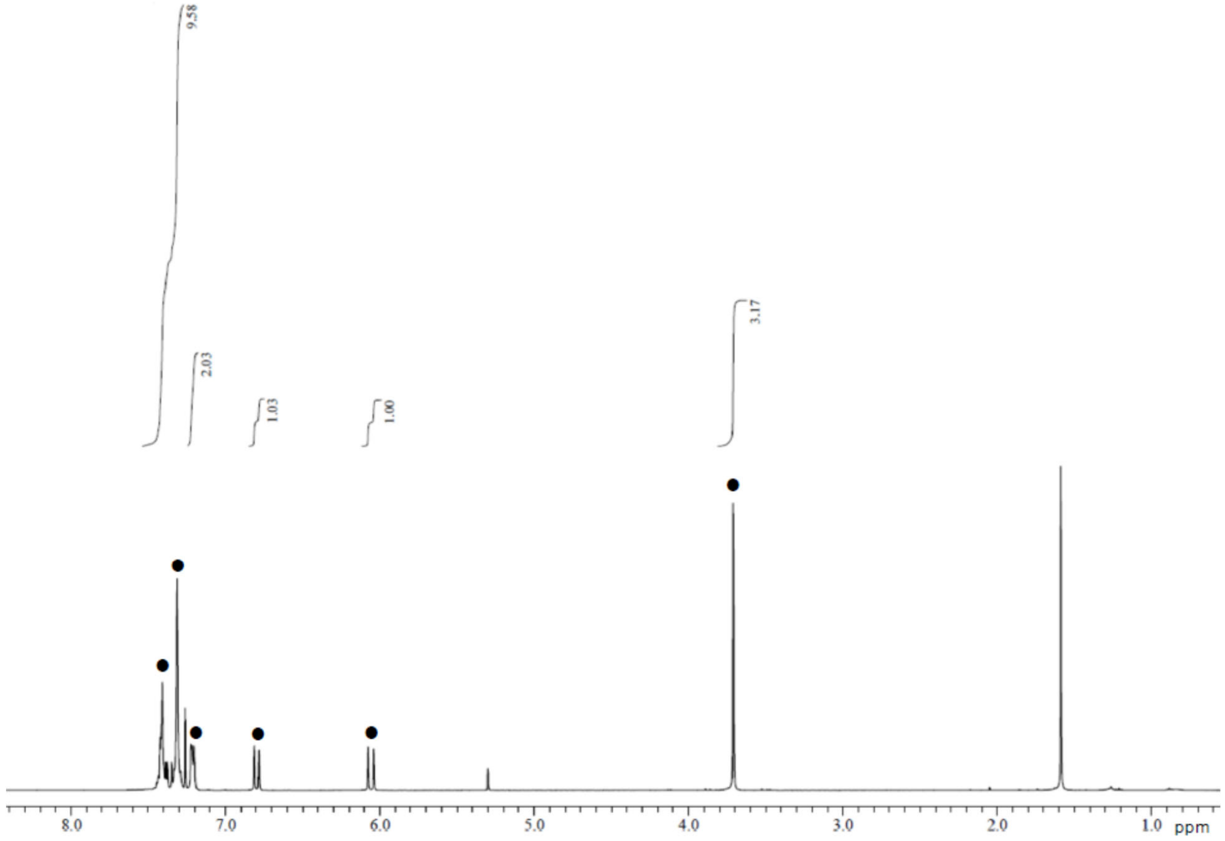

Supplementary Fig 6. ^{1}H NMR spectrum of methyl (*E*)-5,5-diphenylpenta-2,4-dienoate (*E*-**8**) (●) in $CDCl_3$.

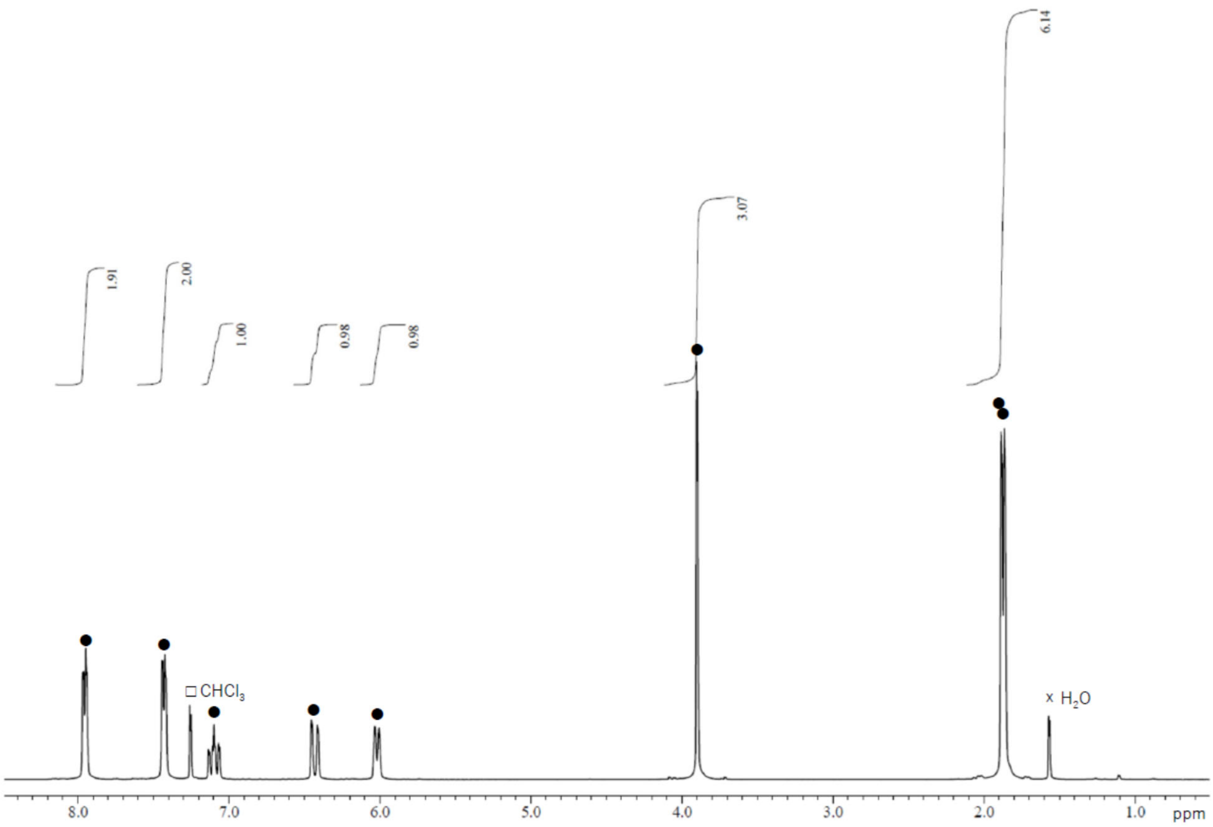

Supplementary Fig 7. ^{1}H NMR spectrum of methyl (*E*)-4-(4-methylpenta-1,3-dien-1-yl)benzoate (***E*-9**) (●) in $CDCl_3$.

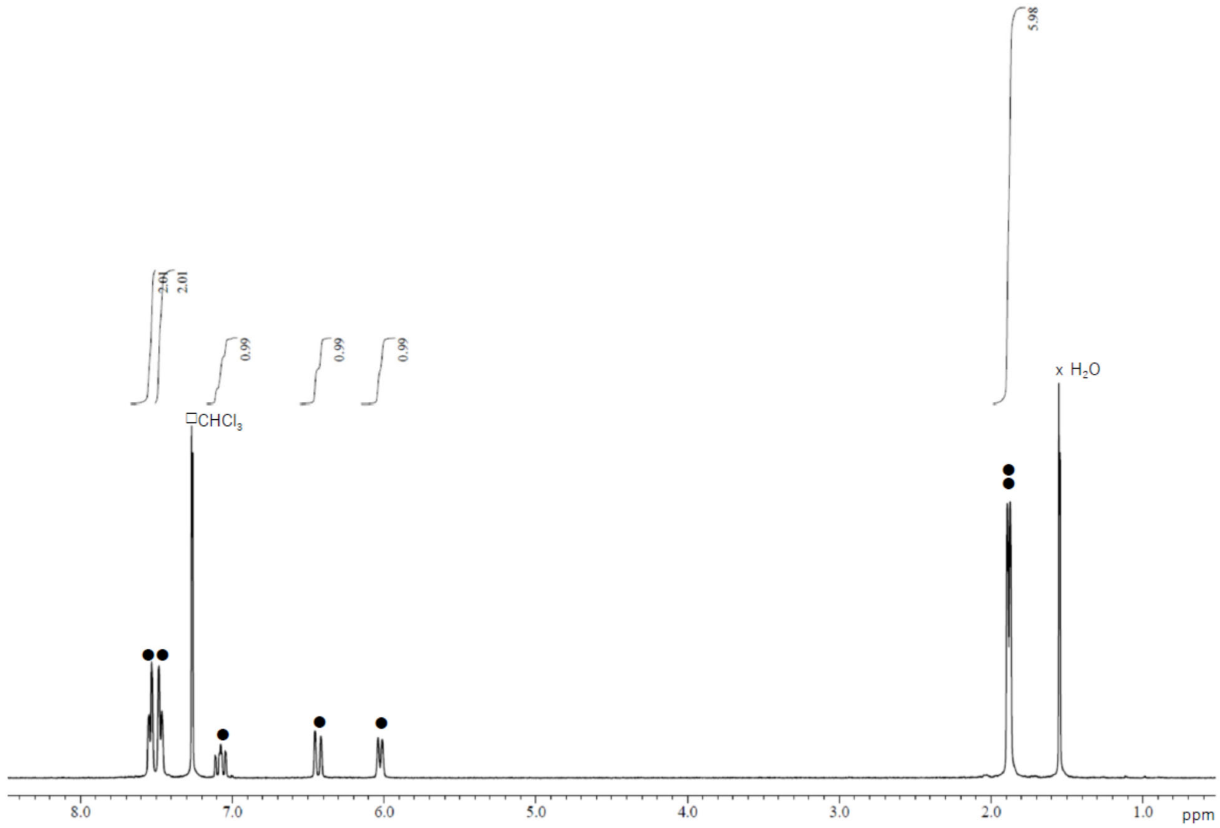

Supplementary Fig 8. ^{1}H NMR spectrum of (*E*)-1-(4-methylpenta-1,3-dien-1-yl)-4-(trifluoromethyl)benzene (***E*-10**) (●) in $CDCl_3$.

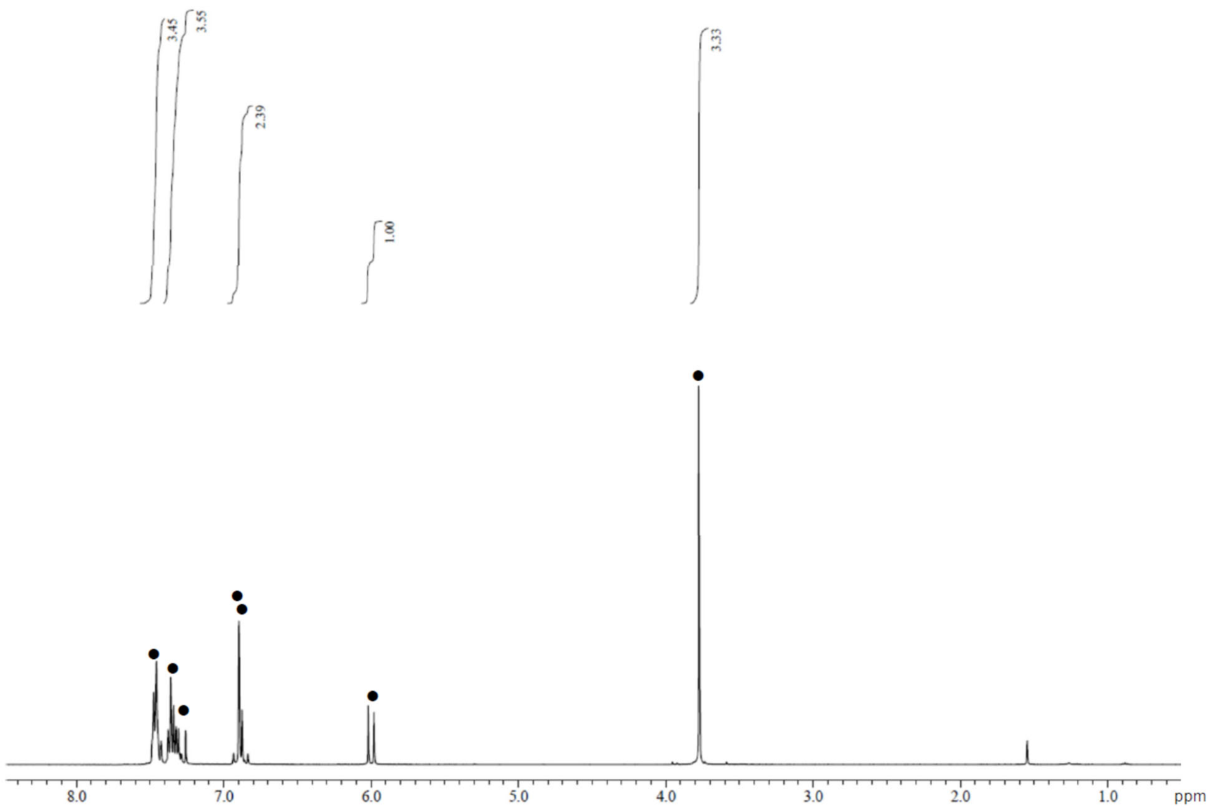

Supplementary Fig 9. ^{1}H NMR spectrum of methyl (2*E*,4*E*)-5-phenylpenta-2,4-dienoate ((2*E*,4*E*)-**11**) (●) in $CDCl_3$.

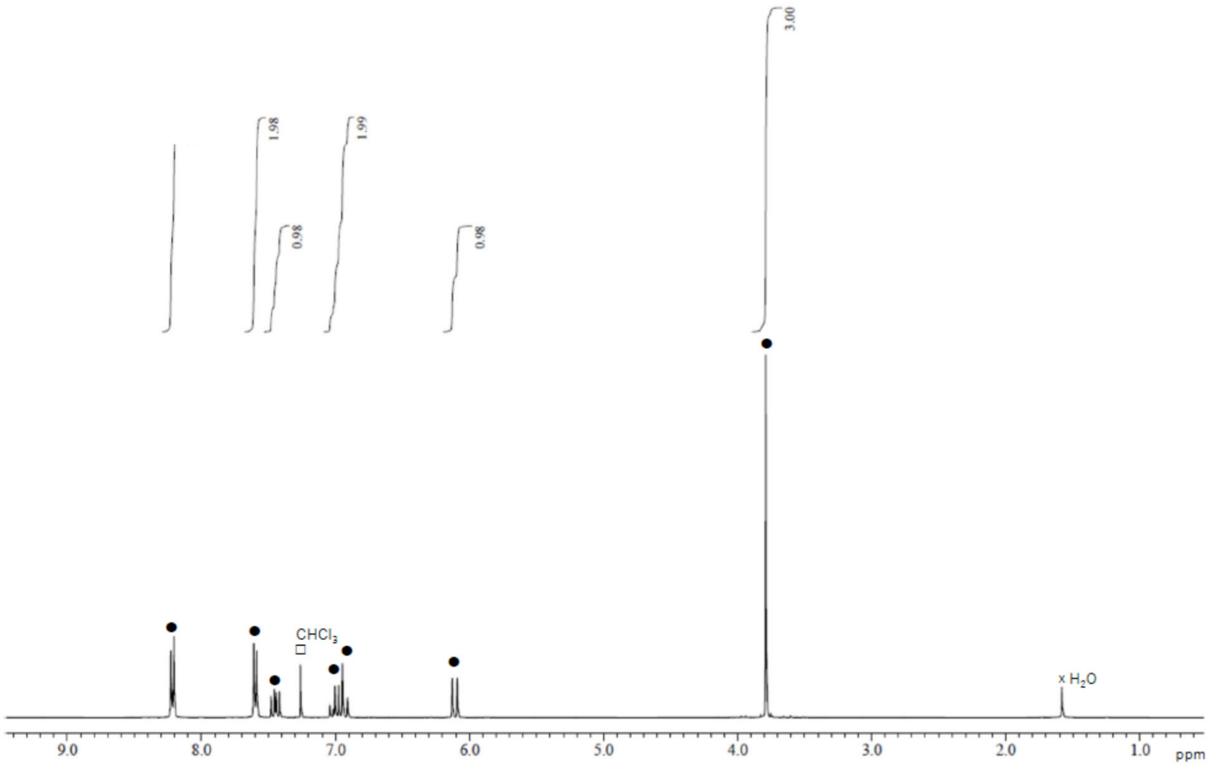

Supplementary Fig 10. ^{1}H NMR spectrum of methyl (2*E*,4*E*)-5-(4-nitrophenyl)penta-2,4-dienoate ((2*E*,4*E*)-**14**)) (●) in $CDCl_3$.

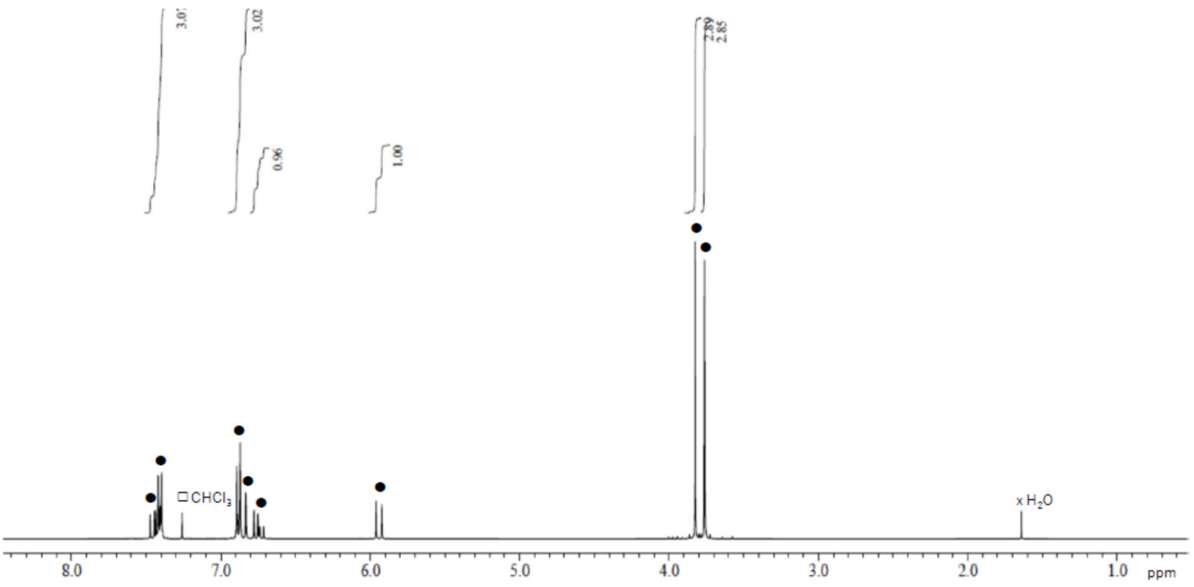

Supplementary Fig 11. ^{1}H NMR spectrum of methyl (2*E*,4*E*)-5-(4-methoxyphenyl)penta-2,4-dienoate ((2*E*,4*E*)-**15**) (●) in $CDCl_3$.

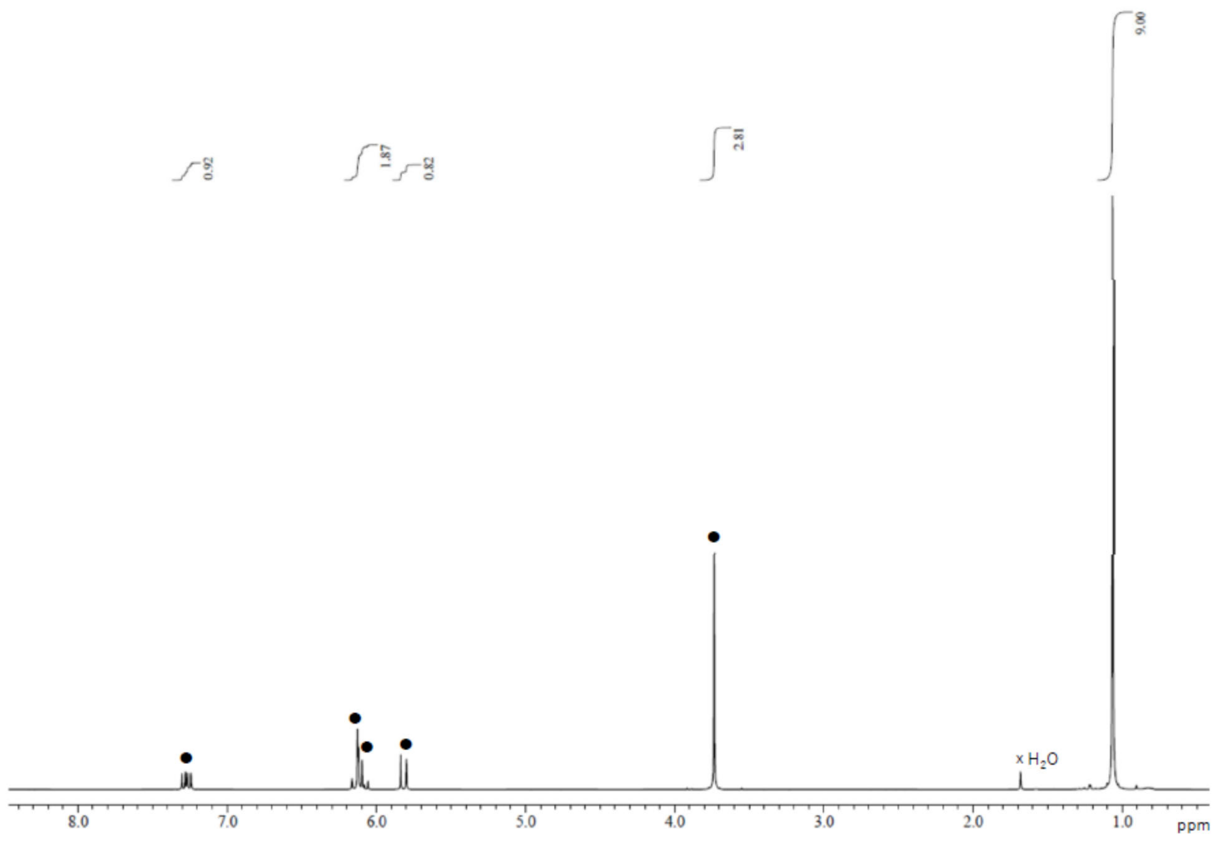

Supplementary Fig 12. ^{1}H NMR spectrum of methyl (2*E*,4*E*)-6,6-dimethylhepta-2,4-dienoate ((2*E*,4*E*)-**16**) (●) in $CDCl_3$.

General procedure for elimination of diene **2** from complex **3**

To a solution of $[Pd_2(CH_3CN)_6][BF_4]_2$ (**1**, 10.0 mg, 1.6 x 10^{-2} mmol) in CD_3NO_2 was added the methyl (*E*)-5-methylhexa-2,4-dienoate (*E*-**2**, 2.2 mg, 1.6 x 10^{-2} mmol) at room temperature. 2 equiv. of a ligand (3.2 x 10^{-2} mmol) was added into the reaction mixture. The E/Z ratio of the **2** through elimination from complex **3** was determined by ^{1}H NMR analysis (Supplementary Table 1).

Supplementary Table 1. The Ligand Survey for Stereo-retentive Elimination of mmd.

ligand	yield [%]	*E* : *Z*
PPh_3	67	>99 : <1
$P(OPh)_3$	83	40 : 60
dppm	99	80 : 20
PPh_4I	99	49 : 51
PPh_4Br	20	>99 : <1
PPh_4Cl	5	>99 : <1
1,6-diphenylhexatriene	70	>99 : <1
1,5-cyclooctadiene	14	50 : 50
norbornadiene	91	51 : 49
1,3,5-cycloheptatriene	99	18 : 82
1,3,5,7-cyclooctatetraene	82	9 : 91
1,3,5,7-cyclooctatetraene[a]	80	4 : 96

[a] at -30 ºC

E to *Z* isomerization of dienes through *syn*-elimination of transoid-antifacial-Pd_2 complexes by using COT

Methyl (*Z*)-5-methylhexa-2,4-dienoate (*Z*-**2**)

To a solution of $[Pd_2(CH_3CN)_6][BF_4]_2$ (**1**, 126.5 mg, 2.00 x 10^{-1} mmol) in CH_3NO_2 was added the methyl (*E*)-5-methylhexa-2,4-dienoate (*E*-**2**, 28.0 mg, 2.00 x 10^{-1} mmol) and stirred at room temperature for 30 min. The reaction mixture was cooled to -30 °C and COT (41.7 mg, 4.00 x 10^{-1} mmol, 2 equiv.) was added. After stirring 15 min, the mixture was diluted with diethyl ether, and filtered through a silica gel pad. The solvent was removed *in vacuo* and the residue was purified

by a silica gel column chromatography to give methyl (*Z*)-5-methylhexa-2,4-dienoate (*Z*-**2**) as colorless oil (22.4 mg, 1.60 x 10^{-1} mmol, 80% yield, *E* : *Z* = <1 : >99). ^{1}H NMR (400 MHz, $CDCl_3$, 25 °C) δ 7.19-7.15 (m, 1H), 6.86 (dd, ^{3}J = 11.6, and 11.6 Hz, 1H), 5.56 (d, ^{3}J = 11.6 Hz, 1H), 3.71 (s, OMe, 3H), 1.91 (s, 3H, Me), 1.85 (s, 3H, Me). ^{13}C NMR ($CDCl_3$, 101 MHz, 25 °C) δ 167.3, 146.9, 140.7, 121.9, 114.1, 51.0, 26.8, 18.2. HRMS-EI (m/z): $[M]^+$ calcd for $C_8H_{12}O_2$, 140.0837; found, 140.0836.

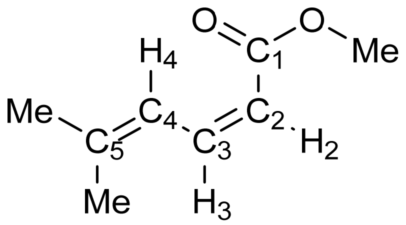

The following *Z*-dienes were prepared according to the procedure for *E* to *Z* isomerization of methyl (*E*)-5-methylhexa-2,4-dienoate.

Methyl (*Z*)-4-cyclohexylidenebut-2-enoate (*Z*-**5**)

$[Pd_2(CH_3CN)_6][BF_4]_2$ (**1**, 126.5 mg, 2.00 x 10^{-1} mmol) and methyl (*E*)-4-cyclohexylidenebut-2-enoate (*E*-**5**, 36.0 mg, 2.00 x 10^{-1} mmol) were used. Methyl (*Z*)-4-cyclohexylidenebut-2-enoate (*Z*-**5**) was obtained as Colorless oil (25.6 mg, 0.14 mmol, 71% yield, *E* : *Z* = 3 : 97). ^{1}H NMR (400 MHz, $CDCl_3$, 25 °C) δ 7.13 (d, ^{3}J = 11.6 Hz, 1H, H_4), 6.93 (dd, ^{3}J = 11.6 Hz, and 11.6 Hz, 1H, H_3), 5.56 (d, ^{3}J = 11.6 Hz, 1H, H_2), 3.71 (s, 3H, OMe), 2.38-2.32 (m, 2H, Cy), 2.28-2.24 (m, 2H, Cy), 1.64-1.53 (m, 6H, Cy). ^{13}C NMR ($CDCl_3$, 101 MHz, 25 °C) δ 167.3 (C_1), 154.9 (C_5), 140.0 (C_3), 118.6 (C_4), 114.4 (C_2), 50.9 (CO_2<u>Me</u>), 38.0 (Cy), 29.0 (Cy), 28.5 (Cy), 27.9 (Cy), 26.6 (Cy). HRMS-EI (m/z): $[M]^+$ calcd for $C_{11}H_{16}O_2$, 180.1150; found, 180.1150.

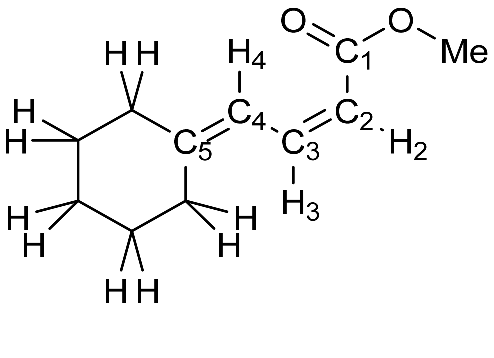

Methyl (*Z*)- 4-adamantan-2-ylidenebut-2-enoate (*Z*-**6**)

$[Pd_2(CH_3CN)_6][BF_4]_2$ (**1**, 200.0 mg, 3.16 x 10^{-1} mmol) and methyl (*E*)-4-adamantan-2-ylidenebut-2-ylidenebut-2-enoate (*E*-**6**, 73.4 mg, 3.16 x 10^{-1} mmol) were used. Methyl (*Z*)-4-adamantan-2-ylidenebut-2-enoate (*Z*-**6**) was obtained as colorless solid (59.3 mg, 2.55 x 10^{-1} mmol, 81% yield, *E* : *Z* = <1 : >99). ^{1}H NMR (400 MHz, $CDCl_3$, 25 °C) δ 7.13 (d, ^3J = 12.0 Hz, 1H, H_4), 6.95 (dd, ^3J = 12.0 Hz, and 11.2 Hz, 1H, H_3), 5.54 (d, ^3J = 11.2 Hz, 1H, H_2), 3.70 (s, 3H, OMe), 3.13 (brs, 1H, Ad), 2.55 (brs, 1H, Ad), 1.98-1.88 (m, 6H, Ad), 1.87-1.80 (m, 4H, Ad), 1.77-1.70 (m, 2H, Ad). ^{13}C NMR ($CDCl_3$, 101 MHz, 25 °C) δ 167.5 (C_1), 163.6 (C_5), 139.7 (C_3), 114.3 (C_4), 113.9 (C_2), 50.9 (CO_2Me), 41.5 (Ad), 39.8 (Ad), 39.2 (Ad), 36.9 (Ad), 32.6 (Ad), 28.3 (Ad). HRMS-EI (m/z): $[M]^+$ calcd for $C_{15}H_{20}O_2$, 232.1463; found, 232.1462.

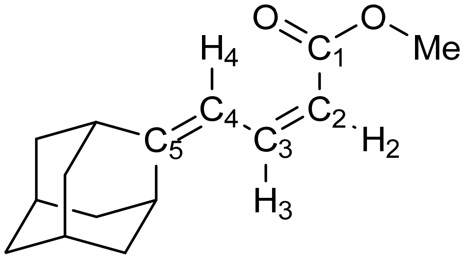

Benzyl (*Z*)-5-methylhexa-2,4-dienoate (*Z*-**7**)

$[Pd_2(CH_3CN)_6][BF_4]_2$ (**1**, 126.5 mg, 2.00 x 10^{-1} mmol) and benzyl (*E*)-5-methylhexa-2,4-dienoate (*E*-**7**, 43.3 mg, 2.00 x 10^{-1} mmol) were used. Benzyl (*Z*)-5-methylhexa-2,4-dienoate (*Z*-**7**) was obtained as colorless solid (36.0 mg, 1.66 x 10^{-1} mmol, 83% yield, *E* : *Z* = <1 : >99). ^{1}H NMR (400 MHz, $CDCl_3$, 25 °C) δ 7.41-7.29 (m, 5H, Ph), 7.23 (m, 1H, H_4), 6.90 (dd, ^3J = 11.6, 11.6 Hz, 1H, H_3), 5.62 (d, ^3J = 11.6 Hz, 1H, H_2), 5.18 (s, 2H, H_6), 1.91 (s, 3H, Me), 1.86 (s, 3H, Me). ^{13}C NMR (101 MHz) δ166.6 (C_1),147.1 (C_5), 141.1 (C_3), 136.3 (Ph), 128.5 (Ph), 128.1 (Ph), 128.0 (Ph), 122.0 (C_4), 114.2 (C_2), 65.6 (C_6), 26.9 (Me), 18.2 (Me). HRMS-EI (m/z): $[M]^+$ calcd for $C_{14}H_{16}O_2$, 216.1150; found, 216.1155.

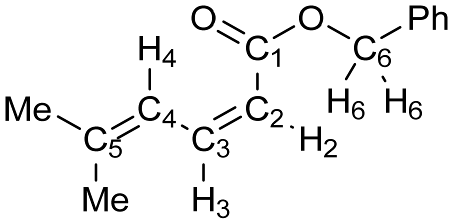

Methyl (*Z*)-4-(4-methylpenta-1,3-dien-1-yl)benzoate (*Z*-**9**)

$[Pd_2(CH_3CN)_6][BF_4]_2$ (**1**, 146.3 mg, 2.31 x 10^{-1} mmol) and methyl (*E*)-4-(4-methylpenta-1,3-dien-1-yl)benzoate (*E*-**9**, 50.0 mg, 2.31 x 10^{-1} mmol) were used. Methyl (*Z*)-4-(4-methylpenta-1,3-dien-1-yl)benzoate (*Z*-**9**) was obtained as a mixture of *E*/*Z* isomers (10.1 mg, 4.67 x 10^{-2} mmol, 20% yield, *E* : *Z* = 51 : 49). ^{1}H NMR (400 MHz, $CDCl_3$, 25 °C) δ 7.99 (d, ^3J = 8.0 Hz, 2H, H_7), 7.39 (d, ^3J = 8.0 Hz, 2H, H_6), 6.52 (dd, ^3J = 11.6 Hz, and 10.6 Hz, 1H, H_2), 6.35-6.30 (m, 2H, H_1 and H_3), 3.92 (s, 3H, OMe), 1.85 (s, 3H, Me), 1.84 (s, 3H, Me). ^{13}C NMR ($CDCl_3$, 101 MHz, 25 °C) δ 166.9 (C_9), 145.0 (C_5 or C_8), 142.7 (C_4), 140.2 (C_5 or C_8), 129.4 (C_7), 128.9 (C_6), 128.4 (C_2), 125.9 (C_3), 121.1 (C_1), 52.0 (OMe), 26.5 (Me), 18.4 (Me). HRMS-ESI (m/z): $[M+Na]^+$ calcd for $C_{14}H_{16}O_2$, 239.1043; found, 239.1091.

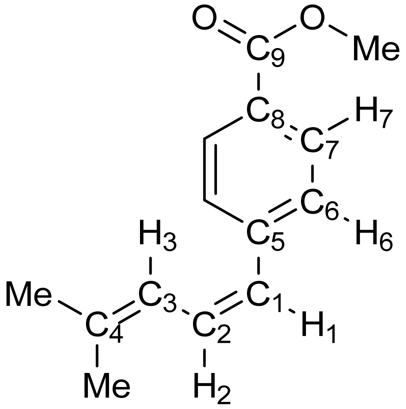

(*Z*)-1-(4-methylpenta-1,3-dien-1-yl)-4-(trifluoromethyl)benzene (*Z*-**10**)

$[Pd_2(CH_3CN)_6][BF_4]_2$ (**1**, 139.8 mg, 2.21 x 10^{-1} mmol) and (*E*)-1-(4-methylpenta-1,3-dien-1-yl)-4-(trifluoromethyl)benzene (*E*-**10**, 50.0 mg, 2.21 x 10^{-1} mmol) were used. (*Z*)-1-(4-Methylpenta-1,3-dien-1-yl)-4-(trifluoromethyl)benzene (*Z*-**10**) was obtained as a mixture of *E*/*Z* isomers (9.7 mg, 4.3 x 10^{-2} mmol, 19% yield, *E* : *Z* = 33 : 67). ^{1}H NMR (400 MHz, $CDCl_3$, 25 °C) δ 7.57 (d, ^3J = 7.6 Hz, 2H, H_7), 7.42 (d, ^3J = 7.6 Hz, 2H, H_6), 6.52 (dd, ^3J = 11.6 Hz, and 11.6 Hz, 1H, H_2), 6.31 (m, 1H, H_1), 1.85 (s, 3H, Me), 1.84 (s, 3H, Me). ^{13}C NMR ($CDCl_3$, 101 MHz, 25 °C) δ 141.6 (C_5), 140.2 (C_4), 129.1 (C_6), 128.4 (C_2), 125.4 (C_1), 125.0 (C_7), 120.9 (C_3), 26.4 (Me), 18.4 (Me). ^{19}F

NMR (376 MHz, $CDCl_3$, 25 °C) δ -62.3. HRMS-ESI (m/z): $[M+Na+K-H]^+$ calcd for $C_{13}H_{13}F_3$, 287.0420; found, 287.0752.

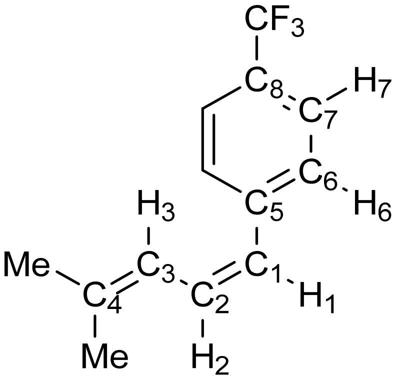

E to *Z* Isomerization of methyl (*E*)-5,5-diphenylpenta-2,4-dienoate by using PPh_4I

To a solution of $[Pd_2(CH_3CN)_6][BF_4]_2$ (**1**, 126.4 mg, 2.00 x 10^{-1} mmol) in CH_3NO_2 was added methyl (*E*)-5,5-diphenylpenta-2,4-dienoate (*E*-**8**, 52.9 mg, 2.00 x 10^{-1} mmol) and the reaction mixture was stirred for 30 min at room temperature. The reaction mixture was cooled to -30 °C, and then a nitromethane solution of PPh_4I (186,5 mg, 0.4 mmol) was added into the mixture. The resulting mixture was diluted with diethyl ether and filtered through a silica gel pad. The solvent was removed *in vacuo* and the residue was purified by a silica gel column chromatography to give (*Z*)-5,5-diphenylpenta-2,4-dienoate (*Z*-**8**, 39.0 mg, 1.47 x 10^{-1} mmol, 74 % yield, $E : Z = <1 : >99$) as a colorless solid. ^{1}H NMR (400 MHz, $CDCl_3$, 25 °C) δ 8.12 (d, $^3J = 11.6$ Hz, 1H, H_4), 7.45-7.29 (m, 8H, Ph),7.24-7.20 (m, 2H, Ph), 6.70 (dd, $^3J = 11.6$, and 10.8 Hz, 1H, H_3), 5.69 (d, $^3J = 10.8$ Hz, 1H, H_2), 3.74 (s, 3H, OMe). ^{13}C NMR (101 MHz) δ 167.1 (C_1),151.2 (C_5), 142.2 (C_3), 141.3 (Ph), 138.5 (Ph), 130.5(Ph), 128.6 (Ph), 128.3 (Ph), 128.2 (Ph), 128.2 (Ph), 128.1 (Ph), 123.2 (C_4), 117.3 (C_2), 51.4 (OMe). HRMS-EI (m/z): $[M]^+$ calcd for $C_{18}H_{16}O_2$, 264.1150; found, 264.1153.

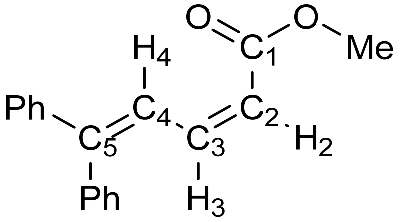

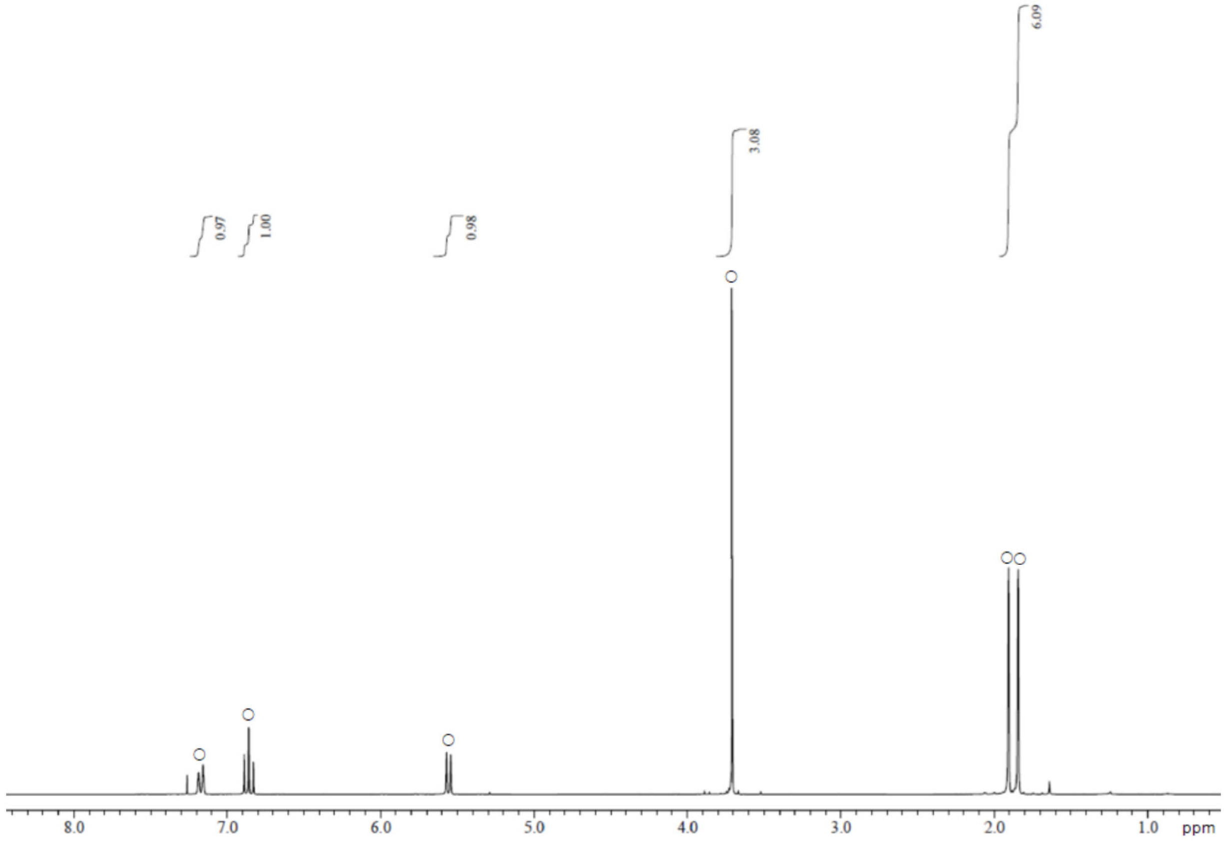

Supplementary Fig 13. ^{1}H NMR spectrum of methyl (*Z*)-5-methylhexa-2,4-dienoate (*Z*-**2**) (○) in $CDCl_3$.

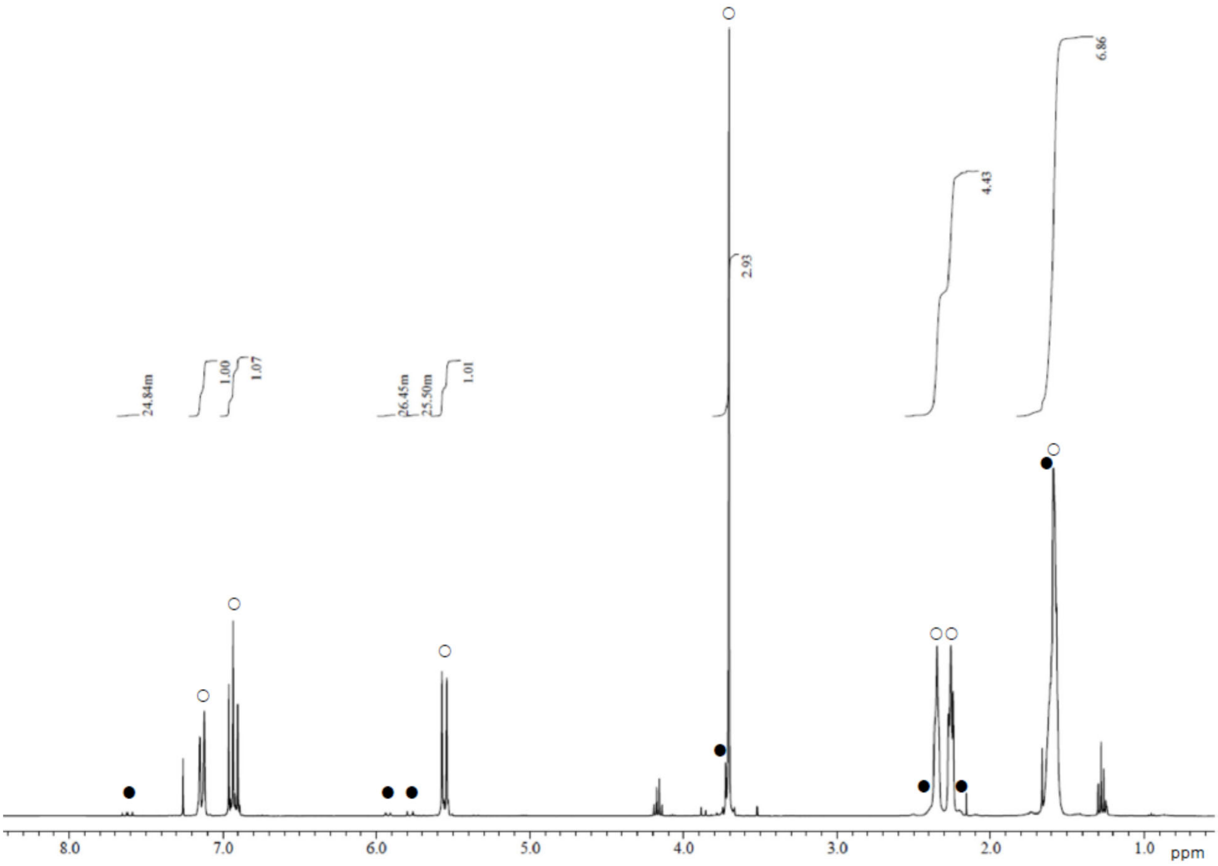

Supplementary Fig 14. ^{1}H NMR spectrum of methyl (*Z*)-4-cyclohexylidenebut-2-enoate (*Z*-**5**) (○) and (*E*)-4-cyclohexylidenebut-2-enoate (*E*-**5**) (●) in $CDCl_3$.

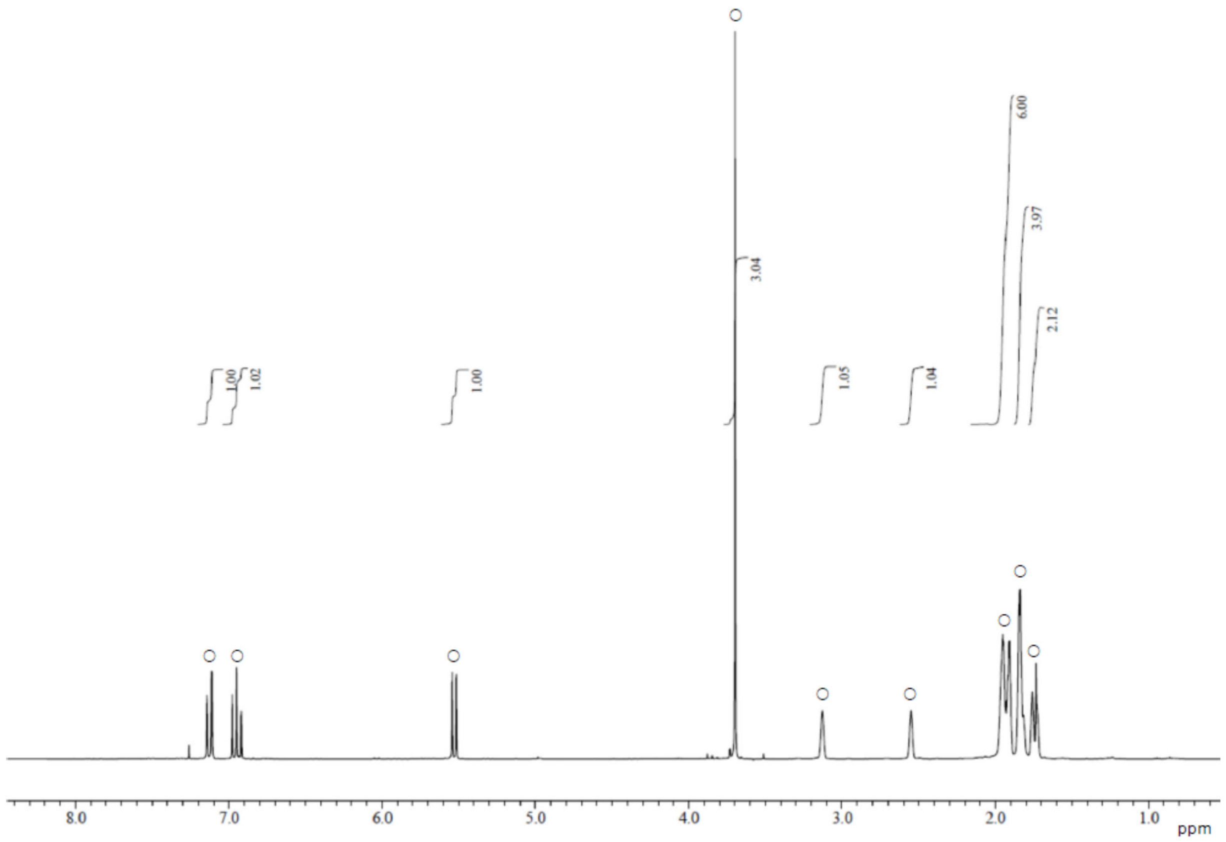

Supplementary Fig 15. ^{1}H NMR spectrum of methyl (*Z*)-4-adamantan-2-ylidenebut-2-enoate (*Z*-**6**) (○) in $CDCl_3$.

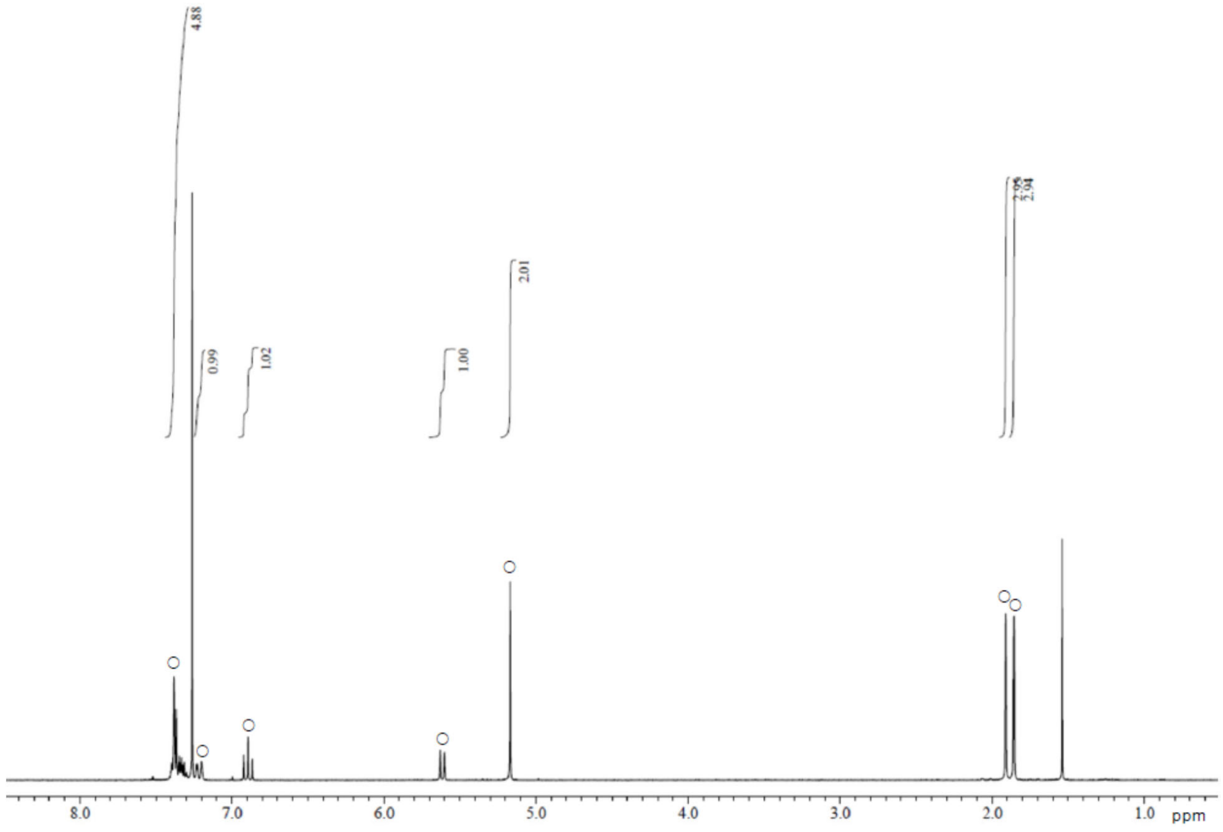

Supplementary Fig 16. ^{1}H NMR spectrum of benzyl (*Z*)-5-methylhexa-2,4-dienoate (*Z*-**7**) (○) in $CDCl_3$.

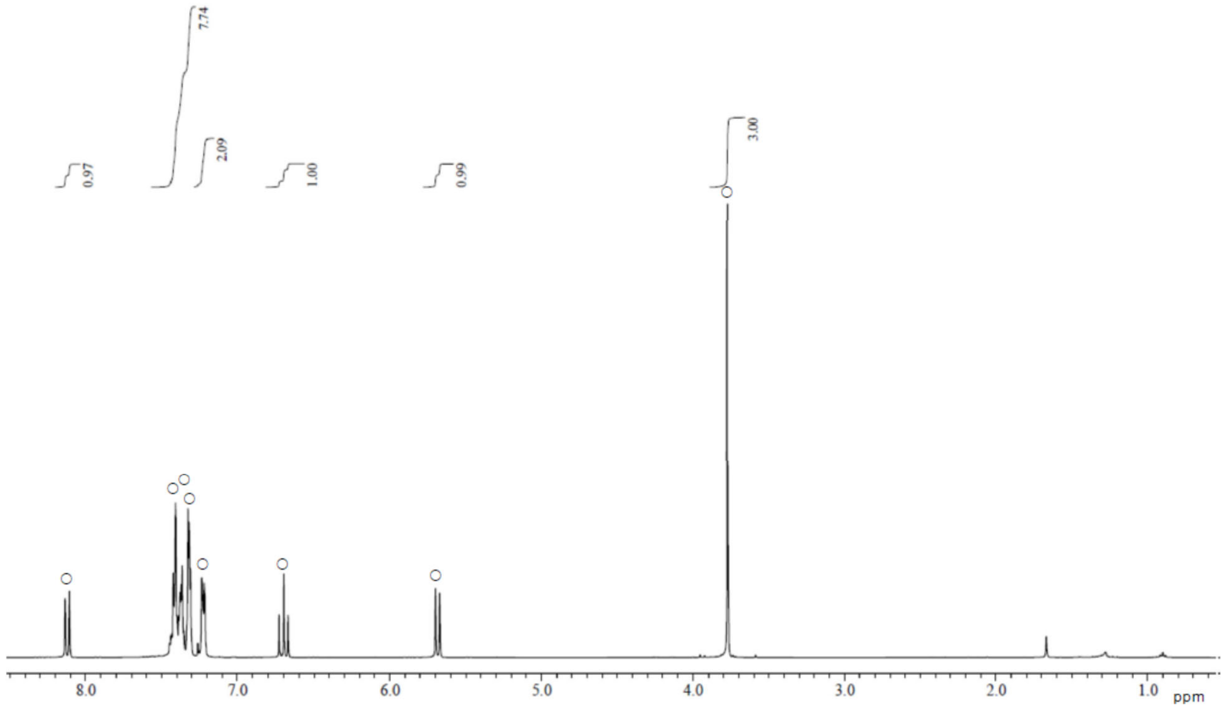

Supplementary Fig 17. ^{1}H NMR spectrum of methyl (*Z*)-5,5-diphenylpenta-2,4-dienoate (*Z*-**8**) (○) in $CDCl_3$.

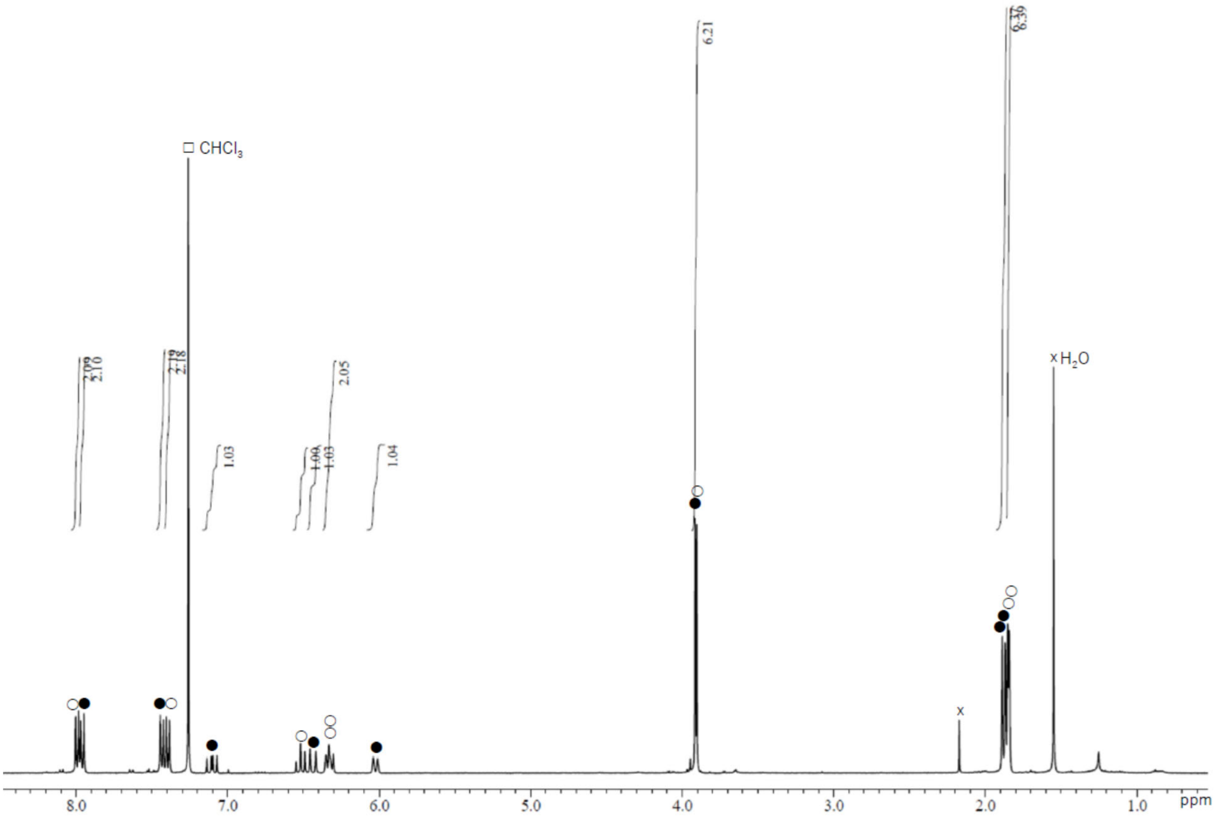

Supplementary Fig 18. ^{1}H NMR spectrum of methyl (*Z*)-4-(4-methylpenta-1,3-dien-1-yl)benzoate (***Z*-9**) (○) in $CDCl_3$. ● = Methyl (*E*)-4-(4-methylpenta-1,3-dien-1-yl)benzoate (***E*-9**) and x = impurities.

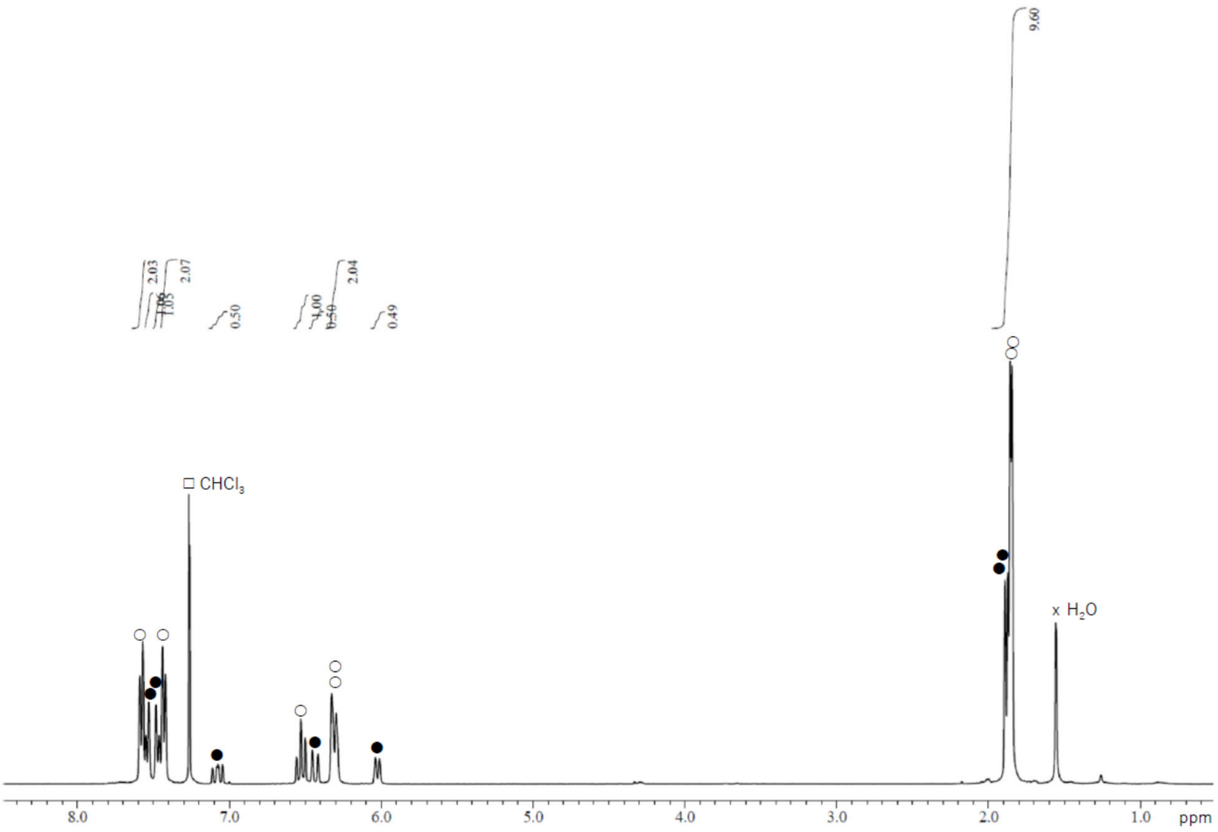

Supplementary Fig 19. ^{1}H NMR spectrum of (*Z*)-1-(4-methylpenta-1,3-dien-1-yl)-4-(trifluoromethyl)benzene (***Z*-10**) (○) in CDCl3. ● = (*E*)-1-(4-methylpenta-1,3-dien-1-yl)-4-(trifluoromethyl)benzene (***E*-10**).

Synthesis of methyl (2*Z*,4*E*)-5-phenylpenta-2,4-dienoate ((2*Z*,4*E*)-**11**)

Methyl (2*Z*,4*E*)-5-phenylpenta-2,4-dienoate ((2*Z*,4*E*)-**11**) was synthesized according to the literature.[16] To a mixture of methyl (2Z)-3-iodoprop-2-enoate (1.44 g, 6.79 mmol), Ag_2CO_3 (2.81 g, 1.02 x 10 mmol), $Pd(OAc)_2$ (76 mg, 3.4 x 10^{-1} mmol) in CH_3CN, styrene (2.89 g, 2.77 x 10 mmol) was added in dark at room temperature. The mixture was stirred for 16 h. The mixture was filtered through Celite, and the filtrate was evaporated *in vacuo*. The resulting solid was diluted with hexane/AcOEt (9:1), and filtered through a silica gel pad. The solvent was removed *in vacuo* and the residue was purified by a silica gel column chromatography to give methyl (2*Z*,4*E*)-5-phenylpenta-2,4-dienoate ((2*Z*,4*E*)-**9**) as colorless oil (0.75 g, 4.0 mmol, 59% yield). ^{1}H NMR (400 MHz, $CDCl_3$, 25 °C) δ 8.14 (dd, ^3J = 15.4 Hz, and 11.2 Hz, 1H, H_4), 7.53 (d, ^3J = 7.2 Hz, 2H, *o*-Ph), 7.58-7.27 (m, 3H, *m*-Ph and *p*-Ph), 6.83 (d, ^3J = 15.2 Hz, 1H, H_5), 6.75 (dd, ^3J = 11.6 Hz, and 11.2 Hz, 1H, H_3), 5.74 (d ^3J = 11.6 Hz, 1H, H_2), 3.77 (s, 3H, OMe). ^{13}C NMR (101 MHz) δ 167.0 (C_1),145.0 (C_3), 141.4 (C_5), 136.3 (Ph), 129.0 (Ph), 128.7 (Ph), 127.5 (Ph), 124.9 (C_4), 117.0 (C_2), 51.2 (OMe). HRMS-ESI (m/z): $[M+Na]^+$ calcd for $C_{12}H_{12}O_2$, 211.0730; found, 211.0764.

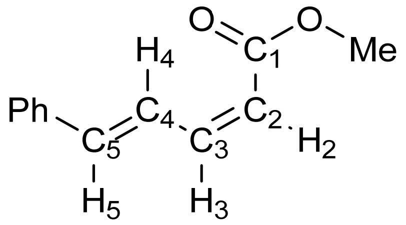

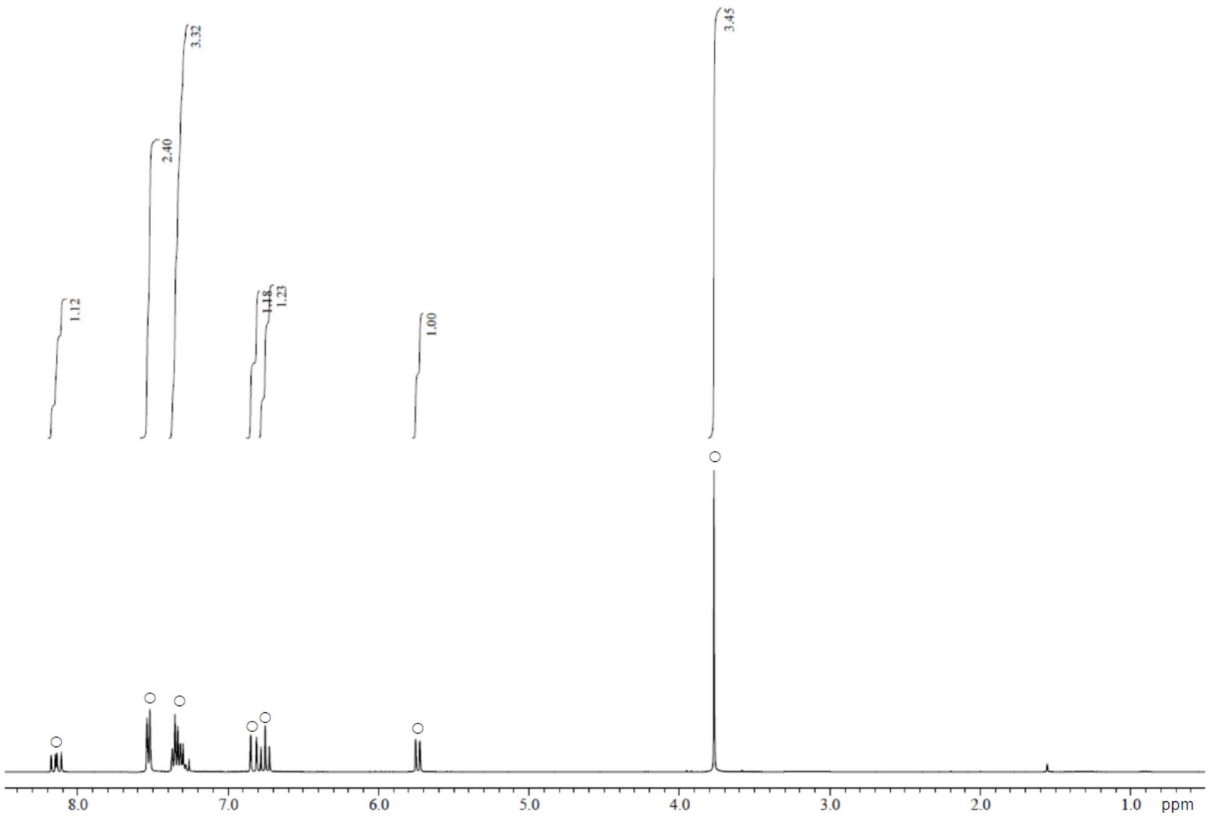

Supplementary Fig 20. ^{1}H NMR spectrum of methyl (2*E*,4*E*)-5-phenylpenta-2,4-dienoate ((2*E*,4*E*)-**11**) (○) in $CDCl_3$.

Generation of $[Pd_2(\mu\text{-}\eta^3\text{:}\eta^1\text{-mpd})(CH_3CN)_5][BF_4]_2$ (**12**) from diene **11**

To a solution of $[Pd_2(CH_3CN)_6][BF_4]_2$ (**1**) (10.0 mg, 1.6 x 10^{-2} mmol) in CD_3NO_2/CD_3CN (v/v = 9/1) was added methyl (2*E*,4*E*)-5-phenylpenta-2,4-dienoate ((2*E*,4*E*)-**11**, 2.9 mg, 1.6 x 10^{-2} mmol). After 10 min at room temperature, the ^{1}H NMR spectra showed the generation of **12-cisoid-antifacial** (73% yield (based on complex **1**), **12-transoid-antifacial**:**12-cisoid-antifacial** = <1 : >99). Generation of **12-transoid-antifacial** by the reaction of complex **1** (10.3 mg, 1.6 x 10^{-2} mmol) with (2*Z*,4*E*)-4-phenyllbuta-2,4-dienoate ((2*Z*,4*E*)-**11**, 2.8 μL, 1.6 x 10^{-2} mmol) was also confirmed by ^{1}H NMR analysis (84% yield (based on complex **1**), **12-transoid-antifacial**:**12-cisoid-antifacial** = >99:<1).

For **12-transoid-antifacial**, ^{1}H NMR (400 MHz, CD_3NO_2/CD_3CN, 25 °C) δ 7.71 (d, ^3J = 7.3 Hz, 2H, *o*-Ph), 7.51 (dd, ^3J = 7.3, 7.3 Hz, 1H, *p*-Ph), 7.43 (dd, ^3J = 7.3, 7.3 Hz, 2H, *m*-Ph), 6.48 (dd, ^3J = 12.0 Hz, and 11.6 Hz, 1H, H_4), 5.02 (d, ^3J = 11.6 Hz, 1H, H_5), 4.62 (dd, ^3J = 12.0 Hz, and 10.4 Hz, 1H, H_3), 4.19 (d, ^3J = 10.4 Hz, 1H, H_2), 3.77 (s, 3H, OMe). $^{13}C\{^1H\}$ NMR (101 MHz, CD_3NO_2, 25 °C) δ 174.5 (C_1), 137.7 (*ipso*-Ph), 130.7 (*m*-Ph), 130.5 (*p*-Ph), 129.4 (*o*-Ph), 109.3 (C_4), 85.5 (C_3), 83.0 (C_5), 52.9 (OMe), 30.8 (C_2).

For **12-cisoid-antifacial**, ^{1}H-NMR (400 MHz, CD_3NO_2/CD_3CN) δ 7.69-7.71 (m, 2H, *o*-Ph), 7.46-7.52 (m, 1H, *p*-Ph), 7.34-7.45 (m, 2H, *m*-Ph), 6.90 (dd, ^3J = 11.6 Hz, 1H, H_4), 4.94 (d, ^3J = 11.6 Hz, 1H, H_5), 4.40-4.30 (m, 1H, H_3), 4.06 (d, ^3J = 6.0 Hz, 1H, H_2), 3.75 (s, 3H, OMe). $^{13}C\{^1H\}$ NMR (101 MHz, CD_3NO_2/CD_3CN, 25 °C) δ 176.0 (C_1), 138.0 (*ipso*-Ph), 130.6 (*m*-Ph), 130.3 (*p*-Ph), 129.7 (*o*-Ph), 110.2 (C_4), 83.4 (C_3), 82.1 (C_5), 52.8 (OMe), 27.5 (C_2).

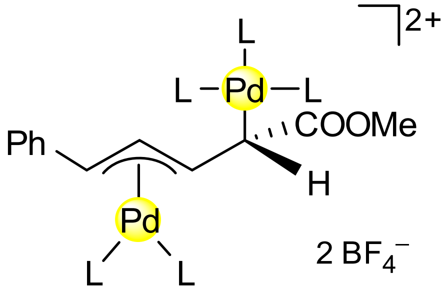

(L = CH_3CN)

12-cisoid-antifacial

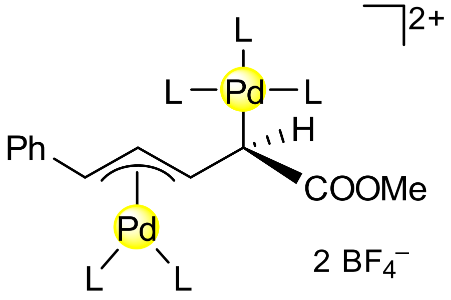

(L = CH_3CN)

12-transoid-antifacial

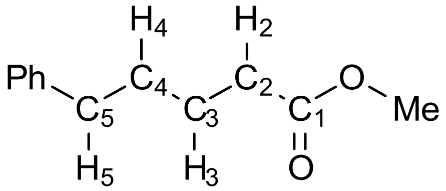

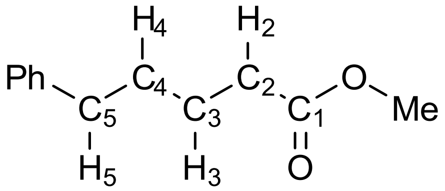

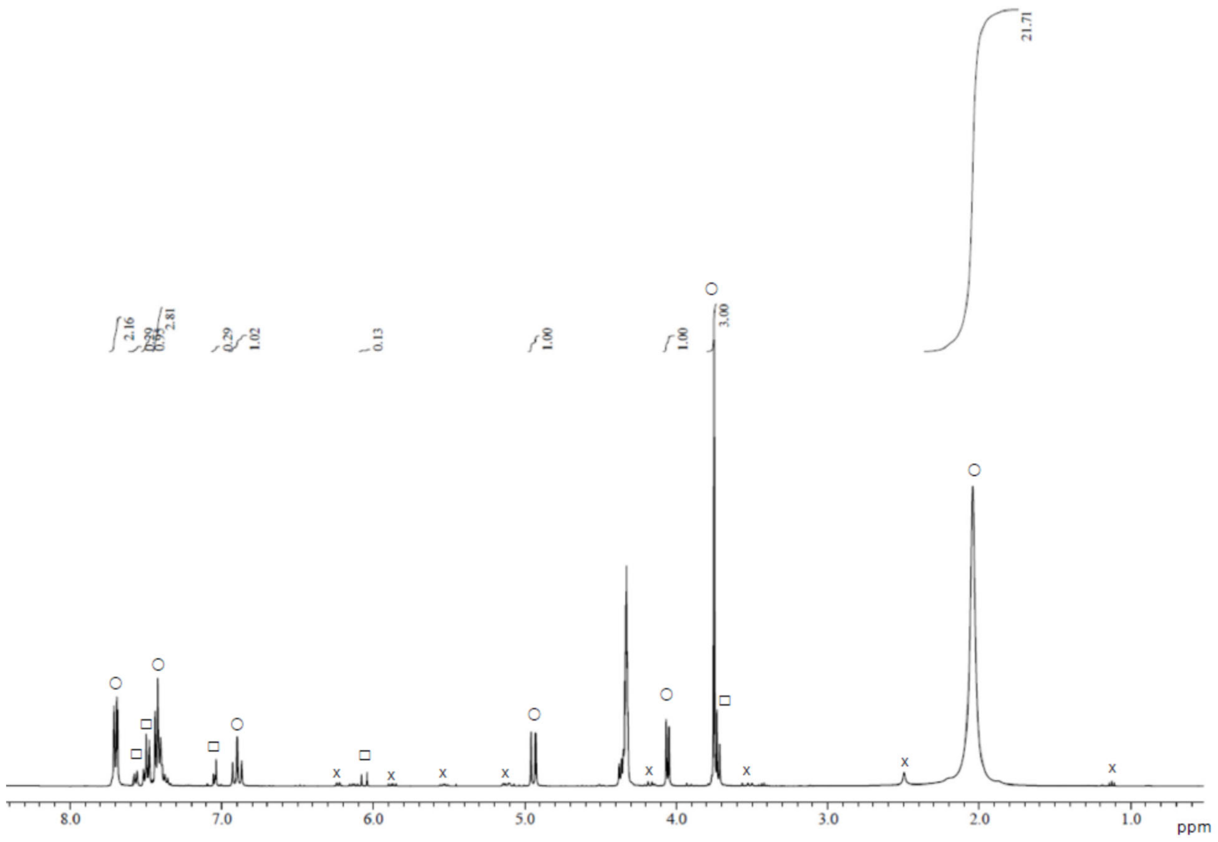

Supplementary Fig 21. ^{1}H NMR spectrum of methyl cisoid-[Pd_2(μ-η^3:η^1-mpd)(CH_3CN)$_5$][BF_4]$_2$ (**12-cisoid-antifacial**) (○) in $CDCl_3$. □ = (2*E*,4*E*)-**11** and x = impurities.

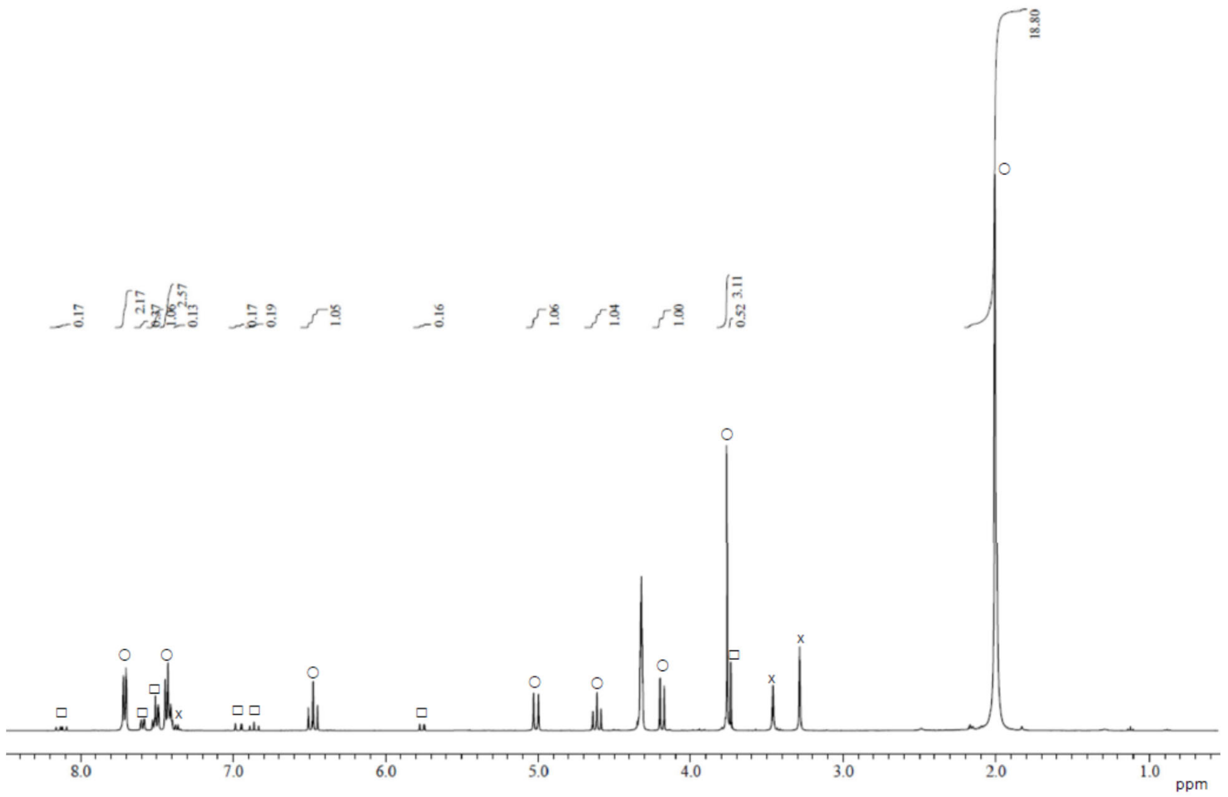

Supplementary Fig 22. ^{1}H NMR spectrum of methyl transoid-[Pd_2(μ-η^3:η^1-mpd)(CH_3CN)$_5$][BF_4]$_2$ (**12-transoid-antifacial**) (○) in $CDCl_3$. □ = (2*Z*,4*E*)-**11** and x = impurities.

Anti-elimination of diene **11** from complex **12** by the addition of TEMPO

A CD_3NO_2/CD_3CN solution (v/v = 9:1) of complex **12-cisoid-antifacial** (**12-transiod-antifacial**:**12-cisoid-antifacial** = <1 : >99) was prepared by the reaction of complex **1** (10.7 mg, 1.69 x 10^{-2} mmol) with diene (2*E*,4*E*)-**11** (3.2 mg, 1.7 x 10^{-2} mmol). 2,2,6,6-tetramethylpiperidine 1-oxyl (TEMPO) (5.2 mg, 3.3 x 10^{-2} mmol) was added to the reaction mixture at -30 ºC. ^{1}H NMR spectra showed increasing intensity of free diene **11** compared to internal standard (dibenzyl). The *E*/*Z* ratio of methyl 5-phenyl-2,4-pentadienoate through diene elimination from **11** was 24:76 (76% yield).

Isolation of $[Pd(\eta^2\text{-TEMPO})(CH_3CN)_2][BF_4]$ (**13**)

To a solution of $[Pd_2(CH_3CN)_6][BF_4]_2$ (**1**, 401.1 mg, 6.34 x 10^{-1} mmol) in CD_3NO_2 was added 2,2,6,6-tetramethylpiperidine 1-oxyl (TEMPO) (203.8 mg, 1.30 mmol, 2.1 equiv.). The reaction mixture was stirred for 1 h at room temperature, and then the mixture was filtered through Celite. The filtrate was dried *in vacuo*. The residual solid was dissolved in CH_3NO_2, and then the solution was reprecipitated with diethyl ether. The precipitated was washed with diethyl ether and dried *in vacuo* to give **13** (495.5 mg, 1.14 mmol, 91% yield) as an orange solid. The yellow single crystals of **13** were grown from CH_2Cl_2-mesitylene solution at -30 ˚C.

^{1}H NMR (400 MHz, CD_3NO_2, 25 °C) δ 2.41 (s, 3H, MeCN), 2.35 (s, 3H, MeCN), 2.06 (s, 6H, H_4 or H_5), 1.90-1.80 (m, 3H, H_2 or H_2’ and H_3 or H_3’), 1.66-1.56 (m, 3H, H_2 or H_2’ and H_3 or H_3’), 1.46 (s, 6H, H_4 or H_5). $^{13}C\{^1H\}$ NMR (101 MHz, CD_3NO_2, 25 °C) δ 127.5 (MeC̲N), 126.6 (MeC̲N), 73.2 (C_1), 39.1 (C_2), 32.1 (C_4 or C_5), 25.7 (C_4 or C_5), 17.4 (C_3), 3.17 (M̲e̲CN), 2.88 (M̲e̲CN). Anal. Calcd for $PdC_{13}H_{24}N_3OBF_4$: C, 36.18; H, 5.61; N, 9.74. Found: C, 35.88; H, 5.82; N, 9.46.

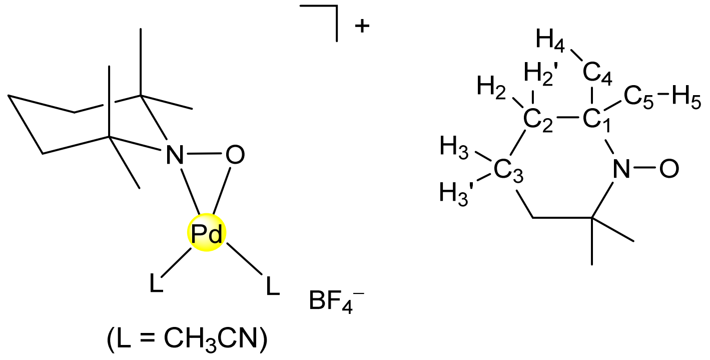

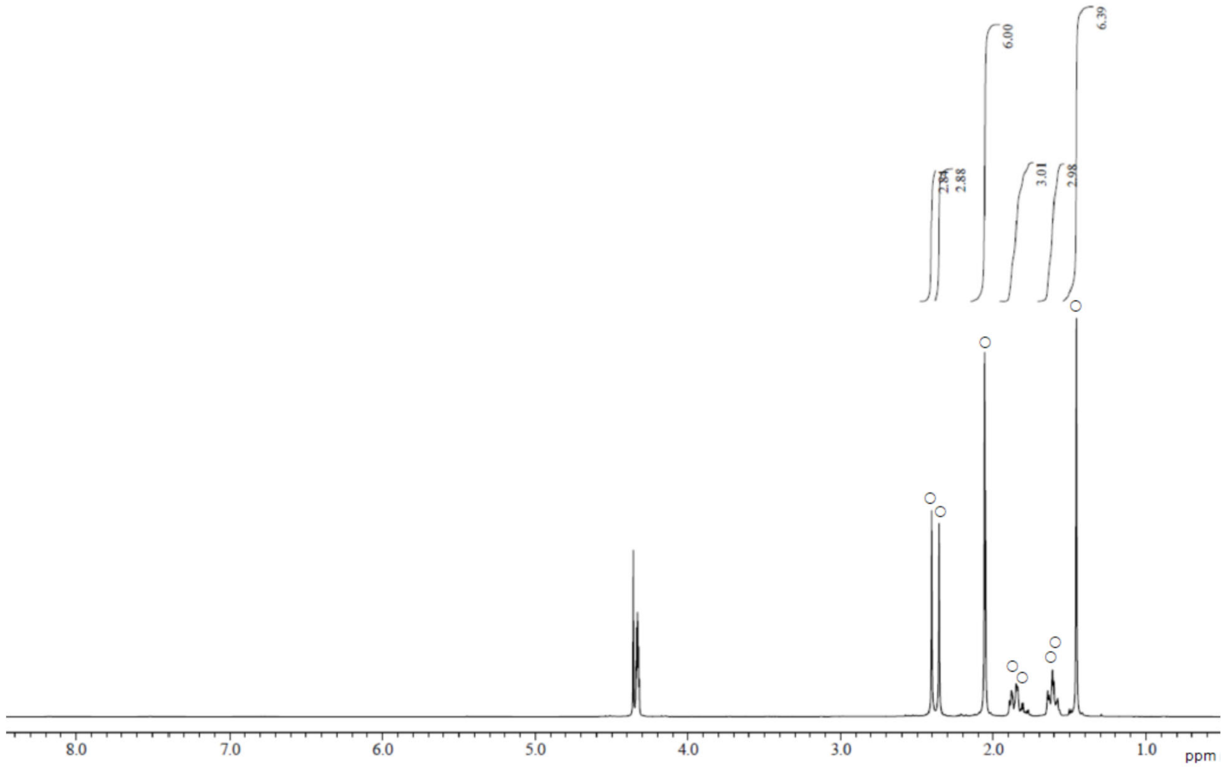

Supplementary Fig 23. ^{1}H NMR spectrum of [Pd(η^2-TEMPO)(CH_3CN)$_2$][BF_4] (**13**) (○) in CD_3NO_2.

E to Z isomerization of dienes through *anti*-elimination of transoid-antifacial-Pd_2 complexes by using TEMPO

Methyl (2*Z*,4*E*)-5-phenylpenta-2,4-dienoate ((2*Z*,4*E*)-**11**)

To a solution of $[Pd_2(CH_3CN)_6][BF_4]_2$ (**1**, 168.1 mg, 2.66 x 10^{-1} mmol) in CH_3NO_2/CH_3CN (v/v = 95:5) was added the methyl (*E*)-5-methylhexa-2,4-dienoate (50.0 mg, 2.66 x 10^{-1} mmol) and stirred at room temperature for 15 min. The reaction mixture was cooled to -20 °C and a CH_3NO_2/CH_3CN (v/v = 95:5) solution of TEMPO (167.7 mg, 1.07 mmol, 4 equiv.) was added at -20 °C. After stirring 5 min, the mixture was diluted with diethyl ether, and filtered through Celite. The solution was washed with distilled water (2 times). The organic layer was collected, and the aqueous layer was extracted with Et_2O (2 times). The combined organic layers were dried with $MgSO_4$. The solvent was removed *in vacuo* and the residue was purified by a silica gel column chromatography and recycling size exclusion chromatography to give methyl (2*Z*,4*E*)-5-phenylpenta-2,4-dienoate ((2*Z*,4*E*)-**11**) as colorless oil (28.2 mg, 1.50 x 10^{-1} mmol, 56% yield, *E* : *Z* = <1 : >99).

The following *E* to *Z* isomerization trough *anti*-elimination were performed according to the procedure for *E* to *Z* isomerization of methyl (2*Z*,4*E*)-5-phenylpenta-2,4-dienoate.

Methyl (2*Z*,4*E*)-5-(4-nitrophenyl)penta-2,4-dienoate ((2*Z*,4*E*)-**14**)

Methyl (2*E*,4*E*)-5-(4-nitrophenyl)penta-2,4-dienoate ((2*E*,4*E*)-**14**, 50.0 mg, 2.14 x 10^{-1} mmol), $[Pd_2(CH_3CN)_6][BF_4]_2$ (**1**, 135.7 mg, 2.14 x 10^{-1} mmol) and TEMPO (134.0 mg, 8.58 x 10^{-1} mmol, 4 equiv.) were used. Methyl (2*Z*,4*E*)-5-(4-nitrophenyl)-penta-2,4-dienoate ((2*Z*,4*E*)-**14**) was obtained as colorless oil (13.4 mg, 5.75 x 10^{-2} mmol, 27% yield, *E* : *Z* = <1 : >99). ^{1}H NMR (400 MHz, $CDCl_3$, 25 °C) δ 8.29 (dd, ^3J = 16.0 Hz, and 10.8 Hz, 1H, H_4), 8.21 (d, ^3J = 8.8 Hz, 2H, *m*-Ar), 7.65 (d, ^3J = 8.8 Hz, 2H, *o*-Ar), 6.85 (d, ^3J = 16.0 Hz, 1H, H_5), 6.76 (dd, ^3J = 10.8 Hz, and 10.8 Hz, 1H, H_3), 5.87 (d ^3J = 10.8 Hz, 1H, H_2), 3.79 (s, 3H, H_{10}). ^{13}C NMR (101 MHz) δ 166.6 (C_1), 147.6 (C_9), 143.5 (C_3), 142.6 (C_6), 138.0 (C_5), 128.8 (C_4), 127.9 (C_7), 124.1 (C_8), 119.8 (C_2), 51.4 (C_{10}). HRMS-ESI (m/z): $[M+Na]^+$ calcd for $C_{12}H_{11}NO_4$, 256.0680; found, 256.0451.

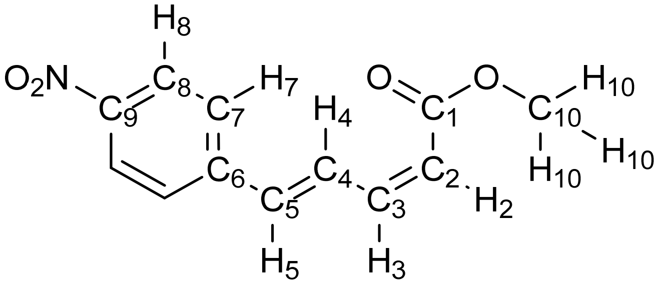

Methyl (2*Z*,4*E*)-5-(4-methoxyphenyl)penta-2,4-dienoate ((2*Z*,4*E*)-**15**)

Methyl (2*E*,4*E*)-5-(4-methoxyphenyl)penta-2,4-dienoate ((2*E*,4*E*)-**15**, 50.0 mg, 2.29 x 10^{-1} mmol), $[Pd_2(CH_3CN)_6][BF_4]_2$ (**1**, 145.0 mg, 2.29 x 10^{-1} mmol) and TEMPO (143.2 mg, 9.16 x 10^{-1} mmol, 4 equiv.) were used. Methyl (2*Z*,4*E*)-5-(4-methoxyphenyl)-penta-2,4-dienoate ((2*Z*,4*E*)-**15**) was obtained as colorless oil (22.1 mg, 1.01 x 10^{-1} mmol, 44% yield, *E* : *Z* = <1 : >99). ^{1}H NMR (400 MHz, $CDCl_3$, 25 °C) δ 8.02 (dd, ^3J = 16.0 Hz, and 11.6 Hz, 1H, H_4), 7.48 (d, ^3J = 9.2 Hz, 2H, *o*-Ar), 6.88 (d, ^3J = 9.2 Hz, 2H, *m*-Ar), 6.79 (d, ^3J = 16.0 Hz, 1H, H_5), 6.74 (dd, ^3J = 11.6 Hz, and 11.6 Hz, 1H, H_3), 5.68 (d ^3J = 11.6 Hz, 1H, H_2), 3.83 (s, 3H, H_{11}), 3.76 (s, 3H, H_{10}). ^{13}C NMR (101 MHz) δ 167.2 (C_1), 160.4 (C_9), 145.5 (C_3), 141.2 (C_5), 129.1 (C_6), 129.0 (C_7), 122.8 (C_4), 115.6 (C_2), 114.2 (C_8), 55.3 (C_{11}), 51.1 (C_{10}). HRMS-ESI (m/z): $[M+Na]^+$ calcd for $C_{13}H_{14}O_3$, 241.0835; found, 241.0958.

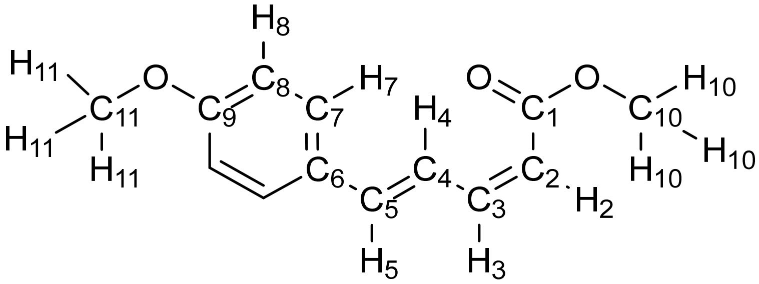

Methyl (2*Z*,4*E*)-6,6-dimethylhepta-2,4-dienoate ((2*Z*,4*E*)-**16**)

Methyl (2*E*,4*E*)-6,6-dimethylhepta-2,4-dienoate ((2*E*,4*E*)-**16**, 50.0 mg, 2.97 x 10^{-1} mmol), $[Pd_2(CH_3CN)_6][BF_4]_2$ (**1**, 190.0 mg, 2.97 x 10^{-1} mmol) and TEMPO (187.6 mg, 1.20 mmol, 4 equiv.) were used. After addition of TEMPO, the reaction mixture was stirred for 30 min. at room temperature. Methyl (2*Z*,4*E*)-6,6-dimethylhepta-2,4-dienoate ((2*Z*,4*E*)-**16**) was obtained as colorless oil (31.8 mg, 1.89 x 10^{-1} mmol, 64% yield, *E* : *Z* = <1 : >99). ^{1}H NMR (400 MHz, $CDCl_3$,

25 °C) δ 7.32 (dd, ^3J = 16.0 Hz, and 11.2 Hz, 1H, H_4), 6.56 (dd, ^3J = 11.2 Hz, and 11.2 Hz, 1H, H_3), 6.07 (d, ^3J = 16.0 Hz, 1H, H_5), 5.59 (d, ^3J = 11.2 Hz, 1H, H_2), 3.72 (s, 3H, H_8), 1.06 (s, 9H, H_7). ^{13}C NMR (101 MHz) δ 167.1 (C_1), 156.3 (C_5), 146.3 (C_3), 121.9 (C_4), 115.2 (C_2), 51.0 (C_8), 34.0 (C_6), 29.1 (C_7). HRMS-ESI (m/z): $[M+Na]^+$ calcd for $C_{10}H_{16}O_2$, 191.1043; found, 191.1207.

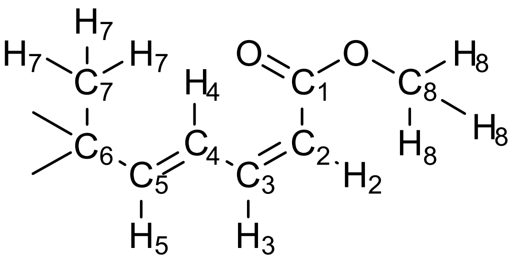

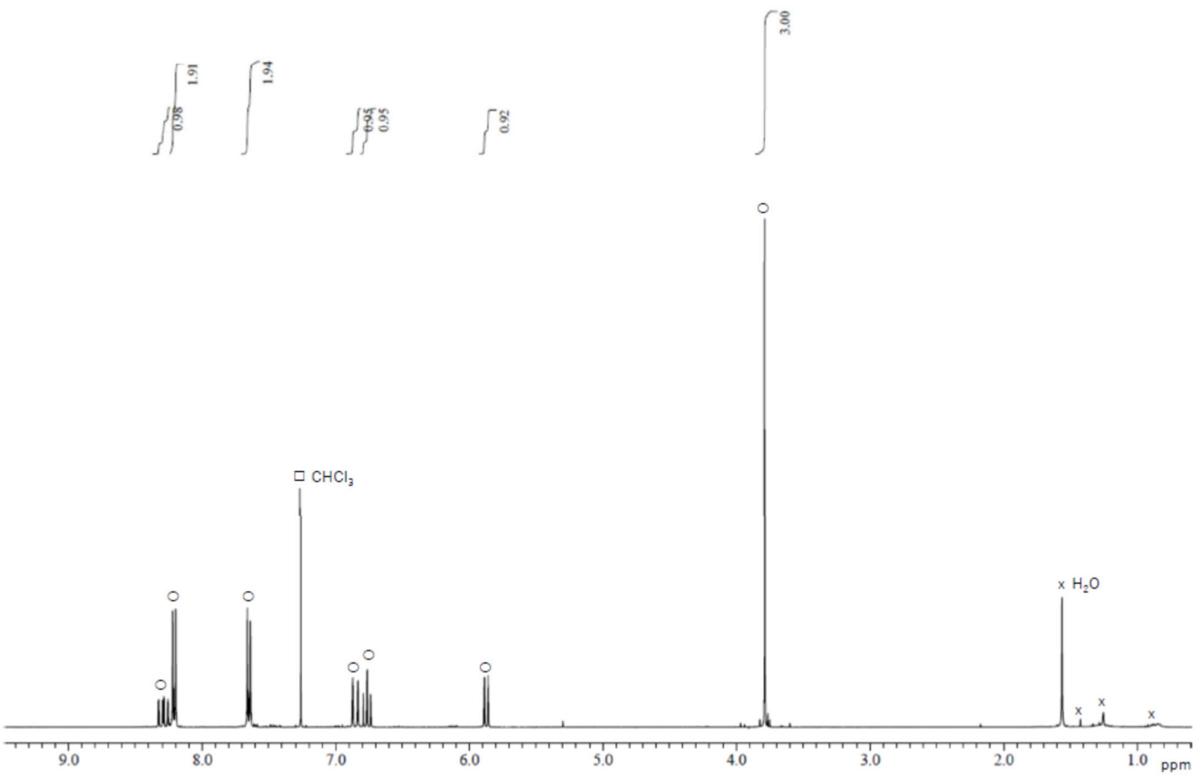

Supplementary Fig 24. ^{1}H NMR spectrum of methyl (2*Z*,4*E*)-5-(4-nitrophenyl)penta-2,4-dienoate ((2*Z*,4*E*)-**14**) (○) in $CDCl_3$. x = impurities.

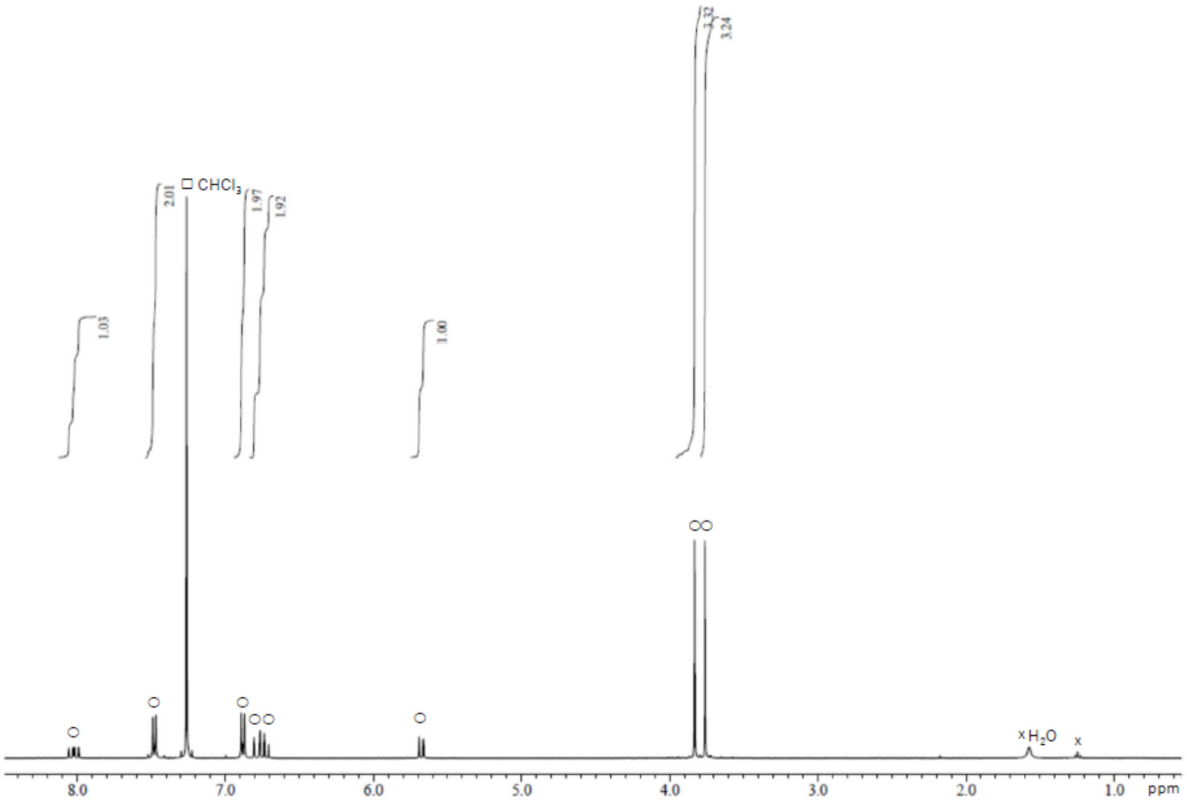

Supplementary Fig 25. ^{1}H NMR spectrum of methyl (2*Z*,4*E*)-5-(4-methoyphenyl)penta-2,4-dienoate ((2*Z*,4*E*)-**15**) (○) in $CDCl_3$. x = impurities.

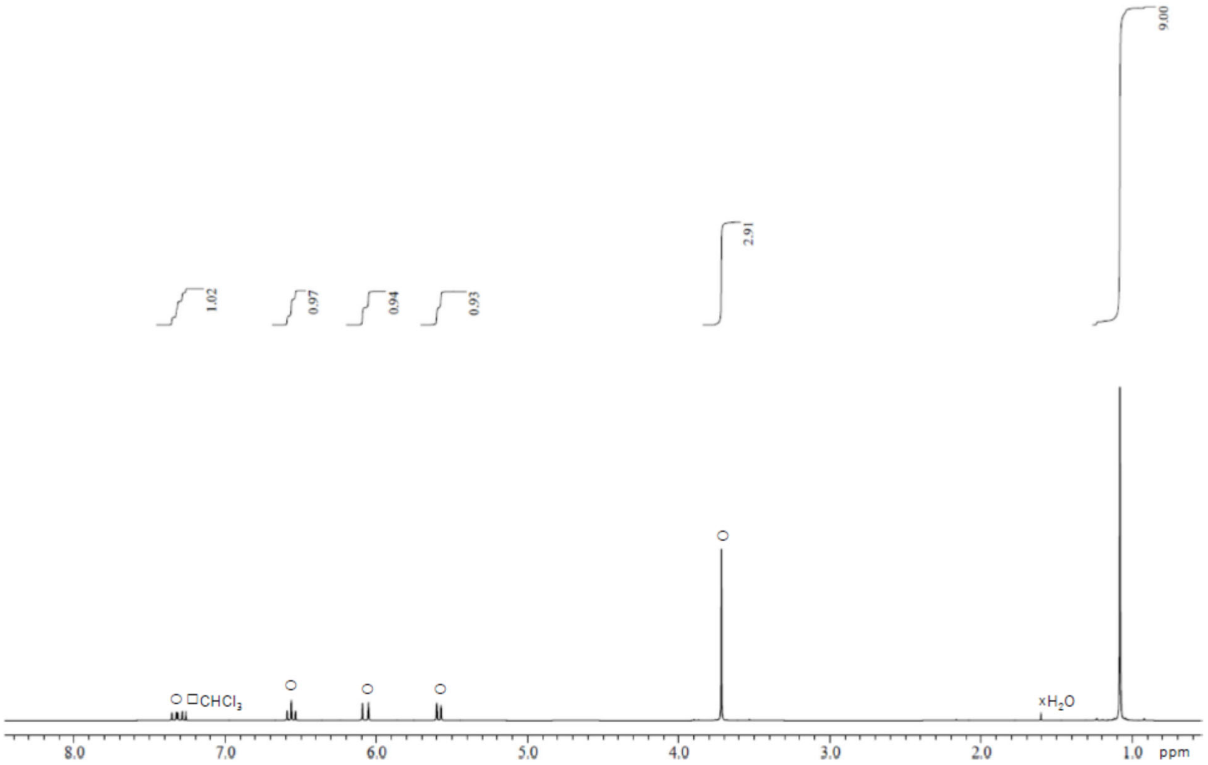

Supplementary Fig 26. ^{1}H NMR spectrum of methyl (2*Z*,4*E*)-6,6-dimethylhepta-2,4-dienoate ((2*Z*,4*E*)-**16**) (○) in $CDCl_3$.

Synthesis of $Pd_2(dba)_3$ and 9-aza-barbaralane **17** through double amination of complex **4**

To a solution of $[Pd_2(CH_3CN)_6][BF_4]_2$ (**1**, 100.0 mg, 1.60 x 10^{-1} mmol) in CH_3CN was added the COT (17.3 mg, 1.70 x 10^{-1} mmol) and stirred at room temperature. After 30 min, dba (185.1 mg, 7.90 x 10^{-1} mmol) was added to the reaction mixture, and then the reaction mixture was cooled at 0 °C. After addition of pyrrolidine (22.4 mg, 3.20 x 10^{-1} mmol) at 0 °C, the reaction mixture was stirred at the temperature for 2 h. The violet precipitate in suspension was then separated by decantation, and the supernatant was *in vacuo*. The solution was diluted with $CHCl_3$, and filtered through a silica gel pad. The silica gel pad was washed with $CHCl_3$. The product was extracted by MeOH/$CHCl_3$ (v/v=1:5) form silica gel pad, and concentrated *in vacuo*. The crude product was purified by a silica gel column chromatography (MeOH/$CHCl_3$ (v/v=1:10)) to give 9-aza-barbaralane **17** (24.2 mg, 9.00 x 10^{-2} mmol, 58% yield**)** as a red solid. The single crystal suitable for X-ray diffraction analysis was obtained by recrystallization from CH_2Cl_2/diethyl ether at -30 °C. The violet precipitate separated by decantation was washed with CH_3CN and diethyl ether and dried in vacuo to yield $Pd_2(dba)_3$ (**18**) in 71% yield (Yield was determined by the free dba / $Pd_2(dba)_3$ molar ratio from ^{1}H NMR according to the literature[17]).

For aza-barbaralane **17**: ^{1}H NMR (400 MHz, CD_2Cl_2, 25 °C) δ 6.11-6.01(m, 2H, H_3), 4.65-4.50 (m, 4H, H_2), 4.42-4.31 (m, 2H, H_1), 3.44-3.30 (m, 4H, H_4), 2.30-2.15 (m, 4H, H_5). ^{13}C NMR (101 MHz) δ 122.9 (C_3),73.5 (C_2), 63.0 (t, $^1J_{CN}$ = 3.1 Hz, C_4), 57.1 (t, $^1J_{CN}$ = 3.1 Hz, C_1), 23.1 (C_5). Anal. Calcd. for $C_{12}H_{16}BF_4N$: C, 55.21; H, 6.18 N, 5.37. Found: C, 55.16; H, 6.23 N, 5.26.

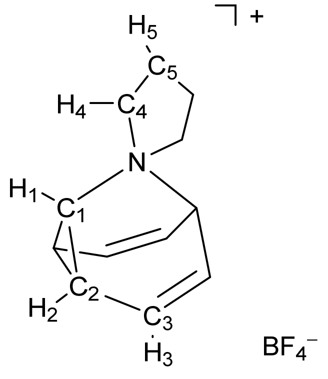

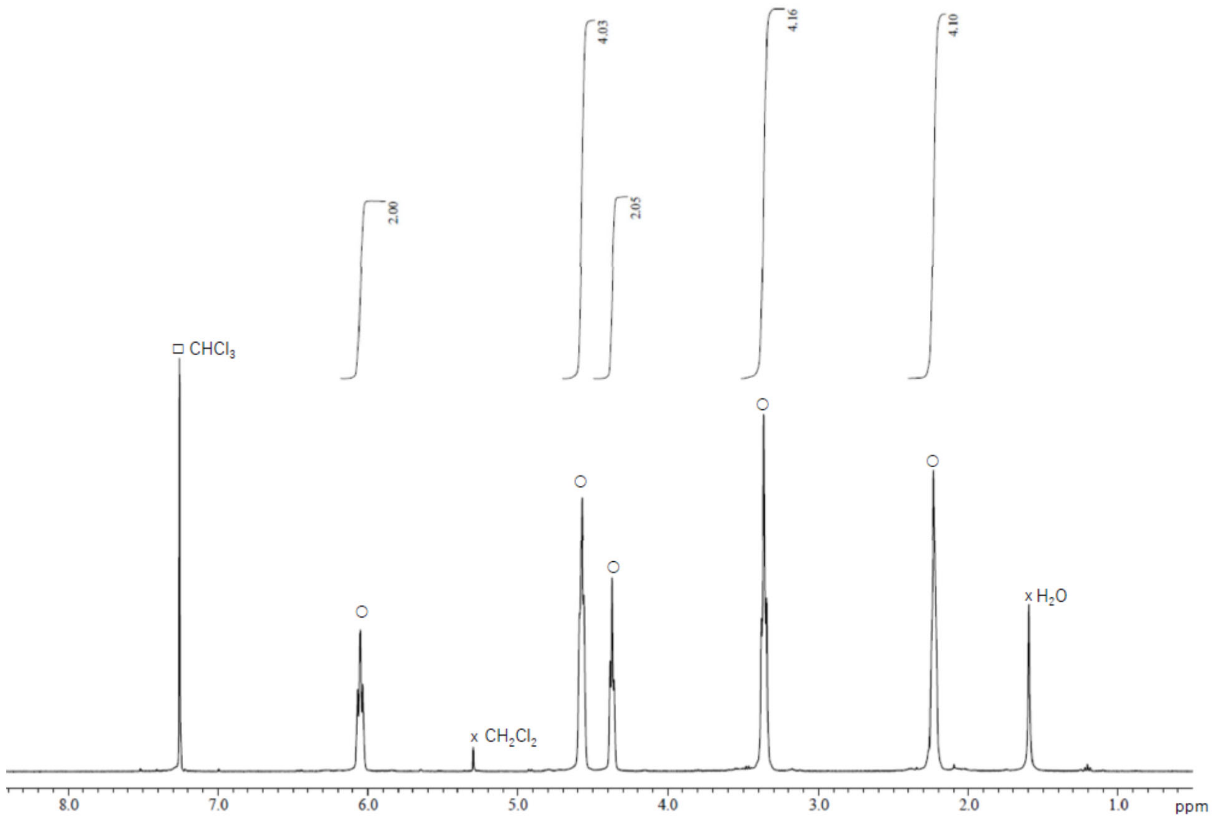

Supplementary Fig 27. ^{1}H NMR spectrum of aza-barbaralane **17** (○) in CD_3NO_2. □ = $CHCl_3$ and x = impurities.

Synthesis of Complex **1** through the oxidation of $Pd_2(dba)_3$ (**18**)

To a suspension of $Pd_2(dba)_3$ (**18**, 268.7 mg, 2.21 x 10^{-1} mmol, prepared in aza-barbaralane synthesis) in CH_3CN/CH_2Cl_2 was added $[Cp_2Fe][BF_4]$ (132.5 mg, 4.86 x 10^{-1} mmol, 2.2 eq.) at room temperature. After stirring for 4 h, the reaction mixture was filtered through Celite. The filtrate was reprecipitated with diethyl ether. The precipitate was washed with diethyl ether and dried in vacuo to give complex **1** (126.5 mg, 2.00 x 10^{-1} mmol, 90% yield).

Synthesis of $[Pd(CH_3CN)_4][BF_4]_2$ (**19**) by protonation of TEMPO-adduct **13**

To an acetonitrile solution of $[Pd(\eta^2\text{-TEMPO})(CH_3CN)_2][BF_4]$ (**13**, 174 mg, 4.03 x 10^{-1} mmol) was added $HBF_4{\cdot}OEt_2$ (115 mg, 7.10 x 10^{-1} mmol, 1.8 eq.) at room temperature. The reaction mixture was stirred for 1 h, and then the mixture was filtered through Celite. The filtrate was concentrated and reprecipitated with diethyl ether. The solid was washed with diethyl ether and dried in vacuo to give $[Pd(CH_3CN)_4][BF_4]_2$ (**19**, 144 mg, 3.24 x 10^{-1} mmol, 80% yield). ^{1}H NMR (400 MHz, CD_3NO_2, 25 °C) δ 2.66 (s, 12H, MeCN).

Synthesis of $[Pd_2(CH_3CN)_6][BF_4]_2$ (**1**) by reduction of $[Pd(CH_3CN)_4][BF_4]_2$ (**19**)

To an acetonitrile solution of $[Pd(CH_3CN)_4][BF_4]_2$ (**19**, 150 mg, 3.38 x 10^{-1} mmol) was added Cp_2Fe (115 mg, 3.41 x 10^{-1} mmol, 1.0 eq.) at room temperature. The reaction mixture was stirred for 15 min, and then the mixture was filtered through Celite. The filtrate was concentrated and reprecipitated with diethyl ether. The solid was dissolved in acetonitrile and reprecipitated with diethyl ether. This reprecipitation procedure was repeated several times. The solid was washed with diethyl ether and dried in vacuo to give $[Pd_2(CH_3CN)_6][BF_4]_2$ (**1**, 104 mg, 1.64 x 10^{-1} mmol, 97% yield). ^{1}H NMR (400 MHz, CD_3NO_2, 25 °C) δ 2.50 (s, 12H, MeCN), 2.17 (s, 6H, MeCN).

Computational Details

All calculations were carried out with Gaussian09 program package (Revision D.01).[18] Geometrical optimization was performed with DFT (B3LYP) method.[19-23] Core electrons of Pd were replaced with Stuttgart-Dresden-Bonn relativistic effect core potentials (ECPs) and its valence electrons were represented by (8s7p6d)/[6s5p3d] basis set.[24] 6-311G(d) basis sets were used.[25,26] The optimized structures of **3-transoid-antifacial-HCN**, **3-cisoid-antifacial-HCN**, *Z*-**2**, and *E*-**2** were shown in Supplementary Figures 28-31. Cartesian coordinates of the optimized geometries were shown in Supplementary Tables 2-5. Gibbs free energies of the compounds were evaluated at 298.15 K and 1 atm, and summarized in Supplementary Table 6.

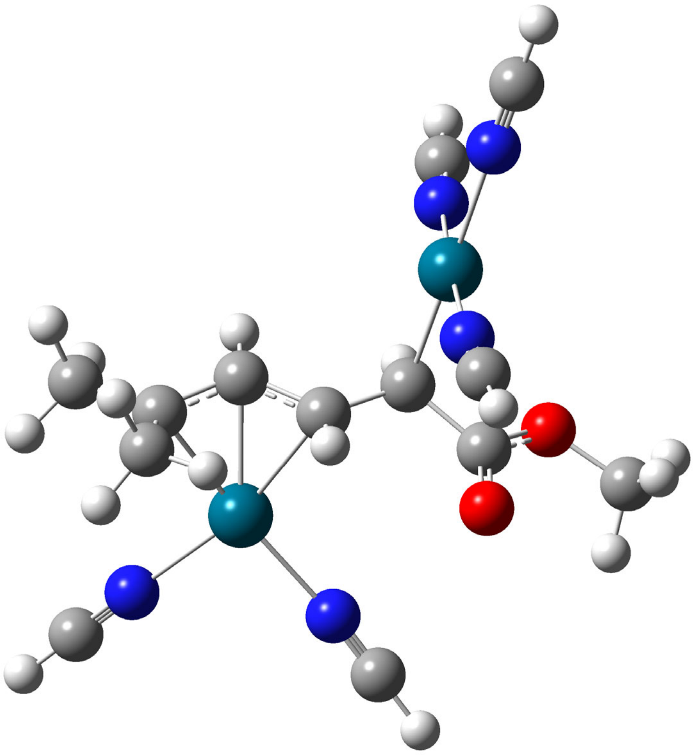

Supplementary Fig 28. The optimized structure of **3-transoid-antifacial-HCN**.

Supplementary Table 2. Cartesian coordinates (in Å) of the optimized geometry of **3-transoid-antifacial-HCN**.

Symbol	X	Y	Z
Pd	2.5385840	0.0057290	-0.0765890
Pd	-2.5083900	-0.4078810	-0.0732700
N	4.6454360	-0.2475270	-0.4145230
N	2.7558560	2.1330460	-0.0369850
N	-2.3922470	-0.0103090	1.9293110
N	-4.4637330	-1.3723800	0.2244780
N	-2.6688530	-0.6908490	-2.0896560
C	2.0557510	-2.1486210	0.3296540
C	0.9685470	-1.4651960	-0.2649400
C	0.3906070	-0.3142780	0.3224750
C	-0.6327850	0.4895660	-0.3841850
C	5.7694850	-0.3422020	-0.6124560
C	2.9548080	3.2545210	0.0780030
C	-2.4232640	0.2596340	3.0400380
C	-5.4860980	-1.8628100	0.3805300
C	-2.8691830	-0.8303940	-3.2072830
H	0.4158400	-0.1882500	1.3993510
H	-0.5234340	0.4753880	-1.4657590
H	-6.4448500	-2.3222130	0.5267680
H	-2.4478600	0.5202080	4.0804920
H	-3.0605220	-0.9599530	-4.2553350
H	3.1424240	4.3040860	0.1885750
H	6.8221450	-0.4308670	-0.7981890
C	-0.7985080	1.9031290	0.1205130
O	-0.4052400	2.2987830	1.1925260
O	-1.4510410	2.6496900	-0.7792680
C	-1.7250480	4.0237510	-0.4113180
H	-2.2429820	4.4514160	-1.2642980
H	-2.3523530	4.0565130	0.4788450
H	-0.7938930	4.5550580	-0.2197600
H	0.7311230	-1.6936000	-1.3014280
C	2.7781990	-3.1888840	-0.4890550
H	3.8311720	-3.2539790	-0.2104110
H	2.3417970	-4.1744430	-0.2881630
H	2.7117530	-3.0034310	-1.5616220
C	2.2381300	-2.2856410	1.8234720
H	1.8714230	-3.2736730	2.1273900
H	3.2941820	-2.2505360	2.0979410
H	1.7063040	-1.5398270	2.4120060

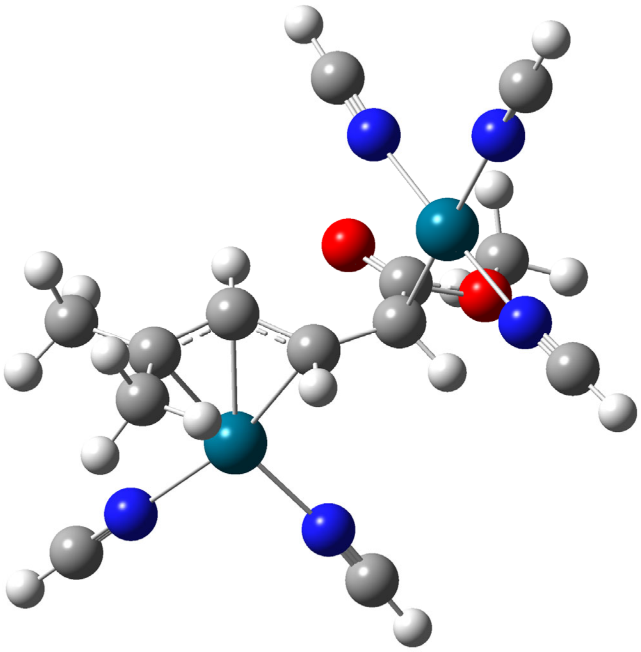

Supplementary Fig 29. The optimized structure of **3-cisoid-antifacial-HCN**.

Supplementary Table 3. Cartesian coordinates (in Å) of the optimized geometry of **3-cisoid-antifacial-HCN**.

Symbol	X	Y	Z
Pd	-2.4970650	0.0282410	-0.2959160
Pd	2.5731700	-0.2209730	-0.2545090
N	-4.6250360	-0.1098850	-0.0287590
N	-2.6827340	2.0270770	-1.0582050
N	2.4057910	-0.9631700	1.6371420
N	4.5680230	-1.1342510	-0.4282510
N	2.7849850	0.5812430	-2.1207260
C	-2.0162700	-2.1125310	0.1907510
C	-0.9626740	-1.2362150	0.5499020
C	-0.3389750	-0.4066000	-0.4146590
C	0.6613160	0.6569760	-0.1368280
C	-5.7596390	-0.1524120	0.1215040
C	-2.8999390	3.0826620	-1.4462680
C	2.4080450	-1.3524170	2.7120970
C	5.6124770	-1.5957190	-0.5072950
C	3.0163110	1.0180060	-3.1524780
H	-0.3124680	-0.7554420	-1.4435520
H	6.5920220	-2.0278550	-0.5811300
H	2.4028510	-1.7092000	3.7237570
H	3.2377560	1.4273250	-4.1195370
H	-3.1080420	4.0704780	-1.8077540
H	-6.8220060	-0.1929600	0.2632270
H	0.7059600	1.3688120	-0.9565430
C	0.5918270	1.4050320	1.1738090
O	0.1364370	0.9721320	2.2079010
O	1.1211120	2.6257310	1.0338010
C	1.1561410	3.4586580	2.2206680
H	1.7598090	2.9842230	2.9934900
H	1.6061190	4.3933190	1.9003080
H	0.1462720	3.6203670	2.5946500
H	-0.8064370	-1.0104140	1.5980960
C	-2.8123420	-2.7515770	1.3013890
H	-3.8431170	-2.9396560	0.9965690
H	-2.3768960	-3.7289410	1.5413980
H	-2.8196750	-2.1521780	2.2121640
C	-2.1033430	-2.8329560	-1.1349450
H	-1.7563850	-3.8635250	-0.9919270
H	-3.1367880	-2.9011760	-1.4803470
H	-1.5050250	-2.3929560	-1.9312040

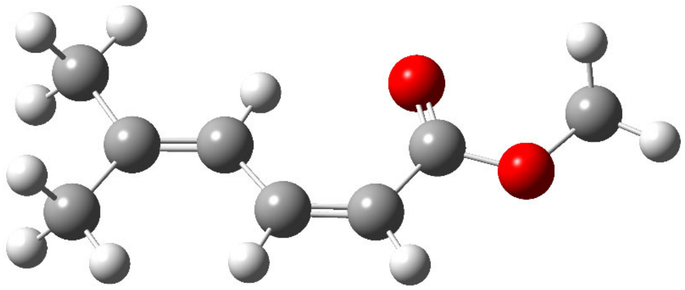

Supplementary Fig 30. The optimized structure of *Z*-**2**.

Supplementary Table 4. Cartesian coordinates (in Å) of the optimized geometry of *Z*-**2**.

Symbol	X	Y	Z
C	-3.3414370	-1.5161850	0.0000120
H	-3.9919440	-1.6178560	0.8771800
H	-2.6379550	-2.3498480	0.0000420
H	-3.9919330	-1.6178990	-0.8771560
C	-2.6407830	-0.1841210	-0.0000090
C	-3.5645860	1.0037780	-0.0000190
H	-4.2225960	0.9759820	-0.8763780
H	-4.2223640	0.9761900	0.8765210
H	-3.0533630	1.9654050	-0.0002030
C	-1.2905390	-0.1288060	-0.0000040
H	-0.7359320	-1.0596470	-0.0000010
C	-0.4931060	1.0771570	0.0000100
H	-1.0271880	2.0236600	0.0000200
C	0.8551960	1.1716640	0.0000170
H	1.3177820	2.1518530	0.0000340
C	1.7990380	0.0418280	0.0000070
O	1.5414260	-1.1430900	-0.0000030
O	3.0758670	0.5085170	0.0000030
C	4.0996780	-0.4940180	-0.0000150
H	4.0223580	-1.1248180	-0.8870510
H	5.0414780	0.0504280	-0.0001680
H	4.0225550	-1.1246400	0.8871680

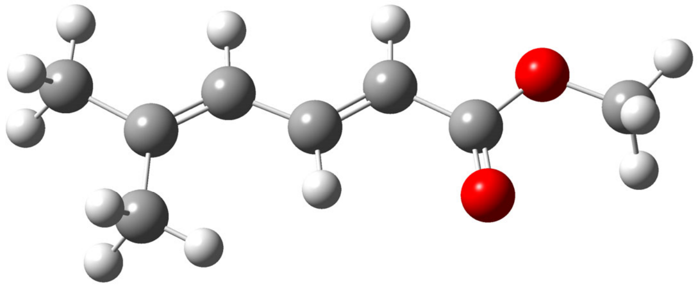

Supplementary Fig 31. The optimized structure of *E*-**2**.

Supplementary Table 5. Cartesian coordinates (in Å) of the optimized geometry of *E*-**2**.

Symbol	X	Y	Z
C	-3.3414370	-1.5161850	0.0000120
H	-3.9919440	-1.6178560	0.8771800
H	-2.6379550	-2.3498480	0.0000420
H	-3.9919330	-1.6178990	-0.8771560
C	-2.6407830	-0.1841210	-0.0000090
C	-3.5645860	1.0037780	-0.0000190
H	-4.2225960	0.9759820	-0.8763780
H	-4.2223640	0.9761900	0.8765210
H	-3.0533630	1.9654050	-0.0002030
C	-1.2905390	-0.1288060	-0.0000040
H	-0.7359320	-1.0596470	-0.0000010
C	-0.4931060	1.0771570	0.0000100
H	-1.0271880	2.0236600	0.0000200
C	0.8551960	1.1716640	0.0000170
H	1.3177820	2.1518530	0.0000340
C	1.7990380	0.0418280	0.0000070
O	1.5414260	-1.1430900	-0.0000030
O	3.0758670	0.5085170	0.0000030
C	4.0996780	-0.4940180	-0.0000150
H	4.0223580	-1.1248180	-0.8870510
H	5.0414780	0.0504280	-0.0001680
H	4.0225550	-1.1246400	0.8871680

Supplementary Table 6. Gibbs energy (in a.u.) of the optimized structures of **3-transoid-antifacial-HCN**, **3-cisoid-antifacial-HCN**, ***Z*-2**, and ***E*-2**.

**3-transoid-antifacial-HCN**	-1185.126340
**3-cisoid-antifacial-HCN**	-1185.123730
*Δ*G = G(**3-cisoid-antifacial-HCN**) – G(**3-transoid-antifacial-HCN**)	0.00261 (6.9 kJ/mol)
Z-**2**	-462.474232
E-**2**	-462.477399
*Δ*G = G(*Z*-**2**) – G(*E*-**2**)	0.003167 (8.3 kJ/mol)

X-ray crystallographic analyses

A crystal of suitable dimensions was mounted on a CryoLoop (Hampton Research Corp.) with a layer of paraton-N oil and placed in a nitrogen stream at 153(2) or 123(2) K. All measurements were performed on a R-AXIS RAPID II Imaging Plate detector with graphite-monochromated Mo-Kα (0.71075 Å) radiation. The structure was solved by direct methods (SIR92[27] or SHELXT-2014/5[28]) and refined on F^2 by full-matrix least-squares methods; using SHELXL-2014/1 or 2017/1.[29] Non-hydrogen atoms were anisotropically refined. H-atoms were included in the refinement on calculated positions riding on their carrier atoms. The ORTEP-3 program[30] was used to draw the molecule. Crystal data for the structures reported in this paper have been deposited in the Cambridge Crystallographic Database Center: CCDC 2003060 (**3-transoid-antifatial**), 2003061 (**13**), 2003062 (**17**).

X-ray crystallographic data

Crystal data for **3-transoid-antifacial**: $C_{18}H_{27}B_2F_8N_5O_2Pd_{13}$, M_r = 731.85, *monoclinic*, space group $P2_1/n$ (no. 14). a = 16.0562(9) Å, b = 9.7527(5) Å, c = 19.1775(9) Å, β = 105.3218(15)°, Z = 4, V = 2896.3(3) Å^3, F(000) = 1440, D_c = 1.678 g cm^{-3}, μ(Mo-Kα) = 13.17 cm^{-1}, T = 153 K, 21421 reflections collected, 5281 unique (R_{int} = 0.0556), 342 variables refined with 3983 reflections with $I > 2\sigma(I)$ to R = 0.0601. CCDC 2003060.

Crystal data for **13**: $C_{13}H_{24}BF_4N_3OPd$, M_r = 431.56, *orthorhombic*, space group $P2_12_12_1$ (no. 19). a = 7.90266(19) Å, b = 12.0143(4) Å, c = 19.1843(5) Å, Z = 4, V = 1821.44(8) Å^3, F(000) = 872, D_c = 1.574 g cm^{-3}, μ(Mo-Kα) = 10.61 cm^{-1}, T = 123 K, 40575 reflections collected, 4161 unique (R_{int} = 0.0347), 215 variables refined with 4091 reflections with $I > 2\sigma(I)$ to R = 0.0233. CCDC 2003061.

Crystal data for **17**: $C_{12}H_{16}BF_4N$, M_r = 261.07, *orthorhombic*, space group *Pnma* (no. 62). a = 10.7312(9) Å, b = 9.8162(7) Å, c = 10.9115(9) Å, Z = 4, V = 1149.41(15) Å^3, F(000) = 544, D_c = 1.509 g cm^{-3}, μ(Mo-Kα) = 1.32 cm^{-1}, T = 123 K, 23912 reflections collected, 1387 unique (R_{int} = 0.0442), 125 variables refined with 1039 reflections with $I > 2\sigma(I)$ to R = 0.0483. CCDC 2003062.

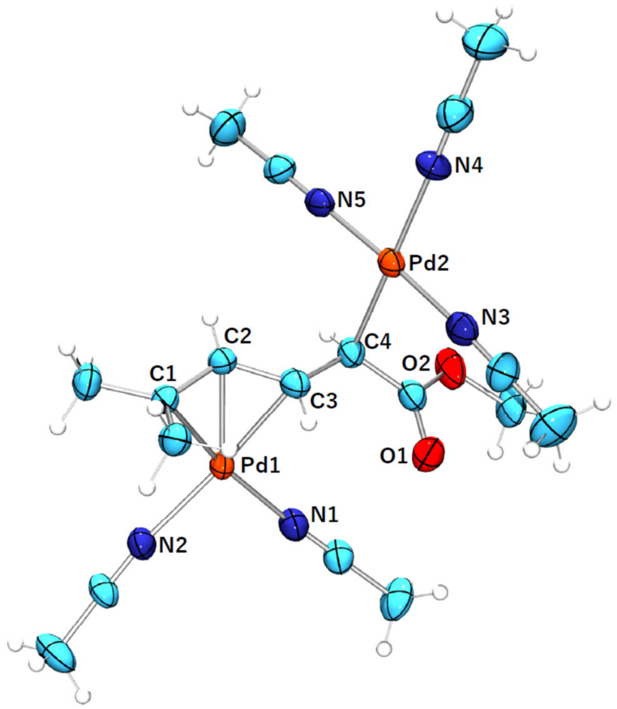

Supplementary Fig 32. An ORTEP of complex **3-transoid-antifacial** (30% probability ellipsoid, BF_4 anions are omitted for clarity).

Supplementary Table 7. Selected Bond Distances (Å) of Complex **3-transoid-antifacial**.

Pd1−N1	2.077(8)	Pd1−N2	2.090(6)
Pd1−C1	2.174(9)	Pd1−C2	2.096(7)
Pd1−C3	2.173(7)	Pd2−N3	2.002(7)
Pd2−N4	2.137(6)	Pd2−N5	2.005(7)
Pd2−C4	2.065(7)	C1−C2	1.413(11)
C2−C3	1.385(11)	C3−C4	1.446(12)

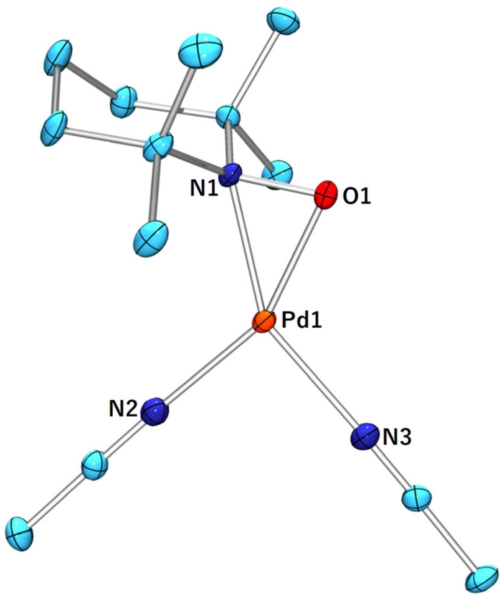

Supplementary Fig 33. An ORTEP of complex **13** (30% probability ellipsoid, BF_4 anions and protons are omitted for clarity).

Supplementary Table 8. Selected Bond Distances (Å) of Complex **13**.

Pd1−N1	2.041(2)	Pd1−N2	2.041(3)
Pd1−N3	2.073(3)	Pd1−O1	1.985(2)
N1−O1	1.359(3)		

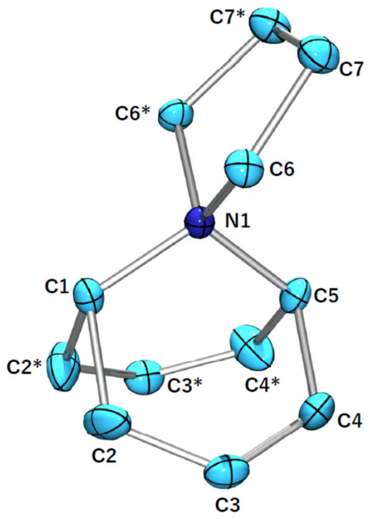

Supplementary Fig 34. An ORTEP of complex **17** (30% probability ellipsoid, BF_4 anions and protons are omitted for clarity).

Supplementary Table 9. Selected Bond Distances (Å) of Complex **17**.

N1–C1	1.492(3)	N1–C5	1.521(3)
N1–C6	1.518(2)	C1–C2	1.475(3)
C2–C2*	1.834(4)	C2–C3	1.410(3)
C3–C4	1.356(3)	C4–C4*	2.162(3)
C4–C5	1.481(3)	C6–C7	1.520(3)
C7–C7*	1.537(3)		

The structure of aza-barbaralane

The molecular structure of 9-aza-barbaralane **17** showed the longer bond length of C2–C2* (1.834(4) Å) and the shorter distance between C4···C4* (2.162(3) Å) compared to typical barbaralane or their 9-hetero analogues (**s1**-**s4**) (1.529(1)-1.592(2) Å and 2.359(2)-2.420(2) Å, respectively). This observation maybe caused by disorder of the C_8N skeleton.

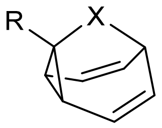

s1: R = Ar, X = COH(Ar)
(Ar = *p*-OMe-C_6H_4) (Ref. 31)
s2: R = H, X = CO (Ref. 32)
s3: R =H, X = BPh (Ref. 33)
s4: R =H, X = PPhO (Ref. 34)

Supplementary References

1. Murahashi, T., Nagai, T., Okuno, T., Matsutani, T., & Kurosawa, H. Synthesis and ligand substitution reactions of a homoleptic acetonitrile dipalladium(I) complex. *Chem. Commun.* 1689-1690 (2000).
2. Ukai, H., Kawazura, H., Ishii, Y., Bonnet, J. J. & Ibers, J. A. Chemistry of dibenzylideneacetone-palladium(0) complexes 1. Novel tris(dibenzylideneacetone)-dipalladium(solvent) complexes and their reactions with quinones. *J. Organomet. Chem.* **65**, 253–266 (1974).
3. Schwalbe, M., Walther, D., Schreer, H., Langer, J. & Görls, H. A new synthesis for thermolabile low-valent palladium complexes by electron transfer reactions from nickel(0) to palladium(II) compounds. *J. Organomet. Chem.* **691**, 4868–4873 (2006).
4. Hendrickson, D. N., Sohn, Y. S. & Gray, H. B. Magnetic susceptibility study of various ferricenium and iron(III) dicarbollide compounds. *Inorg. Chem.* **10**, 1559–1563 (1971).
5. Blumberg, L. C., Costa, B. & Goldstein, R. Chemoselective 1,3-dipolar cycloadditions of azomethine ylide with conjugated dienes. *Tetrahedron Lett.* **52**, 872–874 (2011).
6. Unelius, C. R., Liblikas, I. & Mozuraitis, R. Synthesis and characterization of the four geometrical isomers of 3,5-dodecadienyl acetate. *Acta Chemica Scandinavia* **52**, 930–934 (1998).
7. Pedersen, U., Rasmussen, P. B. & Lawesson, S.-O. Synthesis of naturally occurring curcuminoids and related compounds. *Liebigs Ann. Chem.* 1557–1569 (1985).
8. Taylor, S. D., Kotoris, C. C., Dinaut, A. N. & Chen, M.-J. Synthesis of aryl(difluoromethylenephosphonates) via electrophilic fluorination of α-carbanions of benzylic phosphonates with N-fluorobenzenesulfonimide. *Tetrahedron* **54**, 1691–1714 (1998)
9. González, Á., Aiguadé, J., Urpí, F. & Vilarrasa, J. Asymmetric acetate aldol reactions in connection with an enantioselective total synthesis of macrolactin A. *Tetrahedron Lett.* **37**, 8949–8952 (1996).
10. DeBoef, B., Counts, W. R. & Gilbertson, S. R. Rhodium-catalyzed synthesis of eight-membered rings. *J. Org. Chem.* 72, 799–804 (2007).

11. Gigant, N. & Bäckvall, J.-E. Synthesis of conjugated dienes via a biomimetic aerobic oxidative coupling of two C_{vinyl}–H bonds. *Chem. Eur. J.* **19**, 10799–10803 (2013).
12. Crouch, I. T., Dreier, T. & Frantz, D. E. Palladium-catalyzed elimination/isomerization of enol triflates into 1,3-dienes. *Angew. Chem. Int. Ed.* **50**, 6128–6132 (2001).
13. Hu, X.-H., Yang, X.-F. & Loh, T.-P. Selective alkenylation and hydroalkenylation of enol phosphates through direct C–H functionalization. *Angew. Chem. Int. Ed.* **54**, 15535–15539 (2015).
14. Lewis, F. D., Howard, D. K., Barancyk, S. V., & Oxman, J. D. Lewis acid catalysis of photochemical reactions. 5. selective isomerization of conjugated butenoic and dienoic esters. *J. Am. Chem. Soc.* **108**, 3016-3023 (1986).
15. Schabel, T. & Plietker, B. Microwave-accelerated Ru-catalyzed hydrovinylation of alkynes and enynes: a straightforward approach toward 1,3-dienes and 1,3,5-trienes. *Chem. Eur. J.* **19**, 6938–6941 (2013).
16. Lu, X. Huang, X. & Ma, S. A convenient stereoselective synthesis of conjugated (2Z)-ene-4-ynoic and (2Z,4Z)- and (2Z,4E)-dienoic acid derivatives from propiolic acid derivatives. *Tetrahedron Lett.* **33**, 2535–2538 (1992).
17. Zalesskiy, S. S. & Ananikov, V. P. $Pd_2(dba)_3$ as a precursor of soluble metal complexes and nanoparticles: determination of palladium active species for catalysis and synthesis. *Organometallics* **31**, 2302–2309 (2012).
18. Frisch, M. J., Trucks, G. W., Schlegel, H. B., Scuseria, G. E., Robb, M. A., Cheeseman, J. R., Scalmani, G., Barone, V., Mennucci, B., Petersson, G. A., Nakatsuji, H., Caricato, M., Li, X., Hratchian, H. P., Izmaylov, A. F., Bloino, J., Zheng, G., Sonnenberg, J. L., Hada, M., Ehara, M., Toyota, K., Fukuda, R., Hasegawa, J., Ishida, M., Nakajima, T., Honda, Y., Kitao, O., Nakai, H., Vreven, T., Montgomery, J. A., Peralta, Jr., J. E., Ogliaro, F., Bearpark, M., Heyd, J. J., Brothers, E., Kudin, K. N., Staroverov, V. N., Keith, T., Kobayashi, R., Normand, J., Raghavachari, K., Rendell, A., Burant, J. C., Iyengar, S. S., Tomasi, J., Cossi, M., Rega, N., Millam, J. M., Klene, M., Knox, J. E., Cross, J. B., Bakken, V., Adamo, C., Jaramillo, J., Gomperts, R., Stratmann, R. E., Yazyev, O., Austin, A. J., Cammi, R., Pomelli, C., Ochterski, J. W., Martin, R. L., Morokuma, K., Zakrzewski, V. G., Voth, G. A., Salvador, P., Dannenberg, J. J., Dapprich, S., Daniels, A. D., Farkas,

Ö., Foresman, J. B., Ortiz, J. V., Cioslowski, J. & Fox, D. J. *Gaussian 09, Revision D.01.* (Gaussian, Inc., Wallingford CT, 2013).

19. Becke, A. D. Density-functional exchange-energy approximation with correct asymptotic behavior. *Phys. Rev.* **A38**, 3098–3100 (1988).

20. Becke, A. D. Density-functional thermochemistry. III. The role of exact exchange. *J. Chem. Phys.* **98**, 5648–5652 (1993).

21. Vosko, S. H., Wilk, L. & Nusair, M. Accurate spin-dependent electron liquid correlation energies for local spin density calculations: a critical analysis. *Can. J. Phys.* **58**, 1200–1211 (1980).

22. Lee, C., Yang, W. & Parr, R. G. Development of the Colle-Salvetti correlation-energy formula into a functional of the electron density. *Phys. Rev.* **B37**, 785–789 (1988).

23. Miehlich, B. Savin, A., Stoll, H. & Preuss, H. Results obtained with the correlation energy density functionals of Becke and Lee, Yang and Parr. *Chem. Phys. Lett.* **157**, 200–206 (1989).

24. Andrae, D., Häußermann, U., Dolg, M., Stoll, H. & Preuß, H. Energy-adjusted *ab initio* pseudopotentials for the second and third row transition elements. *Theor. Chim. Acta* **77**, 123–141 (1990).

25. Krishnan, R., Binkley, J. S., Seeger, R. & Pople, J. A. Self-consistent molecular orbital methods. XX. A basis set for correlated wave functions. *J. Chem. Phys.* **72**, 650–654 (1980).

26. McLean, A. D. & Chandler, G. S. Contracted Gaussian basis sets for molecular calculations. I. Second row atoms, Z=11-18. *J. Chem. Phys.* **72**, 5639–5648 (1980).

27. Altomare, A., Cascarano, G., Giacovazzo, C., Guagliardi, A., Burla, M. C., Polidori, G. & Camalli, M. SIR92 - a program for automatic solution of crystal structures by direct methods. *J. Appl. Crystallogr.* **27**, 435 (1994).

28. Sheldrick, G. M. SHELXT – Integrated space-group and crystal-structure determination. *Acta Crystallogr.* **A71**, 3–8 (2015).

29. Sheldrick, G. M. Crystal structure refinement with SHELXL. *Acta Crystallogr.* **C71**, 3–8 (2015).

30. Farrugia, L. J. WinGX and ORTEP for Windows: an update. *J. Appl. Crystallogr.* **45**, 849–854 (2012).

31. Bismillah, A. N., Sturala, J., Chapin, B. M., Yufit, D. S., Hodgkinson, P. & McGonigal, P. R. Shape-selective crystallization of fluxional carbon cages. *Chem. Sci.* **9**, 8631–8636 (2018).

32. Ferre, S. & Echavarren, A. M. Synthesis of barbaralones and bullvalenes made easy by gold catalysis. *Angew. Chem. Int. Ed.* **55**, 11178–11182 (2016).

33. Herberich, G. E., Marx, H.-W., Moss, S., von Ragué Schleyer, P. & Wagner, T. 9-Borabarbaralanes. *Chem. Eur. J.* **2**, 458–461 (1996).

34. Gillick-Healy, M. W., Jennings, E. V., Müller-Bunz, H., Ortin, Y., Nikitin, K. & Gilheany, D. G. Two independent orthogonal stereomutations at a single asymmetric center: a narcissistic couple. *Chem. Eur. J.* **23**, 2332–2339 (2017).
